# Extracellular vesicles produced by irradiated endothelial or Glioblastoma stem cells promote tumor growth and vascularization modulating tumor microenvironment

**DOI:** 10.1186/s12935-024-03253-0

**Published:** 2024-02-12

**Authors:** Giorgia Castellani, Mariachiara Buccarelli, Quintino Giorgio D’Alessandris, Ramona Ilari, Andrea Cappannini, Francesca Pedini, Alessandra Boe, Valentina Lulli, Isabella Parolini, Stefano Giannetti, Mauro Biffoni, Vincenzo Zappavigna, Giovanna Marziali, Roberto Pallini, Lucia Ricci-Vitiani

**Affiliations:** 1 Department of Oncology and Molecular Medicine, Istituto Superiore di Sanità, Viale Regina Elena 299, 00161 Rome, Italy; 2 Fondazione Policlinico Universitario A. Gemelli IRCCS, Rome, Italy; 3 Institutes of Neurosurgery, Catholic University School of Medicine, Rome, Italy; 4 Sapienza, University of Rome, Rome, Italy; 5 Core Facilities, Istituto Superiore di Sanità, Rome, Italy; 6 Department of Medicine, University of Udine, Udine, Italy; 7 Institute of Human Anatomy, Università Cattolica del Sacro Cuore, Rome, Italy; 8 Department of Life Sciences, University of Modena and Reggio Emilia, Modena, Italy

**Keywords:** Glioblastoma, Glioblastoma stem cells, Extracellular vesicles, Endothelial cells, Tumor microenvironment, Periostin, Filamin-B

## Abstract

**Background:**

Glioblastoma (GBM) is the most lethal primary brain tumor in adult, characterized by highly aggressive and infiltrative growth. The current therapeutic management of GBM includes surgical resection followed by ionizing radiations and chemotherapy. Complex and dynamic interplay between tumor cells and tumor microenvironment drives the progression and contributes to therapeutic resistance. Extracellular vesicles (EVs) play a crucial role in the intercellular communication by delivering bioactive molecules in the surrounding milieu modulating tumor microenvironment.

**Methods:**

In this study, we isolated by ultracentrifugation EVs from GBM stem-like cell (GSC) lines and human microvascular endothelial cells (HMVECs) exposed or not to ionizing irradiation. After counting and characterization, we evaluated the effects of exposure of GSCs to EVs isolated from endothelial cells and vice versa. The RNA content of EVs isolated from GSC lines and HMVECs exposed or not to ionizing irradiation, was analyzed by RNA-Seq. Periostin (POSTN) and Filamin-B (FLNB) emerged in gene set enrichment analysis as the most interesting transcripts enriched after irradiation in endothelial cell-derived EVs and GSC-derived EVs, respectively. POSTN and FLNB expression was modulated and the effects were analyzed by in vitro assays.

**Results:**

We confirmed that ionizing radiations increased EV secretion by GSCs and normal endothelial cells, affected the contents of and response to cellular secreted EVs. Particularly, GSC-derived EVs decreased radiation-induced senescence and promoted migration in HMVECs whereas, endothelial cell-derived EVs promoted tumorigenic properties and endothelial differentiation of GSCs. RNA-Seq analysis of EV content, identified FLNB and POSTN as transcripts enriched in EVs isolated after irradiation from GSCs and HMVECs, respectively. Assays performed on POSTN overexpressing GSCs confirmed the ability of POSTN to mimic the effects of endothelial cell-derived EVs on GSC migration and clonogenic abilities and transdifferentiation potential. Functional assays performed on HMVECs after silencing of FLNB supported its role as mediator of the effects of GSC-derived EVs on senescence and migration.

**Conclusion:**

In this study, we identified POSTN and FLNB as potential mediators of the effects of EVs on GSC and HMVEC behavior confirming that EVs play a crucial role in the intercellular communication by delivering bioactive molecules in the surrounding milieu modulating tumor microenvironment.

**Supplementary Information:**

The online version contains supplementary material available at 10.1186/s12935-024-03253-0.

## Background

Glioblastoma (GBM) is the most lethal brain cancer, with median survival of 12–15 months. Currently, the standard of care treatment of newly diagnosed GBM includes maximal surgical resection of the tumor and fractionated radiation therapy concurrent with alkylating agent, temozolomide (TMZ), chemotherapy and offers an improvement in median survival to only 13.4–19 months [1, 2]. Unfortunately, the response to radiotherapy is not consistent for all patients and radiotherapy resistance frequently occurs contributing to aggressive growth and almost inevitable, recurrence. Despite extensive studies on GBM resistance to radiotherapy, its mechanisms are still largely not understood because of the multifactorial and heterogeneous nature of this phenomenon. Many factors have been involved in GBM resistance to radiotherapy: intra-, inter-tumor heterogeneity and tumor plasticity, aberrant expression of non-coding RNAs (ncRNAs), metabolic and epigenetic reprogramming, alterations in DNA repair and cell cycle [3]. Among these, the existence of a rare population of self-renewing stem-like cells able to sustain tumor initiation and growth, to contribute to therapy resistance and recurrence of the disease [4]. Several in vitro and in vivo studies demonstrated that GBM stem-like cells (GSCs) are extremely resistant to most common therapies, including radiation and TMZ [5, 6]. We analysed a large panel of GSC lines and related the resistance to radiation and TMZ with survival of donor patients, confirming the translational power of patient-derived GSC cultures in predicting treatment response [7].

GSCs are localized in perivascular niches in close proximity to brain microvascular endothelial cells where they are involved in bidirectional molecular and cellular interactions [8]. The vasculature is an important component of GSC niches with a critical function of supporting GSC proliferation and regeneration [9]. On the other hand, several studies demonstrate that GSCs might modulate endothelial cell behavior and plasticity to foster tumor vascular development [10, 11].

Angiogenesis is a prominent feature of GBM and it is mainly due to the ability of tumor cells to stimulate the formation of new blood vessels driven by angiogenesis factors. GBM can adopt various strategies to build up its own vascularity. Among these strategies, neo-angiogenesis, i.e., the sprouting from pre-existing brain vessels, is commonly regarded as the main one [12]. In GBM recurring after radiotherapy, however, neo-angiogenesis may lose efficiency due to the radiation-induced senescence of the brain endothelium [13].

GBM tumor microenvironment (TME) is a highly heterogeneous and complex system, which in addition to cancer cells, consists of various resident brain and immune cells, as well as, stroma, blood vessels, vascular component and infiltrating inflammatory cells [14]. Emerging evidence suggests that GBM cells, particularly the GSC population, appear to respond and adapt to the surrounding TME to promote rapid proliferation, invasion, migration, and survival, thus generating treatment resistance [15, 16]. GBM cells interact with their microenvironment directly through cell-to-cell mechanism by interaction mediated by cell-surface molecules, or indirectly through apocrine or paracrine soluble factors, diffusing through the extracellular matrix, or signaling molecules delivered by extracellular vesicles (EVs), being the latter identified as an essential means of bidirectional communication between tumors and TME [17].

EVs are a heterogeneous group of membrane-secreted vesicles, which, based on their size, density, and mechanism of biogenesis, are sorted into three main types: exosomes (30–100 nm in diameter, endocytic origin), microvesicles (50–1000 nm in diameter, outward budding of the plasma membrane), and apoptotic bodies (> 1000 nm in diameter, cell fragmentation during apoptotic cell death) [18]. Moreover, accumulating data have indicated that the content, size and membrane composition of EVs are highly heterogeneous and dynamic and depend on the cellular source, state and environmental conditions.

We have previously shown that GSCs possess potentials for mesenchymal differentiation and that these cells can form functional networks of vascular channels both in vitro and in vivo [19, 20]. EVs are secreted at low levels by normal endothelial cells (ECs) but their secretion might be increased under cell stressing conditions, like radiation. In the vascular niche of irradiated brain, a symbiotic relationship might be hypothesized, where the GBM cell allows the endothelial cell to escape from radiation-induced senescence and the endothelial cell provides differentiation cues to the tumor cell driving its contribution to the angiogenic process.

## Methods

### Cell cultures

GSCs were isolated from surgical samples of adult patients who underwent craniotomy at the Institute of Neurosurgery, Catholic University of Rome, upon approval by the local ethical committee. Informed consent was obtained from the patients before surgery. After mechanical dissociation, single cell suspension was cultured in a serum-free medium supplemented with 20 ng/ml epidermal growth factor (EGF, PeproTech, London, UK) and 10 ng/ml basic fibroblast growth factor (b-FGF, PeproTech), as previously described [19, 21]. GSC lines were validated by Short Tandem Repeat (STR) DNA fingerprinting. Nine highly polymorphic STR loci plus amelogenin (Cell ID^™^ System, Promega Inc., Madison, WI, USA) were used and all GSC profiles were challenged against public databases to confirm authenticity [22].

Human microvascular endothelial cells (HMVECs) were purchased from Lonza and cultured in endothelial basal medium (EBM-2, Lonza Walkersville Inc., Walkerswille, MD, USA) supplemented with EGM^™^-2 MV SingleQuots™ Kit (Lonza Walkersville Inc.), at 37 °C in 5% CO_2_ atmosphere.

Packaging cell line, 293 T, was maintained in DMEM (Euroclone, Pero, MI, Italy) supplemented with 10% (v/v) heat-inactivated FBS, 2 mM L-glutamine, 100 U/ml of penicilline and 100 µg/ml of streptomycin (Invitrogen by Life Technologies, Carlsbad, CA, USA).

All the used cell lines were tested for mycoplasma contamination by using MycoAlert® Mycoplasma Detection Kit (Lonza Walkersville Inc.).

### Extracellular vesicle isolation, counting and tracking analysis

EVs were isolated by ultracentrifugation [23]. Cell media were clarified of cells and cellular debris by spinning media at 1400 rpm for 10 min, then at 3200 rpm for 20 min at 4 °C, before pelleting at 100,000×*g* for 3 h at 4 °C. EVs were washed in PBS and re-pelleted by an additional spinning at 100,000×*g* for 3 h at 4 °C. EVs were resuspended in PBS. EV count was performed using Gallios flow cytometer (Beckman Coulter Life Sciences, Indianapolis, IN, USA). Size of EVs was directly tracked using the Nanosight NS300 system (Nanosight™ technology, Malvern, UK), configured with a 488 nm laser and a high-sensitivity sCMOS camera. Videos were collected and analyzed using the NTA software (version 3.0). For each sample, multiple videos of 60 s duration were recorded generating replicate histograms that were averaged.

### Generation of fluorescent EVs (C16-EVs) and cell uptake

C16-EVs were generated using the green fluorescent fatty acid BODIPY^®^ FL C_16_ (Thermo Fisher Scientific, Waltham, MA, USA). Briefly, to allow the incorporation of the fluorescent molecule as phospholipid into EV membrane, cells were incubated with 10 μM BODIPY for 6 h at 37 °C in 5% CO_2_ atmosphere. Labeling was stopped washing with PBS to remove excess BODIPY. EVs were isolated from cell conditioned media (40 h) by ultracentrifugation and counted by FACS analysis as reported [23].

To verify labeled EV uptake, 4 × 10^4^ cells were incubated with EVs (200 EVs/cell) for 1 h at 37 °C using a thermoblock. Cells were analyzed using the flow cytometer FACSCanto (BD Biosciences, Milan, Italy).

### Exposure of cells and mouse brain to radiations

HMVECs at 70% to 80% confluence or GSCs at density of 1.2 × 10^5^ cells/ml were exposed to single doses of acute cesium-137 (^137^Cs) γ irradiation (10 Gy and 50 Gy). Dose rate was 0.7 Gy/min. Media were replaced 4 h after irradiation, using exosome-depleted serum for HMVECs (System Biosciences, Palo Alto, CA, USA). Cells were incubated for 40 h at 37 °C in 5% CO_2_ atmosphere before EV isolation. Animal experiments were approved by the Ethical Committee of the Catholic University of Rome, Italy. CD1 mice (male, 25–30 g; Charles River, Milan, Italy) were anesthetized with intraperitoneal injection of diazepam (2 mg/100 g) followed by intramuscular injection of ketamine (4 mg/100 g). Mice skulls were immobilized in a custom-made head holder with a lead mask covering both eyes and nose and then exposed to 6 Gy dose of ^137^Cs γ irradiation at 0.7 Gy/min rate, as previously described [24]. Briefly, radiation was administered twice a week over 2 weeks (total dose 24 Gy). After 2 weeks, the mice were deeply anesthetized and transcardially perfused with 0.1 M PBS (pH 7.4) then treated with 4% paraformaldehyde in 0.1 M PBS. The brain was removed, stored in 30% sucrose buffer overnight at 4 °C, and serially cryotomed at 20 μm on the coronal plane.

### Automated capillary Western immunoassay (WES)

Total cell and EV lysates were extracted by using Nonidet-P40 lysate buffer (1% NP40, 200 nM NaCl, 50 mM Tris pH 7.4) plus protease and phosphatase inhibitors, maintained on ice for 30 min, vortexed and then centrifuged at 12,000 rpm for 10 min at 4 °C. Protein concentration was measured by BioRad protein-assay (Hercules, CA, USA). Analysis of protein expression was performed using capillary WES^™^ technology (ProteinSimple by Bio-Techne, Minneapolis, MN, USA) according to the manufacturer’s protocol. Each sample was mixed with fluorescent 5XMaster Mix, incubated at 95 °C for 5 min and then loaded into a WES 12–230 kDa prefilled plate, along with a biotinylated protein ladder, blocking buffer, primary antibodies, ProteinSimple HRP-conjugated anti-rabbit or anti-mouse secondary antibody, luminol-peroxide, and washing buffer. Immunodetection was performed automatically, and the results are reported as virtual gels based on chemiluminescence signals. Compass software (ProteinSimple by Bio-Techne) was used to acquire and analyze the data and generate gel images and chemiluminescence intensities. The following primary antibodies were used:

Alix (NBP1-49,701, 1:10), Flotillin-1 (NBP1-79,022, 1:100) and Calnexin (NB100-1974, 1:50) from Novus Biologicals by Bio-Techne; CD9 (#13,174, 1:5, Cell Signaling, Danvers, MA, USA), Annexin A1 (MAB37701, 1:10, R&D Systems by Bio-Techne), Periostin (TA804575s, 1:10, OriGene, Rockville, MD, USA), Filamin-B (sc-376241, 1:20, Santa Cruz Biotechnology Inc., Dallas, TX, USA), β-actin (A5441, 1:100) and vinculin (V9131, 1:100) from Sigma-Aldrich Inc.

### RNA-Sequencing analysis on extracellular vesicles

To characterize EV RNA composition, total RNA was extracted from pelleted EVs using the miRCURY™ RNA Isolation Kit (Exiqon, Vedbaek, Denmark) according to the manufacturer’s instructions. RNA-Seq was performed using SMARTer^®^ Small RNA-Seq Kit and SMARTer^®^ Stranded Total RNA-Seq Kit—Pico Input Mammalian (Clontech Laboratories, Mountain View, CA, USA), according to the manufacturer’s instructions.

### Plasmid constructs, lentivirus infection and transient transfection

For Periostin (POSTN) ectopic expression and silencing experiments, POSTN-GFP (RC227934L4) and sh-POSTN-GFP (TL310280) lentiviral vectors were purchased from OriGene.

Lentiviral particles were produced in the packaging cell line 293 T (at 70–80% confluency) using the calcium phosphate transfection protocol and infection was performed as previously described [25].

After infection, GFP, POSTN-GFP, sh-NTC-GFP (no target control) and sh-POSTN-GFP transduced cells were analyzed and GFP fluorescence was assessed by FACSCanto (BD Biosciences). Then, GFP-positive cells were flow sorted by FACS ARIA (BD Biosciences).

For transient transfection, 10 pmol of siRNAs targeting the human Filamin-B (Filamin-B siRNA, si-FLNB: sc-60641; Santa Cruz Biotechnology Inc.) and a scrambled (siRNA-A control, si-CTRL: sc-37007; Santa Cruz Biotechnology Inc.) sequence, were used to transfect HMVECs by Lipofectamine 2000 (Invitrogen) according to the manufacturer’s protocol.

### RNA Extraction and Real-Time RT-PCR

Total RNA was isolated from cells and EVs using TRIzol reagent (ThermoFisher Scientific) and the miRCURY™ RNA Isolation Kit (Exiqon) respectively, according to the manufacturer’s instructions.

Real-time PCR for transcripts (GEM, MCM7, PMEPA1, POSTN, CENPJ, CNTROB, FLNB and LMNB) identified by RNA-Seq analysis were performed with SYBR^™^ Green Master Mix in StepOnePlus™ Real-Time PCR System (Applied Biosystems^™^ by ThermoFisher Scientific) and normalized with GAPDH using the following primers:

**Table:** 

	For: 5ʹ—3ʹ	Rev: 3ʹ—5ʹ
GAPDH	ACCTGACCTGCCGTCTAG	CCTGCTTCACCACCTTCT
GEM	GCATGGACAGCGACTGCGAG	CTGGATTCGCAGCTCAGATGC
MCM7	TCAATTTGTGAGAATGCCAGGCGC	CACAGTTACCAACTTCCCCACAGA
PMEPA1	TTTCCATCTCCTTTCCCCGC	CCCGCCAACCCCAAATCTAT
POSTN	TATCCAGCAGACACACCTGTT	TGACTTTTGTTAGTGTGGGTC
CENPJ	AGCCACTTGAACCAC	CTTATAAACCTTTTC
CNTROB	AGAGCCAGCATTCTTTCCAGC	TCAGGGAATGGCCTTTCTGCC
FLNB	ACACCAAAGCTGCAGGAAGT	GGCTCTTTGGAATGTGGTGT
LMNB	TCGCAAAAGCATGTATGAAGA	CTCTACCAAGCGCGTTTCA

### Senescence assays

For the senescence detection by immunofluorescence, expression of γ-H2A.X was assessed in HMVECs using a primary antibody (S139; rabbit monoclonal, 1:250, Cell Signaling). For the detection of senescence-associated β-galactosidase (β-gal) activity, HMVECs and brain sections were analysed by immunohistochemistry following the manufacturer’s instructions (#KAA0022RF, Millipore, Temecula, CA, USA). For these experiments, HMVECs were exposed to 50 Gy radiation and analyzed after 96 h.

For CD31 immunostaining of brain sections, slices were incubated with rabbit polyclonal anti-CD31 (1:50; Santa Cruz Biotechnology Inc.), followed by avidin–biotin peroxidase complex (ABC method, Vector Laboratories, Burlingame, CA, USA) and 3,3’-diamidinobenzidine (Sigma Aldrich Inc.) as a chromogen.

For the senescence detection by flow cytometry, a Senescence Assay Kit was purchased from Abcam (Ab228562, Abcam, Cambridge, UK) and the evaluation of β-gal activity in senescent cells were performed according to the manufacturer’s instructions. Briefly, cells were exposed to single dose of acute ^137^Cs β irradiation (50 Gy), then incubated with GSC-derived EVs for 72 h. The senescent dye was added and incubated for 2 h at 37 °C. Senescent-positive cells were detected with the flow cytometer FACSCanto (BD Biosciences) in the FL1 channel.

For the experiments of HMVECs with EVs, all the experiments were performed after incubation of HMVECs with EVs (1000 EVs/cell) for 1 h at 37 °C using a thermoblock.

### Scratch-wound migration assay

Migration ability of endothelial cells was evaluated by scratch-wound assay. HMVECs or transiently transfected HMVECs were grown to confluence, and a strip of cells was scratched at the centre of the well. Medium was replaced with fresh starvation medium without serum (EBM-2 with EGM™-2 MV SingleQuots™ Kit 1%) and GSC-derived EVs were added (1000 EVs/cell). After imaging acquisition using Axiovert A1 KMAT Zeiss microscope (Zeiss Italy, Milan, Italy), the area of the scratch was measured at 0 and 24 h to determine wound closure by using microscope software analysis (Zeiss Zen 3.6). The migration rate was quantified by the *area method* as the percentage wound area at a specific time point [26].

### Cell growth, migration, colony formation and transdifferentiation assay

For viability assay, GSC#61, GSC#61 incubated with HMVEC-derived EVs or with irradiated HMVEC-derived EVs and GSC#61 transduced with GFP or POSTN GFP and sh-NTC-GFP or sh-POSTN GFP vectors were plated at a density of 2 × 10^4^ cells/ml in 96-well plates in triplicate. Cell viability was monitored by using the CellTiter-Blue^™^ Viability Assay (Promega Inc.).

The migration ability of GSCs incubated with HMVEC-derived EVs or with irradiated HMVEC-derived EVs or transduced cells was evaluated by plating in Corning FluoroBlok™ Multiwell Inserts System (Corning Life Sciences, Tewksbury, MA, USA), according to the manufacturer's instructions. Briefly, 3 × 10^4^ cells were added to the upper chamber in stem cell medium without growth factors. Stem cell medium supplemented with growth factors (EGF and b-FGF) was used as chemoattractant in the lower chamber. The plates were incubated for 72 h at 37 °C, after which the fluorescent dye calcein acetoxymethylester (calcein AM, Life Technologies Corporation) was added to the lower chamber and incubated for 30 min at 37 °C. The cell viability indicator calcein AM is a non-fluorescent, cell permeant compound that is hydrolyzed by intracellular esterases into the fluorescent anion calcein and can be used to fluorescently label viable cells before microscope observation. The number of migrated cells was evaluated by counting the cells after imaging acquisition using a fluorescence microscope.

Colony formation ability was evaluated by plating a single cell/well in 96 well plates. After 3–4 weeks, each well was examined and the number of spheres/cell aggregates were counted.

In vitro transdifferentiation of GSCs incubated with HMVEC-derived EVs or with irradiated HMVEC-derived EVs or transduced GSCs was performed by culturing GSC tumorspheres in endothelial cell conditions for 2 weeks, as previously described [27]. For the evaluation of CD34 expression, cells were incubated for 90 min at 4 °C with the antibodies, then washed with PBS and analyzed by the flow cytometer FACSCanto (BD Biosciences). The antibodies used were as follows: anti-CD34-phycoerytrin antibody (R7125, 1:20, clone BIRMA-K3, DakoCytomation, Denmark) or PE-conjugated mouse IgG_1_ isotype control antibody (1:25, Miltenyi Biotec Inc., Bergisch Gladbach, Germany). Data were analyzed with FACS Diva software (Becton Dickinson).

For the experiments of GSCs with EVs, all the experiments were performed after incubation of GSCs with EVs (1000 EVs/cell) for 1 h at 37 °C using a thermoblock.

### Bioinformatic and statistical analyses

Data were analyzed with homemade Python scripts. We used os, sklearn, pandas, and seaborn Python libraries. The matrix was normalized along the rows to compare the values in the cell lines. We filtered the data set considering a log-fold change of 4. Multivariate analysis was performed by sklearn preprocessing and sklearn decomposition methods. Data clustering was done by seaborn package. A single linkage method was used.

Comparisons between two groups were performed using the unpaired Student’s t test. *P* < 0.05 was considered to indicate a statistically significant difference and the level of significance is indicated in the plots using asterisks as follows: * for p < 0.05, ** for p < 0.01 and *** for p < 0.001.

## Results

### GSC-derived EVs decrease radiation-induced senescence in HMVECs and promote their motility.

**Fig. 1:**
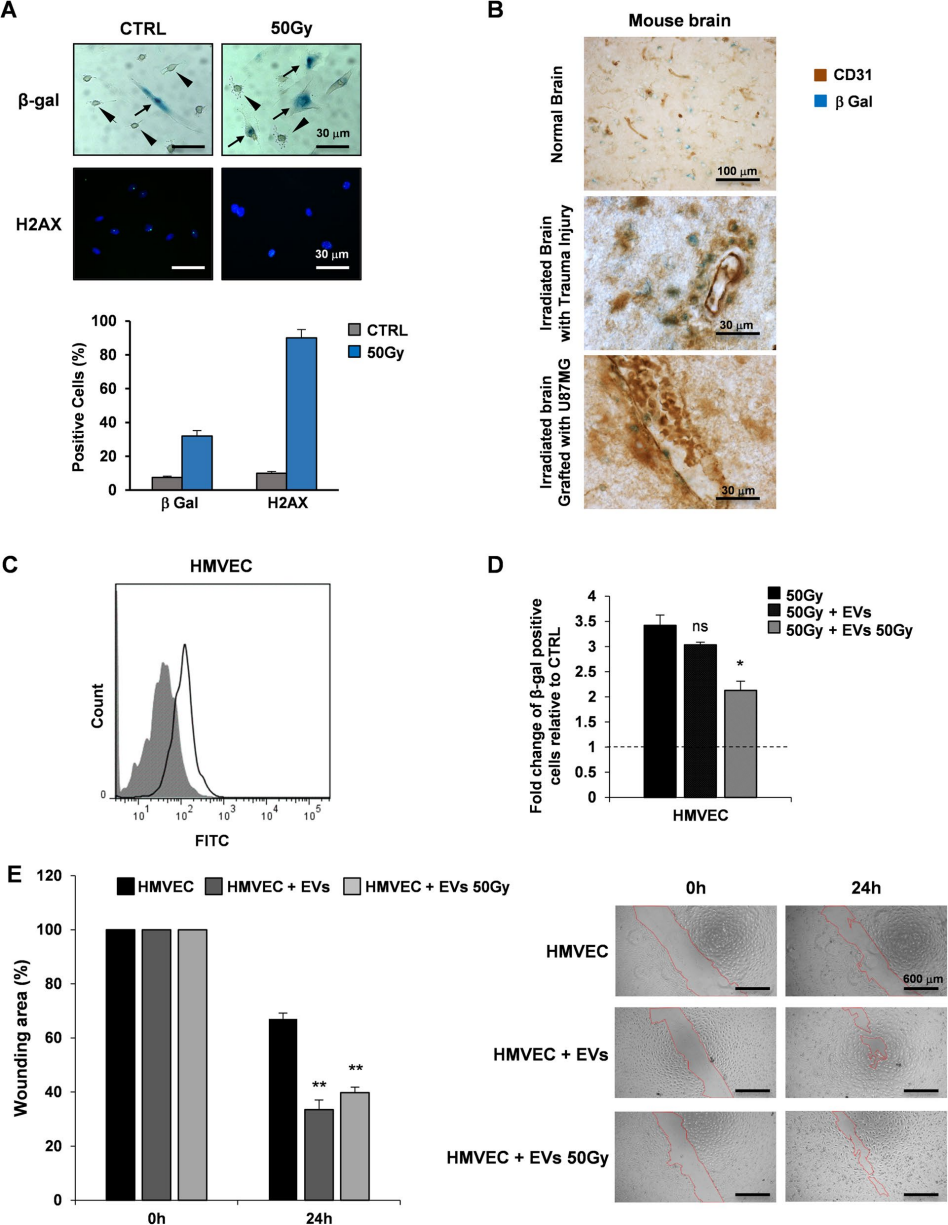
EVs isolated from GSCs decrease radiation-induced senescence and promote motility in HMVECs. **A**. Evaluation of β-gal and H2AX positive irradiated HMVECs (50 Gy) by immunohistochemistry (*up*) and immunofluorescence (*down*), respectively. The arrows point out senescent endothelial cells; arrowheads point out β-gal negative cells. Quantification of positive cells was shown (*lower panel*). Scale bar: 30 μm **B**. Immunohistochemical evaluation of CD31 and β-gal positive cells in normal mouse brain (*upper panel*), irradiated mouse brain with trauma injury (*center panel*) and irradiated mouse brain grafted with U87MG cells (*lower panel*). Scale bar: 100 μm *upper panel*, 30 μm *center and lower panels*. **C** FACS analysis based on green fluorescence of HMVECs after GSC-derived C16-EV uptake (shaded histogram = HMVEC control sample; open histogram = HMVECs incubated with EVs). **D** Measurement of β-gal positive irradiated HMVECs (50 Gy) relative to sham irradiated cells (CTRL, dashed line at value 1). HMVECs (50 Gy), HMVECs incubated with GSC-derived EVs (50 Gy + EVs) and HMVECs incubated with irradiated GSC-derived EVs (50 Gy + 50 Gy EVs) are shown. Results are shown as mean ± SD from two independent experiments. ns, not significant; *p < 0.05 *vs* HMVECs 50 Gy (Student’s t test). **E**. Scratch-wound assay quantification (*left*) and images (*right*) of HMVECs, HMVECs incubated with GSC-derived EVs and HMVECs incubated with irradiated GSC-derived EVs at 0 h and 24 h time points. Values are reported as the percentage wound area and shown as mean ± SD from two independent experiments. **p < 0.01 *vs* HMVECs (Student’s t test) (scale bar: 600 μm; magnification 4X)

Radiation provokes senescence in several types of cultured human endothelium, including the brain endothelium [24]. After γ-ray exposure (50 Gy), the majority of human endothelial cells undergo accelerated senescence, as indicated by elevated senescence-associated β-galactosidase (β-gal) with only a low population showing apoptosis (Fig. 1A, *up*). In cultured endothelial cells, expression of H2AX was much higher after radiation (Fig. 1A, *down*). In sham irradiated conditions, 7–8% of cultured endothelial cells showed β-gal positivity and increased up to fourfold after exposure to radiations. The effects of 50 Gy radiation on H2AX expression resulted in a ninefold increase in irradiated cells compared to sham irradiated cells (Fig. 1A, *lower panel*). Immunohistochemical evaluation of irradiated mouse brain sections showed that the expression of β-gal in endothelial CD31 positive cells in mouse brain grafted with U87MG cells is lower than those with trauma injury (Fig. 1B). These observations extend our previous studies where GBM cell conditioned medium prevented replicative senescence by cultured human umbilical vein endothelial cells (HUVECs) [28], confirming that soluble factors released by GBM cells may influence the local environment in such a way to favor tumor growth and vascularization.

To analyze the effect of GSC-derived EVs on endothelial cells, we first purified and analyzed EVs released by GSC#61 as a representative cell line of a collection of patient-derived GSCs, previously characterized [22]. Tumor cell derived EVs were isolated by ultracentrifugation from GSC conditioned medium. Nanoparticle tracking analysis (NTA) showed the EV population with a median size of 143.3 ± 2.1 nm and a mode of 140.5 ± 16.8 nm (Additional file 1: Fig. S1A). Common EV marker proteins like Alix, Flotillin-1, CD9 and Annexin A1 were found expressed (Additional file 1: Fig. S1B). The endoplasmic reticulum-associated protein calnexin was absent in EVs and observed only in cellular protein lysates thus confirming the purity of EV sample preparations [29].

To confirm the GSC-derived EV uptake by HMVECs, endothelial cells were incubated for 1 h with purified C16-EVs from GSCs, and fluorescence gain was measured by flow cytometry (Fig. 1C).

We then analyzed the effect of irradiation on the release of EVs in four GSC lines. EVs were isolated from culture media 40 h after single doses γ irradiation at 10 Gy and 50 Gy. A dose-related increase in the release of EVs was observed in all the irradiated GSC lines (Additional file 1: Fig. S1C). NTA measured EVs was not significantly influenced by irradiation (Additional file 1: Fig. S1D).

Next, we assessed whether EVs released by GSCs under stressing condition, may influence the local environment allowing the endothelial cell to escape from radiation-induced senescence. EVs were purified from 50 Gy irradiated or sham irradiated GSC#61 and incubated with 50 Gy irradiated HMVECs for 72 h. As shown in Fig. 1D, β‐gal activity significantly decreased in irradiated HMVECs incubated with EVs derived from irradiated GSC#61 compared to 50 Gy irradiated HMVECs without EVs, suggesting the potential of EVs released by GSCs under stress condition to protect endothelial cells from radiation-induced senescence.

Finally, we verified whether EVs released by GSCs after radiation exposure, may influence migration ability of HMVECs in a scratch-wound assay. In HMVECs, incubation with EVs purified from 50 Gy irradiated or sham irradiated GSC#61, significantly increased wound closure after 24 h compared to control conditions (without EVs) (Fig. 1E).

### Endothelial cell-derived EVs promote tumorigenic properties and endothelial transdifferentiation of GSCs

**Fig. 2:**
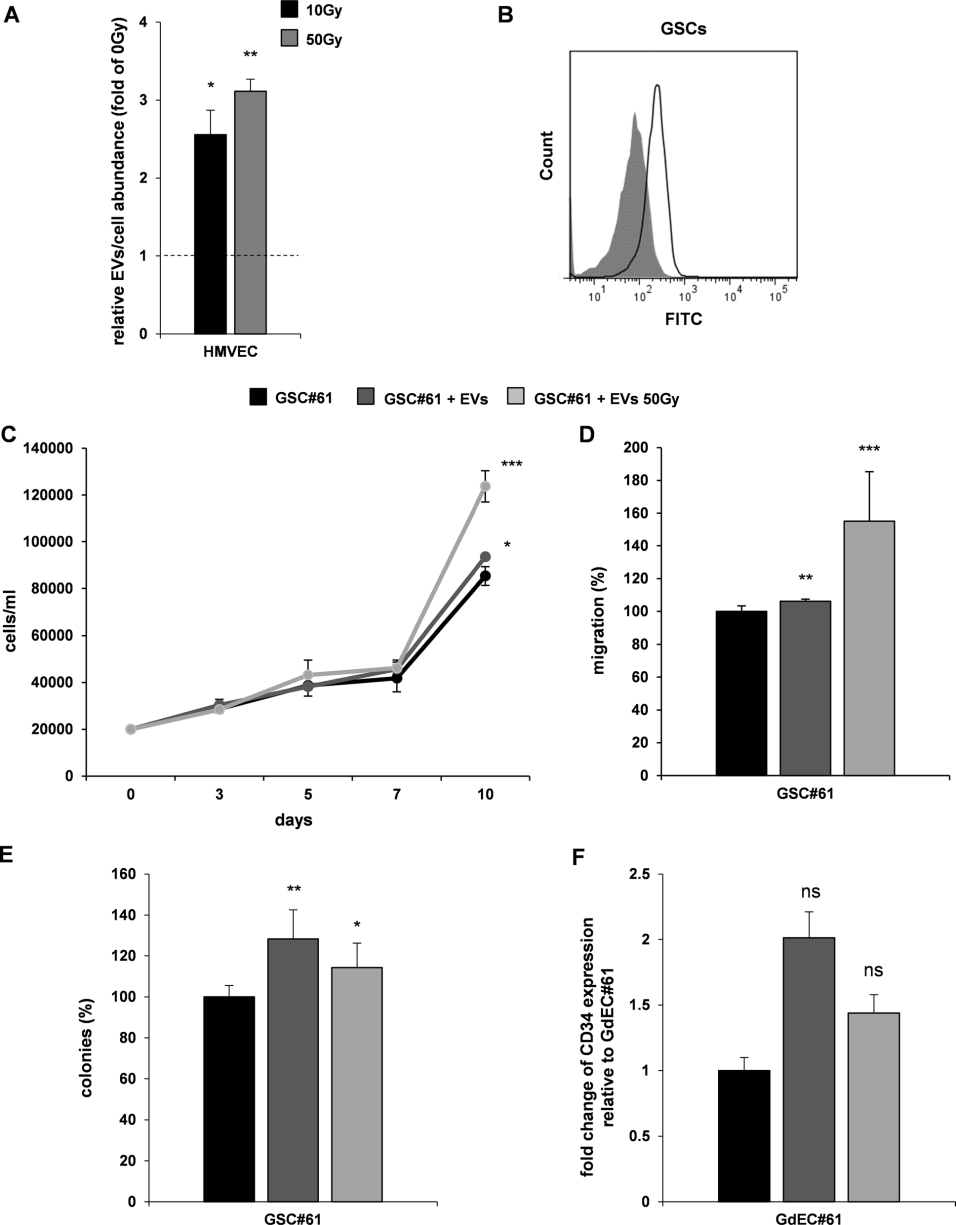
HMVEC-derived EVs promote GSC tumorigenic properties and endothelial transdifferentiation potential. **A**. Measurement of EVs/cell released by irradiated HMVECs (10 Gy and 50 Gy) relative to sham irradiated cells, used as control (0 Gy, dashed line at value 1). Results are reported as mean ± SD (n = 3). **B**. FACS analysis based on green fluorescence of GSCs after HMVEC-derived EV uptake (shaded histogram = GSC control sample; open histogram = GSCs incubated with EVs). **C**–**E**. Functional in vitro assays on GSC#61, GSC#61 incubated with HMVEC-derived EVs (EVs) and GSC#61 incubated with irradiated HMVEC-derived EVs (50 Gy EVs). Growth curve **C**: points and range lines at each day represent mean ± SD of at least two independent experiments in triplicate. **D**. Migration assay: values are reported as percentage relative to GSC#61 and shown as mean ± SD from two independent experiments in triplicate. **E**. Colony formation assay: percent colony number values from two independent experiments in triplicate were calculated over GSC#61 and are shown as mean ± SD. *p < 0.05; **p < 0.01; ***p < 0.001 *vs* GSC#61 without EVs (Student’s t test). **F** Transdifferentiation assay: cytofluorimetric evaluation of CD34 expression. Values are reported as fold change relative to GSC-derived endothelial cells (GdEC) #61. ns, not significant

EVs are secreted by cells in the extracellular space both in physiologic conditions [30] and under several stimuli such as hypoxia, stress, senescence, cell death, and particularly, inflammation [31]. By using the same approach described for GSCs, we isolated EVs derived from HMVECs, either irradiated (10 Gy and 50 Gy) or sham irradiated. As observed for GSCs, after irradiation endothelial cells showed a significant increase of the number of EVs released per cell compared to sham irradiated cells (Fig. 2A), confirming a dose-related increase in EV release under stress conditions. To verify that endothelial cell-derived EVs could be transferred to a recipient cell, C16-EVs were incubated with GSCs and the uptake was confirmed by cytofluorimetric analysis, where fluorescence gain was observed (Fig. 2B). In order to evaluate the potential effects of endothelial cell-derived EVs on GSC tumorigenic properties in vitro, GSC#61 were incubated with EVs isolated from either sham irradiated or 50 Gy-irradiated HMVECs and functional assays were performed (Fig. 2C–E). Interestingly, endothelial cell-derived EVs were able to significantly promote proliferation, migration and clonogenic abilities of GSCs. Furthermore, under endothelial culture conditions, we observed an increase of CD34 expression in GSCs cultured with HMVEC-derived EVs, suggesting an enhancement of endothelial transdifferentiation ability of GSCs (Fig. 2F) [27]. Our results showed that endothelial cell-derived EVs promote tumorigenic properties of GSCs either in normal or irradiated condition.

### Filamin-B and periostin transcripts are enriched in EVs isolated after irradiation

**Fig. 3:**
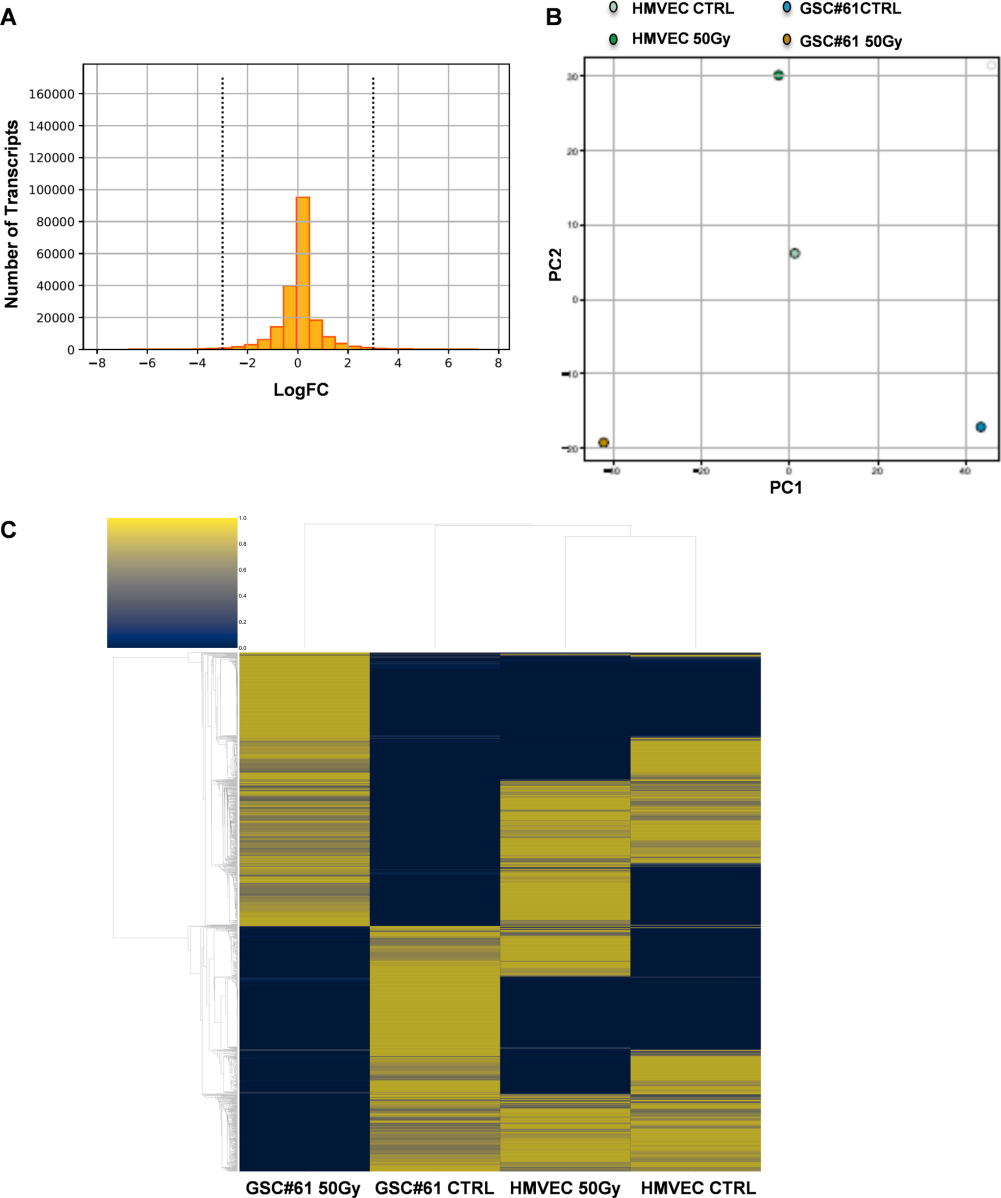
RNA-Seq of EV content and differential gene expression analysis. **A**. Histogram of the log fold change (logFC) of the genes. Vertical lines represent the upper and lower limits chosen as the fold change threshold. **B**. PCA applied to the cell lines from which EVs were isolated. Each cell line is seen as a function of its gene expression, that is, as a function of the genes whose number was skimmed in the previous step. **C**. Hierarchical clustering of gene expression. Cell lines from which EVs were isolated are represented on the columns, the genes on the lines. The data are normalized on line allowing the comparison of the expression of each gene in the various EVs

EVs have been recognized as pivotal mediators of intercellular signalling, fusing with recipient cells and transferring their bioactive molecules, thus influencing several bioactive processes such as cellular communication, immune response, blood coagulation, and tissue repair [29]. It has been reported that GBM cells release EVs containing mRNAs, miRNAs and angiogenic proteins. EV content is taken up by normal host cells, such as brain microvascular endothelial cells and stimulates tubule formation [32]. To characterize EV RNA composition, we performed RNA Sequencing (RNA-Seq) of total RNA extracted from EVs isolated from both sham or 50 Gy irradiated HMVECs and GSC#61. To identify the most relevant transcripts delivered by normal or tumor cell-derived EVs, we first skimmed the number of genes present in the dataset (Fig. 3A). We considered the difference in gene expression between HMVEC-derived EVs and GSC-derived EVs, focusing on the differential log-fold change (logFC) between the two cancer cell lines, pre- and post-irradiation. The main body of the distribution is located in the center of the graph where most of the genes do not undergo or undergo a slight variation in expression (Fig. 3A). We chose a logFC threshold of 4 thus skimming the initial distribution of about 200 K (198,093) transcripts to 16,486. We sought to classify EVs' content using EVs derived from GSC#61 irradiated with a dose of 50 Gy, using HMVEC-derived EVs as a control. Unsupervised data analysis, performed by Principal Component Analysis (PCA), stresses that GSC-derived EVs and HMVEC-derived EVs have a different gene expression profile that is further distinguished after the radiation dose (Fig. 3B). To better understand the complex gene regulation hidden in PCA's endpoints, we used hierarchical clustering to highlight the differences in expressive patterns, inherent in cell lines from which EVs were isolated, before and after irradiation. Greater differences were observed from the comparison between HMVEC-derived EVs and GSC-derived EVs after radiation dosing (Fig. 3C and Additional file 2: Table S1).

**Fig. 4:**
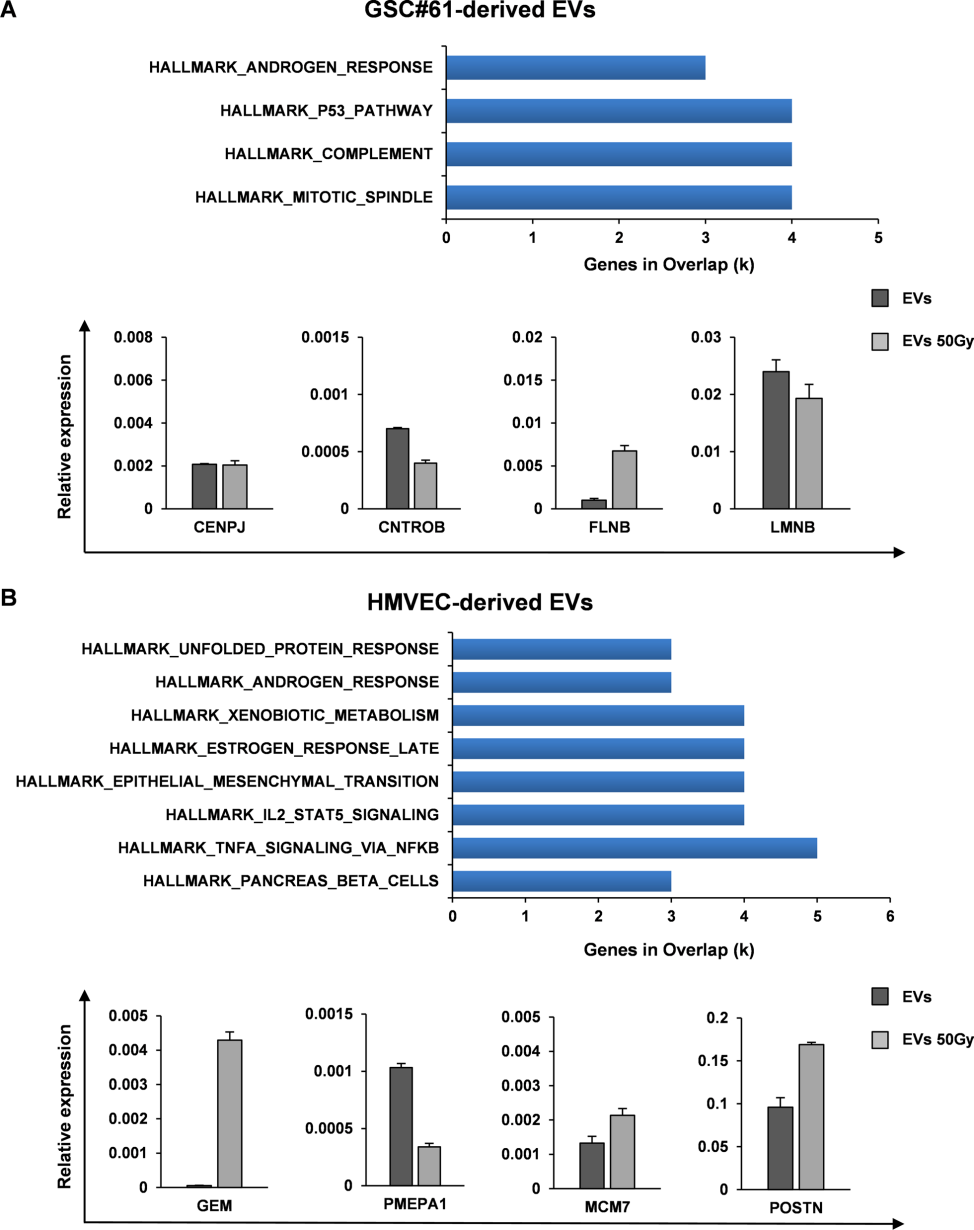
Filamin-B and Periostin transcripts are enriched in EVs isolated after irradiation. **A**. GSEA of highly modulated genes as assessed by RNA-Seq on EVs derived from irradiated GSC#61 (50 Gy) compared to EVs derived from sham-irradiated GSC#61 (*upper panel*). qRT-PCR analysis of CENPJ, CNTROB, FLNB and LMNB expression in GSC-derived EVs isolated from sham irradiated (EVs) and irradiated (EVs 50 Gy) GSC#61 (*lower panel*). Samples were run in duplicate. Data were normalized to the GAPDH expression in the corresponding samples. **B**. GSEA of highly modulated genes as assessed by RNA-Seq on EVs derived from irradiated HMVECs (50 Gy) compared to EVs derived from sham-irradiated HMVECs (*upper panel*). qRT-PCR analysis of GEM, MCM7, PMEPA1 and POSTN expression in HMVEC-derived EVs isolated from sham irradiated (EVs) and irradiated cells (EVs 50 Gy). Samples were run in duplicate. Data were normalized to the GAPDH expression in the corresponding samples

To detect significant variations in gene networks, we explored the Molecular Signature DataBase (MSigDB), a collection of annotated gene sets, with Gene Set Enrichment Analysis (GSEA) software. We searched among the transcripts that resulted upregulated, at least to two fold in the EVs derived from irradiated cells compared to EVs derived from sham irradiated cells, for enrichment in specific pathways. We therefore analyzed 116 unique identifiers corresponding to the upregulated transcripts in EVs derived from irradiated GSCs, with the functional annotation clustering tool. Twenty-one transcripts assigned to pathways enriched with a p-value below 0.001 were identified in GSC-derived EVs. The most significantly enriched pathways were related, among others, to mitotic spindle signaling pathway (Fig. 4A, *upper panel*). The four enriched transcripts associated to this signaling pathway were: centromere protein J (CENPJ), centrobin (CNTROB), filamin-B (FLNB) and lamin B1 (LMNB). To validate GSEA data, qRT-PCR analysis of CENPJ, CNTROB, FLNB and LMNB was performed in GSC-derived EVs isolated from GSC#61, GSC#1, GSC#83 and GSC#163 (Fig. 4A, *lower panel* and Additional file 3: Fig. S2). CENPJ and CNTROB expression was barely detectable in all the four GSC-derived EVs analyzed, LMNB transcript was enriched in only one out of the four irradiated GSC-derived EVs, whereas FLNB expression was increased ranging from 1.6 to 6.6 fold in the EVs isolated from all the four GSC lines after irradiation (Fig. 4A, *lower panel* and Additional file 3: Fig. S2).

By using the same approach, we analyzed 127 unique identifiers corresponding to the upregulated transcripts in EVs derived from irradiated HMVECs. Fourteen genes, assigned to pathways enriched with a p-value below 0.001, were identified. The most significantly enriched pathways were related, among others, to TNFα and epithelial to mesenchymal transition (EMT) signaling pathway in HMVEC-derived EVs (Fig. 4B, *upper panel*). The four enriched transcripts associated to EMT signaling pathway were: GTP binding protein overexpressed in skeletal muscle (GEM), Prostate transmembrane protein androgen induced 1 (PMEPA1), minichromosome maintenance protein 7 (MCM7) and periostin (POSTN). To validate GSEA data, qRT-PCR analysis of GEM, PMEPA1, MCM7 and POSTN was performed on EVs isolated from sham and 50 Gy irradiated HMVECs (Fig. 4B, *lower panel*). Except for PMEPA1, the expression of all the transcripts analyzed, was increased in HMVEC-derived EVs after irradiation, however, GEM and MCM7 expression was barely detectable (Fig. 4B, *lower panel*).

### FLNB is involved in radiation-induced senescence and migration ability of endothelial cells

The actin-binding protein FLNB has been identified in the secretome of senescent bone marrow and adipose mesenchymal stromal cells (MSCs) and it is involved in regulation of MSCs cellular senescence [33].

**Fig. 5:**
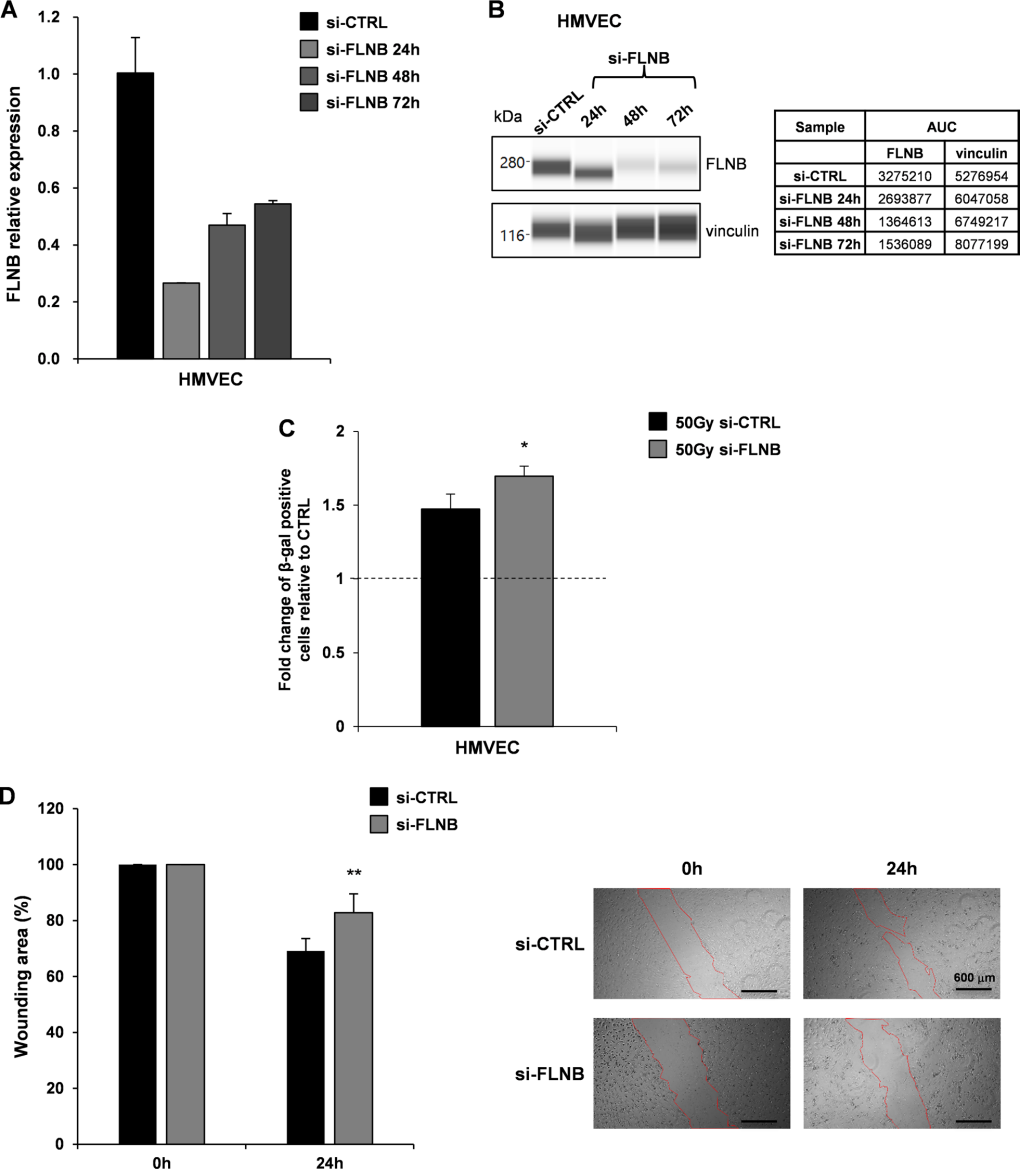
FLNB contributes to radiation-induced senescence and migration ability of HMVECs. **A**. qRT-PCR analysis of FLNB expression in HMVECs transiently transfected with si-CTRL or si-FLNB for 24 h, 48 h and 72 h. Samples were run in duplicate. Data were normalized to the GAPDH expression in the corresponding samples. **B**. WES full-length gel image of FLNB expression in HMVECs transiently transfected with si-CTRL or si-FLNB for 24 h, 48 h and 72 h (*left*). The relative amount of each immunoreactive band was shown. The protein expression was quantified using AUC (area under the curve) measurements generated using Compass Software (*right*). Signal intensity was normalized to the vinculin expression in the same sample. **C**. Measurement of β-gal positive irradiated HMVECs (50 Gy) relative to sham irradiated cells (CTRL, dashed line at value 1). HMVECs transiently transfected with si-CTRL (50 Gy si-CTRL) and HMVECs transiently transfected with si-FLNB (50 Gy si-FLNB) are shown. Results are shown as mean ± SD from two independent experiments. *p < 0.05 *vs* HMVECs si-CTRL (Student’s t test). **D**. Scratch-wound assay quantification (*left*) and images (*right*) of HMVECs transiently transfected with si-CTRL and HMVECs transiently transfected with si-FLNB at 0 h and 24 h time points. Values are reported as the percentage wound area and shown as mean ± SD from two independent experiments. **p < 0.01 *vs* HMVECs si-CTRL (Student’s t test) (scale bar: 600 µm; magnification 4X)

To verify the contribution of FLNB in the complex protein networks involved in the regulation of stress-induced, particularly radiation-induced, cellular senescence, we silenced the expression of FLNB in HMVECs. qRT-PCR analysis of FLNB expression in HMVECs transiently transfected with si-CTRL or si-FLNB for 24 h, 48 h and 72 h, revealed a significant reduction, more than 70%, of FLNB expression since 24 h post transfection (Fig. 5A, *left panel*). Accordingly, the relative amount of FLNB protein decreased over time as assessed by WES full-length gel image (Fig. 5A, *right panel*).

Measurement of β-gal positive 50 Gy irradiated HMVECs relative to sham irradiated cells (CTRL) after transfection with si-CTRL (50 Gy si-CTRL) or with si-FLNB (50 Gy si-FLNB) showed that FLNB silencing increased the percentage of β-gal positive cells after 50 Gy irradiation compared to CTRL cells (50 Gy si-CTRL), confirming that FLNB negatively regulate radiation-induced senescence in endothelial cells (Fig. 5C).

FLNB is mainly expressed in endothelial cells and plays a crucial role during vascular development. FLNB expression has been also confirmed in HUVECs and other endothelial cell types [34]. In the cultured endothelial cells analysed FLNB plays a key role in VEGF-stimulated angiogenesis, more specifically in the regulation of cell motility [34]. To verify the ability of FLNB to regulate also HMVEC motility we performed a scratch-wound assay with HMVECs transiently transfected with si-CTRL or with si-FLNB at 0 h and 24 h time points (Fig. 5D). The significant decreased motility of si-FLNB HMVECs, compared to si-CTRL transfected cells, confirmed a key role for FLNB in HMVEC motility, suggesting that FLNB could be one of the mediators that contributes to the effects of irradiated GSC-derived EVs on HMVEC senescence and motility.

### Periostin mediates the increase of GSC migration, clonogenic abilities and transdifferentiation potential induced by endothelial cell-derived EVs

Extracellular matrix protein POSTN emerged as one of the most interesting transcripts enriched in HMVEC-derived EVs after irradiation. POSTN has been shown to regulate multiple biological behaviors of tumor cells and to exert a pivotal role in remodeling various tumor microenvironments [35].

**Fig. 6:**
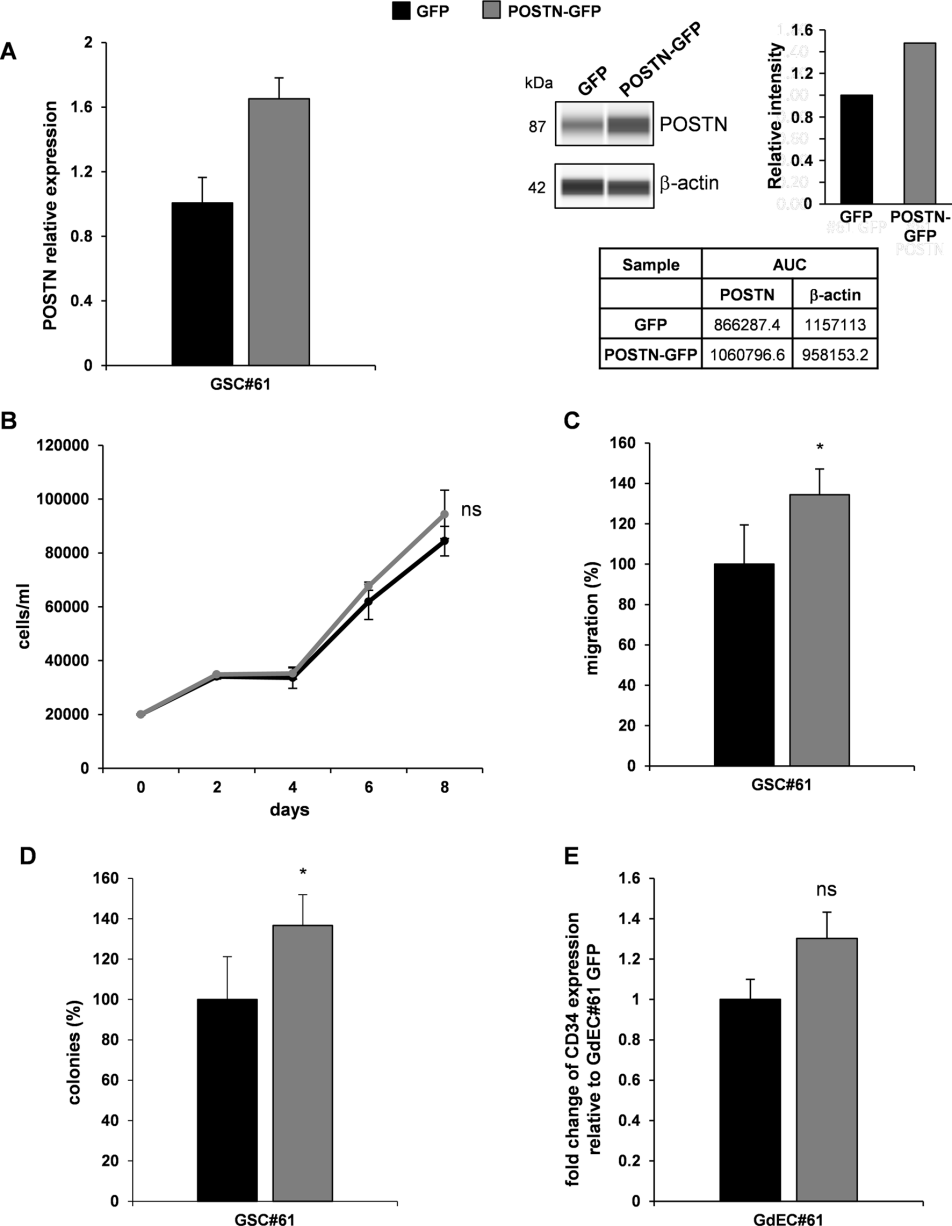
POSTN mediates the increase of GSC tumorigenic properties induced by HMVEC-derived EVs. **A**. qRT-PCR analysis of POSTN expression in GSC#61 transduced with GFP or POSTN-GFP vector (*left panel*). Samples were run in duplicate. Data were normalized to the GAPDH expression in the corresponding samples. WES full-length gel image of POSTN expression in GSC#61 transduced with GFP or POSTN-GFP vector (*right panel*). The protein expression was quantified using AUC (area under the curve) measurements generated using Compass Software. Signal intensity was normalized to the β-actin expression in the same sample. **B**–**D**. Functional in vitro assays on GSC#61 transduced with GFP or POSTN-GFP vector. Growth curve **B**: points and range lines at each day represent mean ± SD of at least two independent experiments in triplicate. Migration assay **C**: values are reported as percentage relative to GSC#61 transduced with GFP vector and shown as mean ± SD from two independent experiments in triplicate. Colony formation assay **D**: percent colony number values from two independent experiments in triplicate were calculated over GSC#61 transduced with GFP vector and are shown as mean ± SD. *p < 0.05 *vs* GFP vector (Student’s t test). **E**. Transdifferentiation assay: cytofluorimetric evaluation of CD34 expression. Values are reported as fold change relative to GdEC#61 transduced with GFP vector (ns, not significant)

Thus, we investigated whether the observed effect of HMVEC-derived EVs on GSC tumorigenic properties could be attributed, at least in part, to POSTN. To this end, we overexpressed POSTN in GSC#61 by transducing a lentiviral vector carrying POSTN and GFP as reporter gene, used to select cells by fluorescence activated cell sorting. To confirm transduction efficiency, POSTN expression was analyzed by qRT-PCR (Fig. 6A, *left panel*) and WES (Fig. 6A, *right panel*) in GSC#61 POSTN-GFP compared to GSC#61 GFP, used as control. Thus, to investigate if POSTN overexpression mimics the effect of the HMVEC-derived EVs on GSC tumorigenic properties, we performed in vitro functional assays, as shown in Figs. 6B–D. POSTN overexpression did not significantly affect the growth of transduced GSCs. Then, we investigated the effect of POSTN on the migration and colony formation abilities of GSC#61. We observed a significant increase in the motility and colony formation abilities of GSC#61 POSTN-GFP compared to GFP transduced cells. Moreover, we found that POSTN induced an increase of CD34 marker expression in transduced GSCs, under endothelial culture conditions, suggesting its involvement in promoting GSC endothelial transdifferentiation (Fig. 6E). To further confirm the contribution of POSTN to malignant features of GSCs, we transduced the line GSC#61 with three sh-POSTN-GFP lentiviral constructs (named sh-POSTN_A, sh-POSTN_B and sh-POSTN_C) or a sh-NTC-GFP lentiviral vector, used as control. POSTN silencing was verified by qRT-PCR (Additional file 4: Fig. S3A, *left panel*) and by WES (Additional file 4: Fig.S3A, *right panel*) in sh-POSTN (A, B, C) transduced GSC#61 compared to sh-NTC cells. For in vitro functional assays we selected the sh-POSTN_C vector since better reduced POSTN expression. We observed that POSTN silencing significantly impairs the growth, the migration and colony formation abilities of GSC#61 (Additional file 4: Fig. S3B-D). Moreover, we found that POSTN silencing induced a decrease of CD34 marker expression in sh-POSTN_C transduced GSCs, under endothelial culture conditions, compared to sh-NTC transduced GSC#61 (Additional file 4: Fig. S3E).

Altogether, our results suggest that POSTN could be one of the mediators that contributes to the effects of irradiated HMVEC-derived EVs on GSC tumorigenic abilities.

### Filamin-B and Periostin expression increases in recurrent GBM patients

In order to analyze the expression of FLNB and POSTN in GBM clinical samples, we interrogated the UCSC Xena public data resource (https://xena.ucsc.edu/) [36], particularly, we selected the GDC TCGA GBM dataset (n = 671 samples). The expression of both transcripts increased in GBM samples (n = 168) compared to solid normal tissues (n = 5) (Additional file 5: Fig. S4A-B). Noteworthy, recurrent GBM samples, derived from irradiation treated patients (n = 13), showed a further increase compared to primary GBM samples (n = 155), (Additional file 5: Fig. S4A-B), suggesting the relevance of these two genes in GBM pathogenesis.

**Fig. 7:**
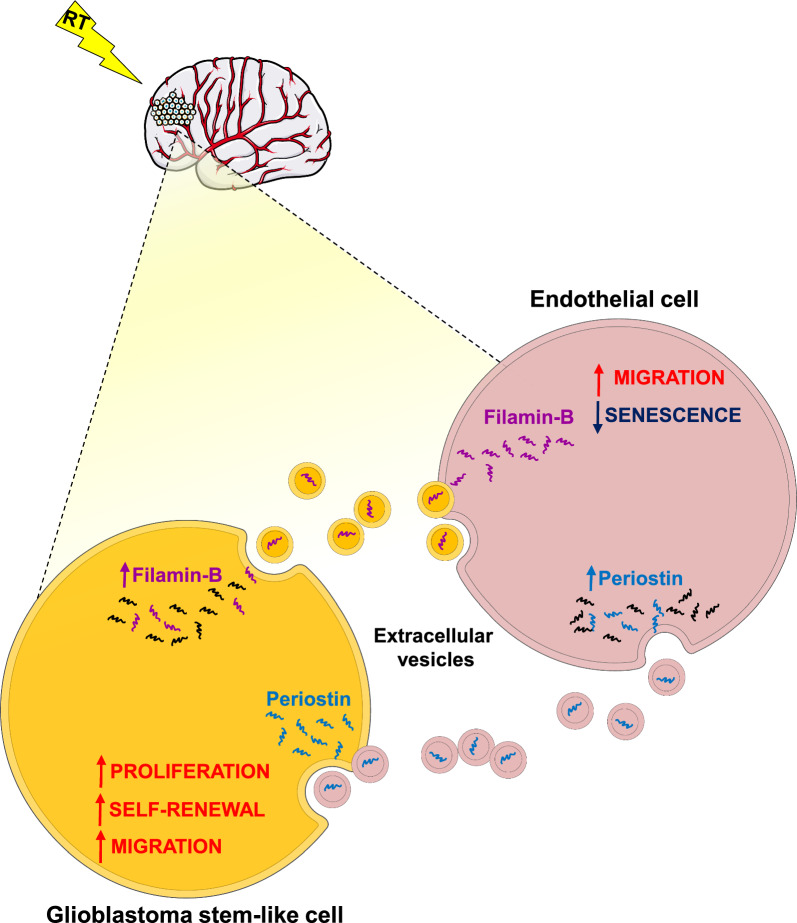
Schematic drawing of EV mediated GSC-endothelial cell communication. Radiation increased EV release and modified EV content in both GSCs and endothelial cells. Filamin-B mediates the effects of GSC-derived EVs on senescence-induced radiation and endothelial cell migration. Periostin mediates the effects of endothelial cell-derived EVs on GSC tumorigenic properties. Parts of the figure are drawn using pictures from Servier Medical Art (https://smart.servier.com (accessed on 22nd January 2024))

Altogether, our results indicated that radiation increased EV release and modified EV content in both GSCs and endothelial cells. FLNB mediates the effects of GSC-derived EVs on senescence-induced radiation and endothelial cell migration. Periostin mediates the effects of endothelial cell-derived EVs on GSC tumorigenic properties, as summarized in the schematic drawing (Fig. 7).

## Discussion

GBM is a highly aggressive and lethal tumor of the central nervous system, characterized by infiltrative growth, abundant angiogenesis and therapeutic resistance. A significant contribution to tumor growth is given by interaction of tumor cells with TME. TME is composed by various type of cells such as endothelial cells, extracellular matrix, and secreted factors that altogether, support survival and proliferation of tumor cells, including the stem-like cell compartment, considered to be major driver of GBM growth and progression. Moreover, it has been recently described the continuous crosstalk between GSCs and the TME, in developing and promoting radio- and chemo-resistance of GBM [37]. EVs are among the most interesting mediators of such critical crosstalk. EVs have, recently, drawn much attention as a new means of intercellular communication, due to their ability to carry various bioactive molecules into the surrounding milieu, that are responsible for altering cellular functions and/or reprogramming recipient cells into a dedifferentiated state [38, 39].

Several studies demonstrated that GSC-derived EVs can modulate the activity of MSCs and their miRNA profiles [40–42]. A further study by Pan et al. [43], demonstrated that GBM-derived EVs can promote proliferation, migration and intercellular communication in surrounding tumor cells by manipulating the PI3K-Akt-mTOR pathway. Accordingly, a more recent study, demonstrated that GSC-derived EVs have the potential to promote cell proliferation and to induce radiation resistance in tumor recipient cells [44].

It has been documented that various cellular insults, including ionizing radiation, cause tumor cells to increase the release of and alter the molecular composition of tumor exosomes [45, 46]. Specifically, Arscott et al. [47], provided evidences that radiation increases exosome release in a dose dependent manner and, radiation-derived exosomes from GBM cell lines enhance cell migration through a mechanism involving a rearrangement in exosome mRNA and protein composition.

In the present study, we confirmed that exposure to stress factors such as γ-ray exposure, increase EV release from both GSCs and HMVECs. Moreover, we demonstrated that incubation with GSC-derived EVs significantly reduced radiation-induced senescence of HMVECs and increased their motility whereas, incubation with HMVEC-derived EVs promoted tumorigenic properties and endothelial transdifferentiation of GSCs.

Analyzing EV RNA content, we found that GSC-derived EVs and HMVEC-derived EVs have a different gene expression profile that is further distinguished after the irradiation.

The most significantly enriched transcripts after γ-ray exposure in GSC-derived EVs were CENPJ, CNTROB, FLNB and LMNB. Among these, we selected FLNB as the only one to be enriched in all the four GSC lines tested, reproducibly. FLNB belongs to the actin-binding protein family, and is highly expressed in vessels and in endothelial cells in culture [48, 49]. Knock-down experiments show that FLNB deficiency in mice mainly causes skeletal malformations and vascular deficiencies [50]. Moreover, in the endothelial cell context at least two different functions for FLNB have been described maintaining cellular structure and facilitating cell migration following VEGF stimulation [34, 51]. In line with these data, we showed that silencing of FLNB significantly impairs migration ability of endothelial cells as assayed in a scratch-wound assay performed with HMVECs, suggesting its contribution in the effect induced by GSC-derived EVs on HMVEC migration.

Among the most significantly enriched transcripts after radiation exposure in HMVEC-derived EVs, we focused on POSTN, a matricellular protein that has been associated with glioma progression, particularly with glioma angiogenesis [52]. Immunohistochemical analysis and RNA in situ hybridization detected expression of POSTN by PDGFRβ+ pericytes but not by SOX2 + /OLIG2 + GSCs [53]. Silencing of POSTN in cultured pericytes results in a reduction of angiogenic capacity, confirming the pivotal role of this extracellular matrix protein in glioma angiogenesis [53].

Recently, it has been demonstrated that GSCs secrete POSTN to recruit tumor-associated macrophages (TAMs) whose density directly correlates with glioma grade, suggesting a supportive role for TAMs in tumor progression. TAM density correlates with POSTN levels in human GBMs. Silencing POSTN in GSCs markedly reduced TAM density, inhibited tumor growth, and increased survival of mice bearing GSC-derived xenografts, confirming the importance of POSTN for GSC niche [54]. Moreover, silencing of POSTN in xenografts specifically attenuated the tumor-supportive M2 type of TAMs that promote tumor progression and counteract the antitumor effects of T lymphocytes [54].

Taken together, our data showed that γ-ray exposure of GSCs and endothelial cells causes an increase in EV release and changes in RNA contents, both events leading to the creation of a microenvironment favorable to tumor progression. Particularly, we here dissected the EV functional role in the tumor context following radiation, and found that GSC-derived EVs reduced radiation-induced senescence and promoted cell migration of endothelial cells whereas, endothelial cell-derived EVs promote GSC self-renewal, proliferation, migration and endothelial transdifferentiation.

## Conclusions

In this study, we confirmed that EVs mediate cell–cell communication between neural and non-neural cells in the brain, as these processes can be co-opted to promote glioma cell growth. Radiation increased EV release and modified EV content in both GSCs and endothelial cells. Particularly, we found that FLNB could be a mediator of the effects of GSC-derived EVs on senescence-induced radiation and endothelial cell migration. On the other hand, we found that POSTN is an important mediator of the effects of endothelial cell-derived EVs on tumorigenic properties of GSCs promoting processes such as proliferation, self-renewal and migration, all of them essential hallmarks for glioma progression (Fig. 7).

### Supplementary Information

**Additional file 1: Figure S1.** Characterization of GSC-derived EVs. **A**. Size distribution of GSC-derived EVs analyzed by the Nanosight™ technology. **B**. WES full-length gel image of EV marker and β-actin expression in GSCs and GSC-derived EVs. Calnexin was used as a negative control. **C**. Measurement of EVs/cell released by irradiated GSCs (10 Gy and 50 Gy) relative to sham irradiated cells, used as control (0 Gy, dashed line at value 1). Four GSC lines (i.e. GSC#1, #61, #83 and #163) isolated from different GBM patients are shown. Results are reported as mean ± SD (n = 3). *p<0.05; **p<0.01; ***p<0.001 *vs* sham irradiated cells (Student’s t test). **D**. Size distribution of GSC-derived EVs (*left*, sham irradiated GSC-derived EVs; *right*, 50 Gy-irradiated GSC-derived EVs).

**Additional file 2: Table S1.** List of the most differentially expressed genes between EVs derived from sham (CTRL) and 50 Gy irradiated HMVECs and GSC#61.

**Additional file 3: Figure S2.** Validation by qRT-PCR of enriched transcripts in EVs isolated after irradiation from GSC lines.** A–C**. qRT-PCR analysis of CENPJ, CNTROB, FLNB and LMNB in GSC-derived EVs isolated from GSC#1 **A**, GSC#83 **B** and GSC#163 **C**. EVs were isolated from sham irradiated (EVs) and irradiated cells (EVs 50 Gy). Samples were run in duplicate. Data were normalized to the GAPDH expression in the corresponding samples.

**Additional file 4: Figure S3.** Effects of POSTN silencing on GSC tumorigenic properties and endothelial transdifferentiation potential. **A**. qRT-PCR analysis (left) and WES full-length gel image (center and right) of POSTN expression in sh-NTC or sh-POSTN GSC#61. For qRT-PCR, samples were run in duplicate and data were normalized to the GAPDH expression in the corresponding samples. For WES, the relative amount of each immunoreactive band was shown, the protein expression was quantified using AUC measurements and signal intensity was normalized to the β-actin expression in the same sample. **B**–**D**. Functional in vitro assays on sh-NTC or sh-POSTN_C GSC#61. Growth curve **B**: points and range lines at each day represent mean ± SD of at least two independent experiments in triplicate. Migration **C** and Colony formation **D** assays: values are reported as percentage relative to sh-NTC GSC#61 and shown as mean ± SD from two independent experiments in triplicate. **E**. Transdifferentiation assay: cytofluorimetric evaluation of CD34 expression. Values are reported as fold change relative to sh-NTC GdEC#61. ns, not significant; *p<0.05; ***p<0.001 vs sh-NTC vector. (Student’s t test).

**Additional file 5: Figure S4.** Filamin-B and Periostin expression increases in recurrent GBM patients. Box plot showing the expression of FLNB **A** and POSTN **B** in GBM clinical samples (https://xena.ucsc.edu/), GDC TCGA GBM dataset (n=671 samples). Significance (p values) were evaluated by One-way ANOVA.

## Data Availability

The datasets generated and analyzed during the current study are available from the corresponding author upon reasonable request.

## Abbreviations

GBM: Glioblastoma
EVs: Extracellular vesicles
POSTN: Periostin
FLNB: Filamin-B
GSEA: gene set enrichment analysis
C16-EVs: EVs secreted by Bodipy C_16_-labeled cells
