# Supplementary material for: Extracellular vesicles produced by irradiated endothelial or Glioblastoma stem cells promote tumor growth and vascularization modulating tumor microenvironment
Source: Cancer Cell Int. 2024 Feb 12;24:72. doi: 10.1186/s12935-024-03253-0 (PMC10863174; doi:10.1186/s12935-024-03253-0)
Supplement: Supplementary file 1 — Additional file 1: Figure S1. Characterization of GSC-derived EVs. A. Size distribution of GSC-derived EVs analyzed by the Nanosight™ technology. B. WES full-length gel image of EV marker and β-actin expression in GSCs and GSC-derived EVs. Calnexin was used as a negative control. C. Measurement of EVs/cell released by irradiated GSCs (10 Gy and 50 Gy) relative to sham irradiated cells, used as control (0 Gy, dashed line at value 1). Four GSC lines (i.e. GSC#1, #61, #83 and #163) isolated from different GBM patients are shown. Results are reported as mean ± SD (n = 3). *p<0.05; **p<0.01; ***p<0.001 vs sham irradiated cells (Student’s t test). D. Size distribution of GSC-derived EVs (left, sham irradiated GSC-derived EVs; right, 50 Gy-irradiated GSC-derived EVs). [file 12935_2024_3253_MOESM1_ESM.pdf]

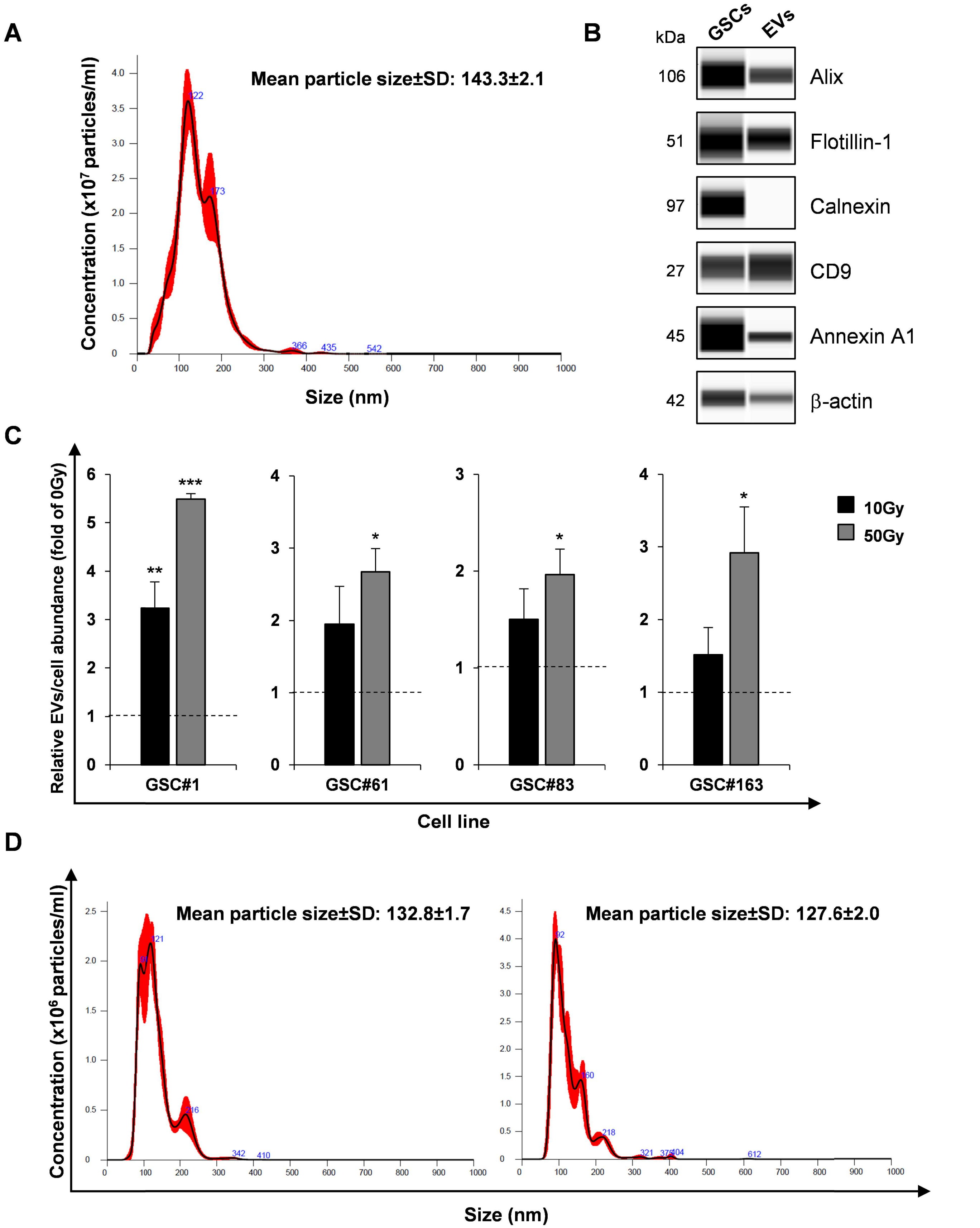

Additional file 1: Figure S1. Characterization of GSC-derived EVs. A. Size distribution of GSC-derived EVs analyzed by the Nanosight™ technology. **B.** WES full-length gel image of EV marker and β-actin expression in GSCs and GSC-derived EVs. Calnexin was used as a negative control. **C.** Measurement of EVs/cell released by irradiated GSCs (10Gy and 50Gy) relative to sham irradiated cells, used as control (0Gy, dashed line at value 1). Four GSC lines (i.e. GSC#1, #61, #83 and #163) isolated from different GBM patients are shown. Results are reported as mean ± SD (n = 3). *p<0.05; **p<0.01; ***p<0.001 *vs* sham irradiated cells (Student's t test). **D.** Size distribution of GSC-derived EVs (*left*, sham irradiated GSC-derived EVs; *right*, 50Gy-irradiated GSC-derived EVs).
