# Supplementary material for: Extracellular vesicles produced by irradiated endothelial or Glioblastoma stem cells promote tumor growth and vascularization modulating tumor microenvironment
Source: Cancer Cell Int. 2024 Feb 12;24:72. doi: 10.1186/s12935-024-03253-0 (PMC10863174; doi:10.1186/s12935-024-03253-0)
Supplement: Supplementary file 2 — Additional file 2: Table S1. List of the most differentially expressed genes between EVs derived from sham (CTRL) and 50 Gy irradiated HMVECs and GSC#61. [file 12935_2024_3253_MOESM2_ESM.pdf]

Additional file 2: Table S1. List of the most differentially expressed genes between EVs derived from sham (CTRL) and 50Gy irradiated HMVECs and GSC#61.

Index	HMVEC 50Gy	HMVEC CTRL	GSC#61 50Gy	GSC#61 CTRL
ERC1-201	0.00716426534947508	8.490528724283042	0.27361052763265054	7.486703154574102
PRKCQ-001	7.272570661258083	1.987314708787933e-06	6.947403015456089	1.2587463643172501e-08
PRUNE2-003	7.386705482400006	0.0	8.120686357927456	2.147618515877493
HPSE-001	0.005759829786671365	8.611253324642085	0.04889885111513312	7.0563097696888475
NAT8L-201	7.090734817064916	0.0	5.634263803585874	0.0
PRUNE2-202	0.0	6.8012639218861155	0.3986397749995843	7.976294190345706
FRMD8-001	7.45137649714054	0.0	6.370612431391971	0.0
RP1-163M9.8-001	0.23280608333014874	8.033937156112124	0.0	6.661268640098362
CACNA1G-201	0.0	7.767966425582013	0.0008255070853594048	6.211028205422675
SMAD9-201	8.281583529482699	0.0	5.418420444038093	0.0
RP11-514P8.6-001	0.0	6.8759244878744905	2.69440308151605e-05	6.3916301389758186
ENOX2-202	7.133631457035365	0.0	6.229545650322249	0.0
YES1-002	6.360385992647766	0.0	5.8054170403343335	0.0
CCDC25-006	0.0	6.846872167812703	0.0	5.844819561527153
SDK1-201	2.566875728413063e-06	7.944289673532482	0.0012097104571123201	6.315708695669743
PTK2B-001	0.0	6.763981703480691	0.0	5.097082858419428
SSNA1-002	6.682617329163772	0.0	4.744114415513926	0.0
ZNF746-201	0.0	6.461244125605837	4.560268789488391e-07	6.478213580576728
CCR9-001	5.569405775373003	0.0	5.60632279960651	0.0
ITGB1-001	8.43705098509625	0.0	5.660308782918495	0.48085851931720436
YTHDF3-001	7.257095951887291	6.003250331528408e-06	6.303870322259645	4.630334178934414e-07
TSC2-001	6.984954667962185	0.0	4.500505508078103	0.0
ZBTB32-201	0.0	6.946539220399246	0.0	5.046643740086108
NAT8L-001	0.0	7.328406726759203	0.0	5.431427341024915
PHRF1-007	8.094776332781379	0.007463086917690575	5.660100514331787	0.0
MYO5A-001	0.00398624851853277	8.322767038315785	1.8649944951947397	7.350995296122023
PUM1-002	6.375122123051542	0.0	4.588572037555544	0.0
PODN-202	6.0570605757907385	3.1147731504220435e-06	5.270720090686381	0.0
NEDD4-003	7.188379365612088	0.0	6.555825810681488	1.0076385712464795
PARL-001	6.504700175347249	0.0	4.4747990835182225	0.0
NET1-006	0.0	7.074570750742602	1.0967262863254799	6.355438647138827
NFE2L2-002	6.868616368240238	0.0	4.839868897729714	0.0
TRAPPC2-201	7.556749461838461	4.026221151079262e-07	5.495892336149304	0.0
TRPV1-001	7.159259377007027	0.0	5.726410094180927	7.000544864796571e-08
ORAI2-201	8.123320058403925	0.010759856644785102	4.57624085001455	0.04398768231055325
ZNF79-203	6.900367797797038	0.0	4.744353471730173	0.0
SETD1B-002	6.17766827952351	0.0024378617636990362	5.505837449217705	5.489590939484083e-05
PRSS51-004	6.106316856232874	0.0	4.709350569698517	0.0
DIAPH1-001	5.31359297656779	0.0	6.640806785767155	4.841184401223209e-06
RNF222-201	6.853000256049885	0.0	5.693955864135919	0.06776904137909025
MOAP1-002	6.837690844125462	0.0	4.431779363547582	0.0
PER3-201	7.635360971815536	0.43192265076439684	7.568352465650461	0.001554389191682476
FMN1-001	2.57472788950583e-08	7.521334928649804	6.295428733293874e-06	5.841211011628918
MFAP3L-001	6.011529528939208	0.0	4.508671538163766	0.0
TP53I11-006	0.0	7.461904257899801	0.0	4.8751765665197695
TGIF2-001	6.473944066476102	2.21578840314797e-06	6.518880493060595	0.0008295727964348726
IKZF2-202	6.876607165173057	0.0025515825040685588	5.277162337857443	0.0
NCOR2-005	9.274549977639294	1.6104406533356356e-06	4.900775342679658	0.11828521927229922
MKNK2-201	6.877739553167383	0.0	4.314135267459011	6.429488680427487e-05
DSCAML1-002	0.0	6.957428962927631	5.582533000480236e-08	5.290010657279307
TCAIM-007	0.0	5.44521344652258	0.0	5.527707513980628
MED29-001	7.186612748407628	6.922273807505268e-07	5.603677402940109	1.0670320531223e-08
CLCN6-001	0.0	7.410679917183326	2.1289181720228685	6.4774497805484375
RPL18-008	9.350509370562571	3.242758213460021e-05	4.323068618712336	6.183214498357858e-05
SEMA4D-005	6.278600779764271	0.0	4.904788954559693	0.0
CTNNBL1-202	6.296711475935099	0.0	6.09356159284642	0.0
RP5-1182A14.7-001	9.166898103476077	4.255490538346246	7.104376417194279	1.636901781664107

USP3-002	6.949230629784681	0.0	4.8897007108260615	1.4630866923863475e-08
GPRASP1-203	3.6853340972994446	0.0	7.025671332718202	0.02102350933496905
SKA3-001	5.735502871526936	0.0	5.5964286975703255	0.0
APOLD1-001	7.656214023380302	0.0	4.29966612271529	0.0
ZDHHC20-202	7.342579579094155	0.0	4.223961564040369	0.0
OSBPL2-001	1.808623638780837e-06	6.426477231518056	6.194988000008444e-06	6.542276452139844
ACAN-202	5.574824183584272e-07	6.9595785687771485	0.0034493414164796956	4.341846004760297
MYH10-001	7.534698840246595	0.016757437761159476	5.938103841838203	1.1515743283096542e-06
TSN-002	0.0	5.7618301354030415	0.0	4.870070444339325
OPTN-005	0.0	7.657618710711249	0.0	4.020298393050995
FAM219A-007	6.85403643296082	0.00023453641334998397	6.086191142835248	0.044333564667709324
RP11-514P8.8-001	4.667585639245063	4.252086691492402e-06	6.109118954333915	0.002880280657887618
PPT2-003	5.479928986644405	0.0	5.090970359075129	0.0
ZNF473-001	6.286632836887303	0.3240444960673677	4.434318775294575	0.0
PUM1-004	6.954293570035616	0.0	5.154914638733427	0.0
SPECC1L-001	0.23700947655575583	7.463679503465627	0.47533005196158384	6.377932591854598
SH3GLB1-201	6.341953866928518	0.0	5.36093592412537	1.4348029353143977e-08
TBX4-002	0.0	6.6300798268424925	0.0	4.297441120920207
NSRP1-007	5.596625153680423	0.0	4.808769559022969	0.0
VDAC2-201	6.881242380307628	0.0	4.126970169037098	0.0
MCM4-004	5.917703196330285	0.0	6.224213928792664	1.9633592517543028
ST3GAL6-004	6.948383394782159	0.03198000332248622	6.1842794322744385	0.0001627505595801826
PHF1-002	5.651517278213982	0.0	5.440535283370327	6.6116460021648506e-06
HNRNPAB-004	6.677605066239258	1.9585274745384464	5.1327337568786735	0.0
ATF6B-001	5.772884920131663	0.0	5.495931234357807	1.2240088157641307e-07
PHKA1-201	6.613420790089335	2.4478651024496438e-06	5.714612023747209	0.38618952863079004
BROX-203	7.742353235945435	0.0	4.792279229230215	0.0
WDR88-002	0.0	6.143424397753396	0.0	4.193136674664746
PI4KB-002	0.0	5.5301290842491095	0.0	5.212015497946824
ATAT1-003	2.168090119677504e-07	6.304967709928778	0.0	5.2944998275622135
UNKL-202	7.008916431963007	0.0	4.420736959369568	0.0
ACSM2B-001	0.0	5.080274554429225	0.0	5.866376704267761
GOLGA7B-001	6.767643452512618	1.507591578773177e-06	4.290729722860001	1.4439161188610724e-07
CFAP45-001	5.4311148650302705	0.0	5.149519384116616	0.0
CLIC4-002	0.0	5.396021270412476	0.0	5.129138848449237
LPAR2-002	5.556508130882301	0.0	5.073787603479388	0.0
DCAF17-201	0.0	6.522602366000053	0.466188133169231	5.9032298271999775
ARMC6-007	5.104920773316371	0.0	4.960621492808772	0.0
SNAP47-010	0.0	6.14664716885936	0.0	4.1624129898873745
CKAP2-004	5.831147223379274	0.0	4.5435029874762245	0.0003032844660866207
NFKBIE-004	0.0	6.653293364213862	0.0	4.090301948695809
GOLGA3-201	7.079379616661138	0.0	4.334672529965059	0.0
BIRC5-001	7.104734873324236	3.81003882860273e-08	4.0854327031699205	0.0
POLG-002	6.803394400390074	0.0	4.925436566091333	0.0
CDY2A-001	0.0	5.466493616816419	0.17733066347048063	5.633071980592987
TMBIM6-002	6.375202181588193	0.01569236616861584	4.922403641126606	0.0
CLDN6-201	0.0	5.906647804092356	0.0	4.099459821595815
GPR179-201	6.589959768355875	2.148390480850623	7.1648151773439155	0.07171997148279019
KCNIP1-201	5.385517678612314	0.0	4.861879384343357	0.0
PLOD2-002	6.117008093806775	0.0	4.198526666645807	0.0
C1orf141-203	0.0	5.019709893534204	0.0	5.151567470015652
RGS19-201	0.0	5.265238988194037	0.0	4.732123324636882
DGCR2-001	5.819349785828361	0.0	4.3336282044927215	1.8767222118551316e-06
NDFIP2-201	6.673859324198561	0.6894106944616558	5.497002467749326	0.12474076903074012
TBCD-007	0.0	5.208611240892826	0.0	4.187896941509868
NCAM1-201	1.0433894459305972	7.5067462341987925	2.4070146999530446	6.927467681291998
NLRC4-001	4.803418975631186	0.0	4.56222902727892	0.0
YWHAZ-004	4.897632787182369	0.0	4.733411366040087	0.0
NR2F1-AS1-004	6.167788603142812	4.294684292230583e-05	5.158715711760251	0.0

RGS19-001	5.96846998338965	0.0	4.032380695410547	0.0
RNF145-201	6.1847826930876675	1.7037002864940813	5.027023977695214	0.8125874902273742
EME1-002	0.0	6.500512420618574	0.0	4.468693404624005
ACTN2-004	0.0	5.6795654321059725	0.0	4.406400152090965
NDFIP2-001	0.0	6.324683443241109	0.002514699165341193	5.299731120908231
ZNF573-008	0.0	5.943497339949035	0.0	4.646380565812274
TMEM99-201	4.681442716642851	0.009101680164457124	5.70824691891941	0.005001833268850062
CDHR5-201	6.477532768669637	0.0	4.295251780708017	0.0
MYO5A-201	9.059318717125146	2.8520805474501936	7.58586837422211	1.3060885899539145
ZFP64-008	4.4820542606167875	0.0	5.848543935155036	0.0
HNRNPH3-001	5.791098870239179	0.0007707858899191355	4.230302273432168	0.07672146879140075
GLRB-001	4.882850226917492	0.0	4.259396368607699	0.0
KIAA1524-003	5.803939204106856	0.0	4.79584141223874	0.0
OGDHL-201	0.0	6.549811077518247	0.0	4.558218729752477
CD200-003	0.0	5.449713965561425	0.0	4.335943202741867
ALMS1-002	0.0029284468831050456	6.073701422789642	0.006671253092362943	7.23760081232054
ARMC6-014	0.0	5.091092997901494	0.0017182984605892831	5.178701297846119
EEF2KMT-001	5.808811616727845	0.0	5.090957483368813	0.0
MAMSTR-002	0.0	5.452341770089016	4.772852139539395e-06	4.228594393739479
NAPB-202	6.124754637589979	0.0	5.0302666871578054	0.0
ACTG1-201	5.979547676734209	0.0	5.464903860306112	0.6347095659230241
SEZ6L-006	5.368280930251169	0.0	5.071450750778043	0.0
GDAP1L1-202	6.623163373901328	0.0	4.697567178431956	2.100232777710171e-06
BTN2A1-001	0.0	7.059339945757965	0.0	4.376104553659261
FAM63B-004	0.0	6.259437875418042	2.926464557204258	7.562165206779418
RIC1-002	0.0	5.2423241123588085	0.0	4.624759082382837
ARHGEF10-002	0.010890225212374989	6.32479451259912	0.0	4.256031731807313
CSMD1-017	5.3434125633485525	2.781179824833694e-05	5.756224094061108	0.0031864734825815303
CSMD1-001	5.3434227933029925	2.7812583781284714e-05	5.756224094061108	0.0031864734825815303
GTF2I-001	5.884931904391685	0.0	5.159798843515011	0.0
SMIM7-019	5.395365090371193	0.0	4.717279825907596	0.0
IL12RB2-202	0.0	6.500399627176513	0.0	5.107699233467914
FDFT1-201	0.0	6.080651293165126	0.0	4.7546985078957995
ATP11A-202	3.913406479331286e-07	5.778178597780637	0.003079711992396537	5.3582472919404776
CDY18P-001	5.050151287121429	0.0	4.50007892498173	0.0
GUCD1-001	0.0	5.863921968096936	0.37188311674275465	5.893847604755038
NMT2-002	0.0	5.701182140578716	0.0	4.945722880414466
CYP3A7-001	5.890777964205478e-05	5.493690330260279	0.0	5.173627590842382
ZNF407-002	6.1289447543693445	0.00014474829319360505	5.317104486158805	9.538290806663381e-05
SCN5A-202	7.779652074901843e-08	6.064168624151726	0.0	5.438489998554408
PCED1B-AS1-011	6.0482851374979365	1.0795625494636216e-06	4.718103706211637	1.156003877501052e-06
CENPH-002	1.2209299715918378e-06	5.555049313177614	0.0017488925455089617	5.256017369088299
HELLS-001	5.594065081615629	4.293924951742406e-05	4.027093113872552	4.321082508513793e-07
TGFB2-001	7.172535272124914	0.00016838731944658034	5.373437128918338	0.0060847383438852
ERBIN-007	6.244164224691579	0.0	4.358896392598408	6.622919525249667e-06
ARPP21-009	4.1437271929515145	0.0	4.659254433877467	0.0
DDIAS-002	5.321534353914959	0.0	4.089995817843662	0.0
PRDM1-004	0.0	4.652228764861494	0.0	5.007645624191044
DYSF-002	0.030621288572125892	7.698909330953827	3.3806084298279318e-06	4.285505256033383
PPP4R1-002	6.02512353065391	0.0	4.047459501494085	0.0
SCARF2-002	6.412987452031303	0.159852193180172	4.259831665853806	0.0
ADNP-201	6.659244822813007	0.0	4.538941831411498	1.659147930912662e-06
CELA3A-004	0.0	4.631408724956518	0.0	4.622192425061048
CORO2B-203	0.0	5.837189346105137	2.4318511627540904e-05	4.775859377928624
WWOX-016	4.4858963004035495	0.0	4.84207991714044	0.0
PNLIPRP2-202	4.937525817196672	0.0	4.032396786490572	0.0
L3MBTL3-004	5.373985995333158	3.814335168705468e-05	4.045903431959329	0.0
RIC1-201	4.914174500705846	0.0	4.7680870153709	0.0
MCM3-001	0.0	7.194684829738504	0.07483627576932349	5.040531264122413

LARP4-015	5.413818436315298	0.0	4.281588708479718	0.0
RBMX-002	0.0	5.506689956921735	1.2204158460986792e-06	4.916199610508189
MEX3B-001	4.856641037358056	0.0	4.407906980152982	0.0
MADD-004	5.536669985624418	0.0	5.371029947915569	0.0
CANX-005	0.0	6.132261259687098	1.3309006480372283	5.603691728212346
SCFD2-002	0.0	4.8576933154149105	0.0	4.045517443357134
ATIC-007	4.456374473341087	0.0	4.113312583314517	0.0
ASZ1-003	5.120603271520189	0.0	5.166373487407944	0.0
FAM71D-001	4.562598239993536	0.0	5.539219901067517	0.22737345631907377
HPS1-011	2.6970219625570262e-06	6.023415565976094	0.0008351085923256999	5.288629595873649
OPA1-204	6.402285599295892	0.0	5.294845634229471	0.0
FAM83D-001	1.2097661264553949e-07	6.221969253838236	0.0	5.232819814778938
SPDYE11-001	4.635677944818995	0.0	4.881386977433044	0.0
SPDYE9P-001	4.6356528825942735	0.0	4.881386977433044	0.0
CNKSR2-202	0.0	4.670656771604776	0.002320102587419594	6.220371255311833
RPL23AP60-001	6.714568762452878	0.0	4.339705138906224	0.0885040367031559
ABCA8-201	5.301822103747277	0.0	5.090846747550626	0.0
ATF7IP2-006	0.0	6.29809922452006	0.02830903697250561	4.686332530117096
MGAT4A-001	0.6365559196673103	6.417921753081293	0.2561372006700316	6.70456486771392
TP73-001	0.012852602618618682	5.6724515857566224	0.0014950734985337273	5.076656831541633
OTUD5-004	6.000432890718603	2.249165026258093	5.416167385243821	0.08226523896720185
PMFBP1-004	0.0	5.2951233807385325	0.1659797834284379	6.038795395065609
POTEB2-001	4.992170098935148	0.0	4.472666038939681	0.0
EPB41L4A-201	0.0	4.487328093141477	0.03126946049724333	5.221583300955573
NKTR-018	0.2326487920050034	5.236348794435611	3.392987561497568e-08	5.05423648507868
LGI4-001	6.1400357081877805	0.0	4.111450282752804	0.0
ALDOA-030	0.0	4.758098059814601	0.0	4.047264399919002
ADRM1-202	6.518771739738183	0.0	4.166304282161593	3.330451776357738e-08
UNKL-002	0.0	4.934044742586341	0.0	4.200516507660519
PIK3CD-202	4.9846271024055	0.07203208580757399	4.250773378026266	2.501144921609432e-07
FILIP1L-001	4.1835181075416	0.0	4.218507768792692	0.0
MAP3K6-007	6.6919834563916005	0.004478807943863681	4.866180081561453	0.0029364806602485993
SCARF2-005	0.0	5.2825914151320905	0.08900628900771365	4.274567680413229
DLGAP4-202	0.0	6.311173944854964	0.0	4.345806519547803
NR2F1-AS1-011	0.0	4.85572319356858	0.005299025011597895	5.392959335443763
TAF6-003	6.004708447261571	0.005198257259704853	5.130169787111525	0.00484409761819281
MAN1C1-201	4.8128184428078615	0.0	4.486588327503667	0.0
RAB27A-201	7.189290322343299	0.0021080588285191976	5.0311339379947375	0.007975670472072764
NRAP-001	0.042748536028399696	5.398289247628244	0.011513272865693954	6.2120815589571805
MUC1-207	0.0	6.321294988172731	0.0	4.3307651308515
NEK6-002	5.509004874710117	0.0	4.007728784519175	7.813936952346365e-05
MTRR-001	5.554556430791947	0.0	4.174794541530629	0.0
FAM86C1-001	5.014417538714117	0.0	4.4709098366263165	0.4656915118301848
RER1-201	4.013537492299439	0.0	4.6313977621736235	0.0
HP-001	3.7163348646494927	0.0	4.235735039950586	0.0
PLEKHG4-001	0.0	6.239715076868258	5.1164143180769324e-05	5.187069854693639
ASNA1-201	0.0	5.282140623679547	0.0	4.708175659664436
CEP162-004	6.149656213771207	1.8270379792290208e-07	4.514481732570223	0.0
PITPNB-001	5.332692725026444	0.0	4.72979068298588	0.00019604068091313718
MERTK-201	0.0	5.3877205828650965	1.5094578204800753	5.6072153727667375
PBX3-002	0.0	5.131005075866544	0.0	5.121480185765679
TDRKH-004	4.487970827415427	2.0077035282166095e-07	6.110542297509276	0.011279651221772609
SLC39A12-002	0.0	4.908813946457136	0.006251370919052537	4.732403352143851
PCGF6-001	4.508270804507378	0.0	4.21488247425448	0.0
DDX47-001	4.705281355790394	0.0	4.6619507678272205	6.732484294011452e-08
KB-1552D7.2-201	4.987634635102128	0.0	5.7984762485410055	0.12214266553611518
COL11A1-201	0.0023752778074570976	5.229532290169423	0.0	4.298954429243481
CENPL-003	0.0	4.795332657804218	0.0	4.621344461969089
DDX54-001	6.212505412428348	6.774943118499472e-06	5.307664299155048	0.12074168211324153

GLB1L2-002	0.0	4.854574220611439	0.0	4.144068452017651
SNX16-201	0.0	4.942959592037837	4.388424143384359e-05	4.34100870339882
SLC9A3-001	5.0176714126632795	0.0	4.471214556511372	0.0001780779060645289
MCM10-001	5.874513303981829	1.9678814857638044e-07	4.06339062282153	0.0
TFRC-003	5.670275266702761	3.53326409912189e-08	5.022262342731219	0.0008982850125812983
VDR-001	0.0	6.164677458276377	0.0	4.654162942925421
KANSL1-001	3.5047941852693896	0.0	4.351391534315815	0.0
ABCA8-001	0.0004508360923929408	5.771971980588504	0.4446087680382836	4.647036439163253
MID1-005	4.268995594479633	0.0	4.500210791407508	0.0
PPP1R7-006	4.48273246517748	0.0	4.453161106670514	7.28148355492845e-05
OSBPL3-007	0.0	5.592471230657936	1.297565481241058	5.92431293867568
TCP10-201	2.0812842833053884e-07	5.196786979144921	1.4163767633656803e-05	4.565643254012046
ZNF16-001	5.367114059580855	0.0	4.747823509377573	0.18707246430673963
ACIN1-022	6.333156246532755	0.0	4.413873095966519	0.0003375795586158588
RUSC1-001	0.0	4.3758676980695235	0.0	5.34839765761838
PPP1R26-001	5.055631591264842	0.0	4.025509520244109	0.0
BPHL-004	4.964641186281054	2.4738811817672994	4.346481571589912	0.0
ADCY10-001	0.0	5.604748703276233	0.0	4.527459668401454
KCNAB1-006	4.3358639473753176	0.0	4.075836234353161	0.0
TCAIM-001	7.080987987777435	4.181838668056448	5.8468293568811305	1.1571341744885257
RPL13AP17-001	0.0	3.8425816378397255	0.0	4.001883937005115
CAPN9-001	0.0	4.311414364222458	0.0	5.089190323288376
LGALS3BP-007	0.0	5.36958817046966	1.104879159506591e-08	4.37179247752005
MYADM-002	4.344762661312623	0.0	4.0293040036407595	0.0
WDR92-004	5.049204515728368	0.00013730542945815995	4.010372599569268	0.0
NBEA-201	5.661960730944992	0.5950279034649193	4.698145453337313	0.12971475575056196
USP45-012	3.2438686098568437	6.051808165302735	0.0	5.007989812835643
ATP13A2-002	0.0	5.427635546312554	0.0	4.36033037098861
SRPK1-002	0.0	4.459379329156733	0.5908582385795511	5.451349083271772
COL6A5-004	0.0	3.60833390734385	0.0	4.488080208080254
MAZ-004	4.239252531087207	0.0	4.292289419082696	0.0
SPG11-006	5.675506033356262	0.0	5.008820465350375	0.5846627277556832
MASTL-004	0.0	4.294900870966697	0.0	4.99578029277198
MAP4K4-206	4.870025311290118	0.0	4.282563146790143	0.0
ZNF287-002	4.859262022759865	0.0	4.881997289562306	2.726100721053475e-05
TOP2B-002	0.0	3.9317047701835204	0.0	4.585631491498225
LTBP1-007	4.6932346841269075	0.0	4.603747223380024	0.0
EML3-015	0.0	4.412109574992121	0.0	4.774447213523953
EIF4G3-001	3.2326826404075817	5.457022831886458	0.0	6.5266091356272495
OR6K3-001	0.02596289867962525	4.1156169439306	0.0	4.826127677208471
SEMA6D-006	0.0	5.046761447331217	0.00020951310206491247	4.3669352842315
PARP3-001	0.0	3.5939699699861833	0.0	5.432810700873025
PIK3CD-201	6.647506172225079	4.208561939513456	6.639178732466934	0.005595722380952584
DDX3X-201	2.4483608228241533e-07	4.792170138032647	0.0	4.3975081567603524
SNX17-001	6.574206122988544	2.9812791020975227	4.229324632317463	0.0
TRAF5-001	4.062310330508072	0.0	4.369843282100019	2.947467658373368e-07
NKTR-013	4.5245043217004195	0.0	4.6943096323572835	0.0
NOMO1-006	4.145535345483315	1.8207149371450821	4.3704586790979505	0.0
EIF4ENIF1-003	3.552968474402101	0.0	4.037942867866088	0.0
CAPRIN2-003	0.0	5.1962871333968925	0.6332748502222092	4.988507609015845
NAB1-003	4.042308056050065	0.0	4.047140989068551	0.0
PARP11-001	3.132603985342751	0.0	6.020608971024261	0.0
NTRK2-003	0.4398230074791939	4.976257501126265	0.17103235625866595	4.898873520916633
GLB1-002	4.963403627056263	0.0	4.013207269207095	0.0
BIN1-006	5.1517872541416505	4.233914400564003e-06	4.627408458554701	8.76026379560763e-05
ZCCHC10-010	0.0	4.2093732379014215	3.175701173825199e-07	4.427893645361194
MYEOV-001	0.0	3.855982153979783	6.243768986133013e-06	4.991965992362115
CNTN6-004	0.0	4.580748556738169	7.73014889037168e-06	4.606941706920835
ZSCAN26-001	5.229155639511767	3.653351282096775	6.448962825392414	0.0017482233660596608

CTCFL-018	0.0	5.151527311318665	0.1834416164872644	4.486428999411433
ARHGAP12-202	2.406559931033064	5.364137402239118	0.32930407426521213	7.171859693645752
MAP2K7-003	4.447540645277396	0.007015377192713699	4.746908569933661	0.0
RAMP2-AS1-004	3.8983813413296895	0.0	4.983956045755473	0.0
SYTL2-202	4.915551334511664	0.0	4.409543308886762	0.4003457232768444
SARDH-001	3.6874875418660866	0.0	4.070935623682057	0.0
DCAF17-001	7.147388831665721	4.278115194141343	6.10663745642675	0.005902198282428658
WDR45B-001	4.143656699563137	0.0	5.3775661658043274	0.7861024990689859
SFTPA1-008	3.634906434859106	0.0	4.02893306894352	0.0
GCSH-002	3.2243853104812668	0.0	4.496650048312565	0.0
RUFY2-202	0.0	4.3627039537136305	1.8603644542084336e-07	4.418840762411659
THOC2-010	3.1163039146506346	0.0	4.611365144052714	0.0
RBM12-006	0.0	3.4925654499574055	0.0	4.443037522481935
MYCBP2-AS1-010	1.5542032897989493	4.093431358745584	0.0	4.271812057660477
FAM219A-004	4.066716492238124	6.472165742833534	0.0044359221148838526	6.383444622753224
CNKSR2-004	4.3626595766855125	1.386090305492298	6.440024195060647	0.8132953396248183
IL24-005	3.300600292741971	0.0	4.352032042066193	0.0
TNIP2-003	5.831245708681411	3.057796322729912	4.926851098529778	0.0
ATP8B4-201	4.370541934034369	0.0	4.628373995119435	0.001686945242706118
ADCK2-004	2.535890040367759	4.630246135971582	0.0	4.483169605151512
SHPRH-003	6.12153097022149	3.021791819323084	4.570585957302653	0.00012256360404328857
RSPO2-003	0.0	3.8472121249422475	0.0	4.257627045076255
ERBIN-201	8.226855090313729	3.433459982570534	4.531359583909028	0.0
LINC00969-051	4.852046419212849	1.5800071826364446	4.89804923180591	0.025825400062198873
DOCK8-002	0.0	4.157036370656272	7.96373213967599e-06	4.148732793050007
WDR89-201	4.368452222620251	7.910863672943219	3.0997020343554778	7.879435255456801
FANCC-001	7.1962175175779866	4.747619080404523	6.822770330706029	7.365964769306164e-08
PDXK-018	4.200989688830244	0.0	4.104548486353746	0.0
ABI2-201	3.3212228227235174	5.85422284227872	0.0	4.409239522665462
BTRC-002	3.8077977361023247	6.613186403009402	0.0332356755351299	6.462252964131749
MDM4-207	1.1475717083214962	6.521137708488985	2.81843485142993	7.593901588332105
FAM104A-001	3.541803017965954	0.0	4.650894024106073	0.0
SLC38A8-002	2.63723443844939	3.9803912084034003	0.0	4.660156297873699
RAB41-002	1.4369516438972831	3.622001261259406	0.003046255899761406	5.254852995105933
CHD1L-002	0.00020111409399393288	4.57602469844327	0.18626759034899368	5.142069844136969
FDPS-011	0.7794677419551208	3.545799516127378	0.0	4.435401553102947
ZMYM2-201	7.48240094209681	4.359697107341798	5.5352376710160085	0.0
STX5-004	2.520756789412136	4.534800011199961	0.0	4.045649362961985
PPT2-204	2.8346883119772383e-07	3.8189420264337515	0.0	4.142332134438328
CACNA1E-003	6.2911268198339325	7.4609355610746775	2.9488823338030006	7.528353653204008
SMC6-006	5.504505934688492	3.7931757002366573	5.305726222931314	0.0
EPS15L1-006	5.281051875395038	3.468608098051841	4.685625363772047	0.0
HDAC11-201	2.2716625007922024	5.238784887264005	0.0	4.228715433510313
NF2-001	4.463197565399176	0.0040376389896031655	5.608215058036471	0.619128633212298
CAP1-001	7.212677526027592	4.679880302231687	6.1559050087365135	0.0
PARD3B-201	3.843628197856468	0.00012019431632693145	4.02070502523911	3.2669400035113356e-07
COLGALT1-008	2.982154909086661	5.449594133742262	0.0	4.329375146986166
MYO5A-007	5.957440257269091	3.426192451882371	4.278399139746948	0.0
SSBP3-201	3.205506774461246	0.0	4.522324844272006	0.0
SCARNA2-201	5.642166618937692	3.571677794088164	4.631376515120424	0.0
OPTN-004	6.455832297699652	4.245904620924284	5.5878501600643915	0.0
KIF13B-004	4.217024405973727	4.9921753088338955e-06	4.350010681514604	0.0
PQLC2-002	5.552115299943786	3.6785704438630042	4.871571715701777	0.0
GLYR1-005	6.2283164057862495	1.631470679330382	4.76589504092151	0.0072115116641536985
HMGN2-201	4.42618760253549	6.569238199859343	1.053105070652018e-07	5.83488458553804
PDE1C-007	3.1800197100667167	4.832632380375452	0.0	4.155151032536183
NCLN-005	1.4595088720218157	3.8751244936175713	0.0	4.0345480059374985
SNX16-002	3.770902560701103	0.5559818495442618	4.132109920502891	0.0
RFX5-008	0.0	3.4338404517072156	0.0	4.0526567153762

PRPF31-001	7.5510579446365895	4.490441770848311	5.417730728661825	0.0
DPF3-006	4.745729605053736	3.48196708308703	4.761436358358256	0.0
SCN5A-003	4.008978521554795	6.367659475458118	0.013573616277037083	5.667646992775394
LGI4-003	3.1811763778930904	5.864155745883974	0.19574274983371645	4.585544541745808
GLIDR-001	5.7783967488266414	3.92939676365014	4.948990162906243	0.0
FECH-003	3.271699646037585	4.1094013664036355	0.0	4.858502646211713
A4GALT-003	3.324400295644291	4.876900450558232	0.0	4.280670431773727
SCN8A-001	3.400407862653998	6.50150115479057	0.000999282192925668	5.393129982552808
TACC3-001	5.69884257398809	3.7972366207061614	4.6169736164269155	0.0
IFIT2-002	3.2745566741816736	4.3743408380512765	0.0	4.867644287203739
ZNF521-003	6.848828272277618	4.467722736810774	5.295572075589579	0.0
EOGT-002	7.285074375644921	3.9273002174837592	4.373273424361505	0.0
ASPN-002	4.965339339913345	3.3245902180519105	4.775851348655343	0.003944402480334183
GRM1-001	6.415441947083296	3.6138198181607057	7.88457717578714	3.1835527793513947
DARS-AS1-017	4.0805177981835845	6.969104725097868	0.0	4.6172635886153035
RPS11-002	5.414534349793171	3.7005030996406547	4.449582596969321	0.0
ANKRD26-001	5.109421487425339	3.8523137540714814	4.984787828087555	0.0
MKNK2-004	2.9112845085215757	4.881171714012824	0.0	4.350948773603115
PRB1-003	5.24707644890794	7.842518932132283	0.003702720607494054	6.1194996892162346
CLUH-201	3.949128967469369	5.9596177788480516	0.0	5.133081029528186
ZEB1-202	7.3353580211583775	4.784580187374947	5.339881365173818	0.0
KTN1-001	3.1757606845083672	5.581915421910554	1.5572154210255375e-07	4.872213622113712
HNRNPR-002	6.602575906154446	4.2848072450909696	4.7520866570501985	0.0
MAPKAP1-015	4.0130873878350215	6.704586355795935	0.00924950870262296	4.246924685312579
SLC7A2-202	6.464180576633686	4.436028771528407	4.968825701367434	0.0
PMFBP1-201	6.406960147167597	5.092344636619496	6.309004699922125	0.018781511898720706
RP11-383G6.4-001	3.8066599045260276	4.281996864048085	0.018225958774280484	5.505480501071908
CTNNBL1-001	3.069624285897886	3.7719543139414315	0.0008069793119943792	6.08755477158129
TSN-001	7.300780366991337	4.850577359350012	5.285645243670094	0.0
SELT-201	4.897584018198355	6.069559040768045	0.0032491175307735984	6.005019122820005
ZSWIM8-201	5.1639100275412515	7.4055709183142415	0.000913090180585828	5.641139101601385
CLCNKB-001	4.948624197402921	6.611992968769656	2.169508000882914e-07	5.66411591912599
RNF40-001	7.19204815074003	4.344026725143212	4.455299158578573	0.0
ANXA6-001	5.974093796073778	3.9352728452901227	4.1925005325739075	0.0
FAM138D-001	4.269889353710685	5.2871318362632795	0.0	5.224584549776134
PGRMC2-001	5.436524502041137	4.188827730333909	4.887822435483923	0.0
IGF1R-001	8.84552332194689	5.648372556788446	5.832134834141009	0.0004409118391597077
CCDC110-003	3.232200950822478	1.7067437468214497	4.135804228878778	0.0
GABRA2-005	6.412641344095425	5.723139155093909	6.455278101061434	1.8437137623603834
KIAA1715-001	6.6516121472548635	5.022572526425239	5.589595470203271	0.0
SEPT2-007	6.925058194720559	5.172713023756511	5.691792557162399	1.1281013480648828e-06
CTD-3137H5.5-001	3.802720265259352	5.149138445434083	0.0	4.1688313502130985
DIP2A-001	5.0687470304467395	6.799619502571609	0.0007826275858255678	5.527500902389431
APOL4-002	4.223208608461853	6.898897594019144	0.0	4.155308958261664
CYP1B1-AS1-026	5.6685900589770055	4.239874853158108	4.63016137137059	0.0
MRPL37-201	4.360699929158244	2.3019394041324546	4.108456433834203	5.859861568138596e-05
RAI2-002	3.194601245302994	4.179326087526346	0.0	4.069849268687857
NPIPA7-002	3.738788898646062	4.670317452185065	0.0	4.270396994073041
RAVER2-001	6.2699345178421355	5.158926813559812	5.955443288909783	0.00032313054410207457
MAP7D3-001	7.556859754812058	5.209559801948937	5.305111437647187	0.0
KIAA0922-002	4.6863388529226615	5.127129126690586	0.8503477745221915	5.590629755642001
FUT3-004	4.486878368044973	3.074889244410437	4.0476293460390576	0.0
ACE2-201	5.999589239882576	5.560275152711952	6.593539995176377	1.1236378261480997
HSPH1-001	4.770433368835158	6.347430081318227	1.652319459715011e-06	4.998633704850968
SMARCC2-001	7.406914782801228	5.248343673398458	5.256709089078348	0.0
TXNRD2-001	5.581131063851084	3.856582405038129	5.128125865999689	1.0764989347083909
KCNT2-002	5.131639819880371	5.931405687584155	0.0	5.996430129649254
RP11-812E19.10-001	4.440843738239169	5.006883520851903	0.017447577339149496	5.3289753990281525
PRR13-002	5.545580905304917	4.285398207300986	4.548353628818145	0.0

EBF1-001	6.378680435002514	5.274929027014046	5.8741362734746865	0.00033513389022475165
RB1CC1-002	7.5170981439071936	6.176708812111374	6.756777966587611	0.09839499600952312
F7-002	4.591767773233783	5.213989950545052	7.908537394027252e-07	5.403308453935899
RRBP1-002	7.211346874634697	5.603699048214157	5.866123378548119	0.0
MRPL51P2-001	3.944348017627379	4.1156169439306	0.00010377523491933689	5.12825153909132
NRP1-202	6.950220121565206	4.837265548049064	4.708974773218391	0.0
PRDM1-001	6.791162489679277	5.52537839011434	5.9413653676178	1.680833627386626e-07
ARMCX2-011	4.22521971960631	2.3161899971034052	4.053820311845588	1.4668619812267475e-06
USP20-002	4.2222520695273	5.547130088422637	0.0	4.327509200660221
RPL38-008	8.46444797811311	5.074947406329998	4.581727063713532	0.5520512926960521
GP6-002	4.115922220011975	4.769241026304323	0.0	4.678211780698053
OPA1-201	5.436514911409817	3.677238539959707	4.344478335945695	0.001859666904891866
GRK6-010	4.67362787773004	5.8867381005388655	0.0	4.9022224782031145
ZBTB4-001	7.121240020311422	5.285132050625188	5.267877729623546	0.0
ICAM4-003	4.6645099665493115	6.257844204302694	3.418251837368371e-06	4.667129130446113
NEB-018	4.107585979197053	4.937765960147604	0.008276329493710292	5.6759018328237065
HMGN2P46-005	6.46783115675845	5.074224750000803	5.244830808873645	0.0
APP-001	6.929007232564982	4.820367766816871	4.618743013108177	4.499329329340612e-06
RP11-56L13.7-001	3.1172112636897937	3.3329583838285846	0.00010260713257111305	4.2021793006698
PRKAR1B-201	2.695097556540277	3.726485742143275	0.1070238168621683	4.694039672568631
SPOCK1-001	6.475807199148929	4.651166210448877	4.506746325018388	0.0
FERMT2-003	7.274047323588385	4.979116255249072	4.688439703363768	0.0
LRSAM1-006	3.177150380059372	3.951329208869944	0.19205127147030432	4.823587954756246
ARHGAP5-002	6.717142966689401	5.503212888827954	5.779208684690979	7.949538197097177e-07
CPNE3-001	5.081609397080057	3.9590105597222496	4.072172773498606	0.0
USP9YP5-001	4.1916781515509545	4.1156169439306	5.156898842770331	0.21609163915827553
IMPDH1-005	7.273282332698413	5.687792887135457	5.73352406216718	0.007447788502412026
ZBTB21-002	5.534144597580955	4.245102566774588	4.243827492289523	0.0
TFPI-201	7.592943385858396	5.625243526763342	5.466111550975003	0.000595790681369069
FAM63B-002	7.493460500356184	6.946815174363153	7.698674579000636	1.1271702049540049
CCR2-001	6.456728648116167	5.072755009572041	5.026113849476578	0.34524707630800466
TICRR-003	6.115855691931882	7.784628325462471	9.028558693058639e-09	6.134939534472962
HDLBP-002	8.392175690447255	5.265824407385944	4.6635204394506715	0.0
ADCY7-001	5.5981607339982125	6.476541925363827	7.61001699624939e-05	6.06232197225279
SEZ6L-004	5.2418338565349964	6.150272036037676	0.1741487761493006	5.539911856419195
HPGD-201	4.091620485402081	3.2065201756040893	4.112424865178192	0.0
CNIH4-001	8.654593171498702	6.9946097638087625	6.990495420651026	0.15194058193983706
CAPRIN2-001	6.476276105342763	4.7724349923234834	4.52120154657253	0.014454824446711258
COLQ-002	5.042288692317932	4.302113121154811	4.551358770849436	0.0
RAD23A-007	4.825412820927662	6.285244065689387	0.010899822058567647	4.650322184468921
OTUD5-202	4.11479592016799	4.813130033223845	0.0	4.304068537333762
NKRF-002	5.0370040793039506	4.404191850517352	4.689487714474882	1.3710100437237177e-07
FASTKD1-003	4.101852444808801	3.839338199769328	4.369282830277549	0.0
TMPRSS6-002	5.618364929090666	4.70709200631817	4.799631194416234	0.00010610294785745982
SIRT1-003	3.7296976564353246	3.744385636462612	0.0	4.5512320758730676
KRIT1-005	3.9541487194946514	2.954596463861197	4.0718179191961195	0.0
RP11-473I1.9-001	6.475900525961941	5.547253286259623	5.6441676814022665	7.783012520957756e-07
CALCRL-001	7.910373211288711	5.847594539970176	5.336401218774558	0.00438053343714484
LINC00919-002	5.151652891581884	4.50969686925153	4.664988078652531	0.0031175395701853706
KRT18-201	6.556569746250546	4.706339950527335	4.200781186458626	0.0006948149596768803
STAT2-006	5.357162434305927	4.647472438668372	4.7546931312049745	0.0
IL1RL1-002	4.898085563322647	4.791725886168655	5.51341632524956	0.0
LYST-201	4.8400387276113825	7.367465020731462	0.0	4.097027290230906
XPO4-201	6.414911467615535	7.492787943511975	1.1624393156776762	6.251930740364828
SPTB-001	7.299159423454536	4.968450232046889	4.276933007665278	0.00012372717000788504
SPECC1L-201	7.702540822530709	6.36596199712177	6.171173220643445	0.0
MCF2L-002	5.820335067376356	6.568350015144544	0.0	5.985507949216691
KIZ-013	4.327292817659535	4.685864476421111	6.0481798064730965	0.01743793933189596
TTC12-201	4.279695026337849	4.69633549791966	0.0	4.498767243691717

PARD3B-203	4.8582815010344085	4.901867486985182	5.742520758355095	0.05286654411608375
HLCS-001	4.418002094526785	4.079308581447553	1.0712992793143523e-07	5.60056919467829
SELV-201	5.471697340686979	6.148545638518264	0.0	5.539338144209971
XIRP2-002	3.7799774822635506	3.309963427899296	0.22863616139941256	5.111868358237499
AMOTL1-002	7.065387516045926	7.907661288386318	0.016742866265564014	7.045617533771791
FEZF2-004	5.445535442583199	6.841548493512951	0.11661123959195553	4.951305185788268
RPL23-004	10.248447762834955	5.95070398346466	4.564779020284199	0.0
AC104389.28-004	5.792276017223505	4.928457755508914	4.653578117221392	0.5135139569840464
NSMF-201	5.211099122642941	4.176309548194198	4.297427163919119	0.0006251304047016796
FRG1HP-003	5.8750618551198315	4.763622464833035	4.375076382418917	0.0007219408170987047
GANAB-201	8.21969070196109	6.855429448200461	6.4072566309066	0.0
CCNY-201	6.641218073771364	6.779734101589705	7.652685229055371	1.5697232879654284e-05
ZBTB48-004	3.5171231853405454	3.729554601386613	4.350591575046702	0.0
STX16-004	6.130466229408136	7.136500112952197	1.8730194355550933e-06	5.770065943780345
ZCCHC17-203	4.839175644280185	5.617125315845837	0.0	4.570082754336354
GRK6-001	6.6793486758961995	5.241722156646895	4.606462750115847	0.0
BTBD9-002	7.910856524169901	8.073822990431369	3.202148823104304	8.074794203543846
RNASE9-003	6.767641546572236	6.236777186258354	6.20673362727572	0.09004667425880263
TMEM164-201	5.792706897748096	5.515240225491381	5.6091126075057876	1.9486036873293906e-07
FLII-019	4.63204774436022	4.837219747955761	0.0	4.734587067080121
NXF2-001	5.786425805545363	5.885671149792231	0.0537897016910263	6.038464002605382
CCDC65-001	3.706095269688898	3.9220653489491433	4.424837205281384	9.410257850199086e-09
APLP1-007	3.734576536949066	3.433605396634245	0.0	4.351469106765323
FTH1-004	11.285456629541574	8.537006372472675	7.095155915147936	0.0
FAM223B-201	3.868523792077554	4.010335941031858	4.359909769851365	0.0
FAM223A-201	3.8685337444712173	4.010703116158599	4.359909769851365	0.0
NR3C2-003	4.665238577383186	5.0230235784545405	5.679670806170313	0.0003177282343933515
FNIP1-201	5.698738620481539	6.688189984782346	0.0	5.109440900745632
CASP2-201	6.434957812362029	7.4269541387944225	1.4279393774978324	5.845874171236743
EFCAB6-010	3.388146354755427	3.672596944838968	4.3638368240821	0.0
AP3M2-201	6.480775447947832	5.562349117070706	5.003161070457205	0.0
CUL1-003	4.989440072275436	5.18196102458746	0.0	4.965223837823495
RP5-881P19.7-201	6.6692205641570474	6.752800311242092	7.021266066431541	0.0041960308435198225
MSLNL-201	4.913154797081762	5.851232307943722	0.0	4.317806327086428
ZHX1-001	6.760076121872373	5.937643621566738	5.406323255212348	0.014574069862790914
COPRS-003	4.671339534163117	5.425169526678305	0.0	4.159840675991441
SERF1A-002	6.545609643861034	7.310354854418075	0.012068846004096015	6.008181554209418
LTBP1-001	5.776829499216417	5.878322771579572	0.18530460141894417	5.761179763753959
PRR29-006	5.577117216797691	6.358041581871906	0.0006274198617543381	4.996286588344599
AKAP1-003	5.544789269081084	6.7570431382669565	0.0	4.706427951275139
MPND-006	4.72731416651412	5.500415932938804	0.0	4.13977522695119
PEX10-002	6.2696976791451124	5.349190083075845	4.632696779024876	0.001594003148054839
PRAME-002	4.680171530954893	5.040274760226469	5.438411655139328	0.0
RP11-378A12.1-001	6.319093916563809	6.183872552456254	6.035604640239862	1.6034634206117304
ZIK1-001	6.976424155223351	6.322280292234287	5.705766986014965	7.811250902532869e-07
PTPN3-202	7.52700664688497	7.521733955427656	1.5608641728294013	7.4667785323063
CYP26B1-002	5.593639394424537	6.426306740004244	0.0022722139082436165	4.868492818841759
AMPD1-002	5.302548435411486	5.114501461568682	0.006661398554993537	5.4184926445481665
ZNF185-010	7.200629012716147	6.012416258060525	5.056289583841971	0.0
MAP2-014	5.63041757272001	6.014945193503234	1.1263732218955131	5.237728399963147
AKR1E2-004	5.441426551321605	5.043581295461562	4.621316379310816	0.2941814766348555
AC005301.9-001	6.851736801723126	7.806081090915386	0.0	5.978862630592282
B3GAT2-201	5.218859742504867	4.9514642997768235	4.6163368176744335	3.3466265489508756e-07
FBXL19-201	5.086672294106268	4.750763965805891	4.355697664595417	0.0
TRMT61A-003	4.837012041358367	5.482957402567264	0.0	4.201375281036924
SLC38A10-001	6.150337602709263	4.969043933244095	4.026931602299526	0.0
ESCO1-002	7.215796241716463	6.128665645669742	5.22814804898353	0.7616139100103443
PLPPR2-001	5.215644262967426	5.977418980707587	0.0	4.48037054660941
SPG20-202	7.463087515128461	6.432750077655051	5.46361759134805	0.0

FKBP4P7-001	5.677288704857261	5.240323543009883	4.701846329876423	0.12448350057304043
VEPH1-004	3.3377166184640963	3.5097087980501347	4.770912323940417	1.6086901136582553e-08
CFAP61-202	3.6969086830601365	4.088446684690345	4.373713791071899	0.0645597290327156
MAP3K3-002	6.335955937774307	5.269338731968702	4.303658180974775	0.0
SRP72-005	5.996746400951722	5.38731398926192	4.68351953911827	0.0
LYPLA1-202	7.272100497012957	6.041695918126248	4.913441171940619	2.501190512491885e-08
GOLGA4-006	8.845216306674804	6.3052226977479116	4.578938825832055	0.0
SERF2-002	9.8426748116962	7.263876776605002	5.4072749178590644	0.004254822282562503
SLC23A1-002	5.759905730151864	6.786393860295326	0.7737419402160514	4.820497753824772
AC012358.8-011	4.246830755207233	3.791278072016728	0.0	4.520602887033866
IST1-008	5.987709669339984	6.033252544919535	5.752489350390685	0.0
SULT4A1-001	5.868010377033416	6.412746180716725	0.0	5.116983104394228
PLXNB2-001	8.162290416007306	5.822591999598277	4.197335890831277	3.578071033177473e-08
MGA-201	6.1972127985635375	5.809004203388322	5.160275065818068	0.0
GNG2-001	4.210555616904226	4.953603791796151	5.523382992292215	4.9326699705352574e-08
STAMBP-201	5.6594524777426445	5.869195225944118	5.7078372511040705	0.0
ZFAND2B-002	5.388128415291886	5.699258262438898	0.0	4.781900145566914
RBPJ-202	6.7411259592997865	5.9656304228143995	4.983562168171067	0.0001170970097308213
PRRC2B-004	5.269259081894728	5.98838214436302	0.0	4.360675032308486
ASPH-201	7.746604485795399	6.732133613888845	5.498298160635067	0.0
HOXA1-002	5.401209984545175	5.317851414396136	0.0	5.05401245267449
CBLB-003	5.387314928107974	6.662990018500249	0.0006543560374410128	4.139337474740273
CRAMP1-001	7.245373165451868	7.166567605870653	6.520659593528717	1.9302282306556982e-05
ATF6B-002	6.371113521253012	6.65390716894567	0.005076878198468141	5.599532462607958
STAU1-004	6.658393942119926	5.686644372536435	4.5265694231810585	0.0018502208736042964
WDR36-201	5.952700228331157	6.252547836939928	0.45526180484024914	5.227557764467396
TWF1-001	5.364374242301387	5.748940972984725	3.087012381960062e-08	4.560082293816584
HIVEP3-004	5.034554096363838	5.403445705499234	5.227745245150942	1.6414297402447408e-06
HEATR4-001	6.880204412568187	6.659818557533791	5.841524901983642	0.0
ALDH18A1-001	6.7400481379365	6.107198022946394	5.042627325437361	0.02158187748419403
RPS13-001	10.211795317320279	7.355371009567788	5.083475322044258	1.7068290959442374e-05
DNAH9-001	8.42158104329217	8.74982111849207	8.364691445013715	1.4381917006800042
MRS2-201	4.662280917343363	5.2415422130797324	5.25032914924401	0.0
CDH20-002	5.824745870473134	5.165252729109743	0.0	5.8600552124969685
PTPRQ-001	7.064509651140676	6.929682750724196	6.08163669420793	6.943950059625842e-08
GSTCD-006	6.82378546318361	5.7583175184242705	4.416674341134822	0.0
CBFA2T3-002	4.960591888585009	5.007282513957544	0.3450852289424115	4.50483705074808
EPHX1-202	6.2201749269058935	6.972555330586447	7.613497678923502e-06	4.957473754668406
NHS-003	1.176227074527073	0.03498308102946981	6.728279059493667	2.5421135082103277
CT45A9-001	1.8763297609288565	2.0952098263961902	6.0916839000882765	2.0766593244806137
GAPT-201	3.777509673798604	2.341741658036451	1.0604947452401588e-06	5.862408346619308
CFAP47-201	4.244740028458851	3.519514774682393	0.0	4.492792743686695
CRYL1-002	4.567187097687347	4.337785709025222	0.0	4.185629113314506
EYA3-201	8.764948217257597	8.688132270692305	0.00023899753294677624	7.7721269357616025
SEPHS1-201	6.277549117306805	6.250557965226826	2.3115334048901604e-07	5.490042511905842
DAGLA-001	7.162459360547324	7.469667437723306	0.0005999989078696381	6.012251440795498
ASAP2-201	7.598965169770663	7.450795127449325	6.365139510157602	0.0
MYLK-AS2-001	3.9751116737241876	4.305308500318162	4.013745281688967	0.0
GLYR1-001	5.74287026963721	7.001647897703835	0.0	4.181322901434399
SERF2-007	5.431639420913347	5.379101334382622	4.599300479258287	0.0
SPACA5-001	3.6456603842020687	4.7281698012306155	5.354454619616055	0.0
CUX1-005	9.019137072217813	7.807040174422622	6.23181297765489	1.3654666830752404
UGT1A4-002	5.567042229867079	5.507455745723116	4.672617164620105	0.004683033325392168
PCDH1-002	5.505091362240496	6.343701408286373	0.0	4.152810687704113
INTS7-004	7.004790801292015	5.832817819528328	0.016491602319440673	7.242362505381654
GLB1L2-201	6.182165372283299	5.490834073494469	4.217857019716903	0.0
ARHGAP44-011	5.959554184255737	6.566071888251434	6.266462787017508	0.43238331989132756
LGALS8-AS1-001	5.958308911792386	5.417684818104396	4.220514753265681	0.0
RNF38-002	3.1906785503779282	4.516806035604121	5.564946478628724	0.0

TBCE-001	5.064260180588158	5.1807559230753375	0.0	4.177259696534181
RPS6KL1-201	7.3903863914313	7.798150111635932	6.975104623041387	0.06956651961204648
PITPNB-201	5.493014690096785	5.223019982333499	0.0	4.843487641876535
PPP1R12B-021	8.488810389303474	8.550645864477273	7.516453323233093	1.1595051092473634
GNG4-201	7.414627809139425	8.013957943459161	0.0	5.797943311543463
HMGA1-002	6.884362357276518	6.351578881794105	4.934066687188489	0.0
SLC1A5-004	4.9052552822203035	5.335961042980787	4.820266274388401	0.0
GLUL-201	4.774619862943808	3.866323189141633	6.960369157311402e-08	4.896934138358073
FN1-008	8.926772414250259	7.257201385330322	5.147160959834001	0.27752016300666565
TOP2B-001	7.30826837212207	7.100944391117047	5.768295266218282	0.0
NCDN-002	6.913659196644845	5.765733733767797	4.084419014122704	0.0
NAT10-002	6.514523549930747	6.237572082265573	4.971108051980592	0.0
ETV7-201	5.370119852595527	5.08846332555267	0.0	4.668514664989322
BMP2K-202	6.385517498394674	6.496466591947953	5.470892651180993	6.498588422386252e-05
RP11-439A17.4-002	4.750080905149629	4.189232564233233	0.0002979136923879901	4.421422814719364
CACNA2D4-001	4.592568483313498	5.301650877637809	5.018706495083079	0.0
ARHGEF18-002	5.614004092985152	6.049165150616166	0.0	4.313503337129609
FRG2-001	6.824591843456072	6.368214292158618	4.923262990681327	0.0
SCML1-201	4.266807317236599	5.309219243722087	5.423846594194142	0.0
KRT80-001	7.733559295474219	8.172000082857522	0.0032519022571225373	6.064724182111973
FAM110A-001	5.696569882350002	5.292794021310828	4.048854818554525	0.0
TMC3-001	8.27002519412256	7.775228149936589	1.6020727704617075	7.609091071687831
C1orf43-001	7.010527953227719	5.841164040992087	4.062870812528377	0.0
EGFL6-003	3.4269878931574382	4.503441708895685	4.8475347665777715	0.0031759674098996247
SRPK2-008	4.645867177400435	3.3723830566952206	6.081516596110051e-07	5.256328501770802
LCOR-202	5.996281266822999	5.900287253050992	4.723987257773727	2.5663039063342057e-05
NPIPB5-001	6.547140507984321	7.597400019853111	1.1137042934251146e-05	4.643056758848787
SNAP47-003	6.297461506773652	6.233770673600922	4.995744652455221	0.0
PQBP1-006	5.264415430924806	5.610628777296938	4.9339059027331045	0.48263109802181003
KCNQ4-001	6.317631428556536	6.975510896528218	6.2240613039822295	0.19794572344252487
TMPRSS11A-002	4.399533649820888	4.984907785365321	4.478335351992196	0.0
DSPP-001	6.217453649477151	5.74508925466414	0.0	5.343776109743248
ARHGEF15-001	7.068743599900696	7.2413035571816735	0.3256144580925958	5.625615986581527
DPY30-007	5.243243613982986	5.1993263752824594	4.402763948235267e-06	4.190310378061917
ISLR-001	6.47259775217737	6.18967777435469	0.0	5.3711301952208625
DCAF8-001	5.420079154486229	3.830790008397769	0.0	6.134228961196888
P3H4-001	6.215219259821902	6.273712398368155	5.0099731103421545	0.0
CILP2-002	5.715343823438605	6.011365284493023	4.981888376410029	0.0011835392914198812
ARF4-004	4.682130471122708	5.071392802475668	4.283527913907882	0.0
JADE2-201	5.066108667983462	4.929300191950866	0.0	4.054307640023765
CASP10-201	8.395318519406647	8.932317444291925	0.4219144712408061	6.3904728931835075
MYO1A-001	6.356870898599422	6.230628871158032	4.8111002591634594e-08	5.06244971127361
SPINK5-001	6.136869652751985	6.0534255603086455	1.991558110642681e-06	4.852828279537557
CLTCL1-002	5.8712459920581646	6.144738796006853	0.0	4.372546378577571
RB1CC1-001	3.1001300212222778	2.0942105894517207	1.9491765205853349	6.982453262468828
MCFD2-001	9.241758362643049	7.899766633163625	5.340052552167754	1.1651629392992292e-07
COL18A1-002	9.181624920120198	7.738057886317586	5.158607599565063	0.0
DYSF-001	8.27895837257656	7.513073125536997	5.3426784837549235	3.0166340205986323e-07
MARCH7-001	7.1894326073876655	6.747399965100279	4.9326505938598215	0.0
TMEM205-005	5.234455453978815	5.118720463935142	0.0	4.080307722128395
AC008268.2-001	4.209500851765954	3.242096597847331	0.0	4.1419722806887185
BAG5-001	6.725813582985829	6.47405507836194	8.572384325755785e-07	5.340966049164901
SSX5-002	6.1828830363256415	6.912096587432057	5.921408117268534	0.058059273376615005
SCN5A-014	7.956834213368347	6.7364866797609055	4.425102787235685	0.00013152085949383956
CD59-005	4.761794133977862	5.461629334912198	4.745557974243281	0.0
NFE2L1-001	7.24645400804301	7.645128326276504	6.175393291987836	0.0
SEPT2-002	6.891850517654195	6.392149284206852	4.5506960793472055	0.0
ARHGAP19-002	4.711041225367683	3.927952382307137	0.0	4.235761782434017
STXBP4-007	6.327051714856303	5.7177010535264365	0.0	5.289411972822364

DNAH17-001	8.050336565395972	8.364686056482926	7.091093282327428	1.4806768259426115
WDR91-001	7.544064833076556	7.925574854533753	6.406191126970342	0.2548891461086623
CCNY-202	7.881074071832794	6.758182936985049	2.287715149952276	7.724862800159592
NLRP7-003	5.614364467241738	5.901474875967303	4.623689370168255	0.0
PLCB4-202	4.4189440958097395	5.055079099472917	4.278607173569769	0.0
UBE2J2-002	5.127065380154407	4.2732889985135785	0.0	4.550194132421723
PRELID1-005	4.941383472511146	4.393429786261717	0.0	4.1024590616838985
RP4-545C24.1-008	2.883243053454413	4.05641774302702	4.582445262379657	0.5641243382281331
USP54-201	5.846664803956363	5.598342003069881	1.127536349602826e-05	4.528474761325581
THRA-005	5.847819127218146	5.752879554914	4.20292734700665	0.0
RAD17-007	4.848014089801049	5.218659710852982	4.144247043120518	0.0
ZNF589-005	5.607169645642024	4.60457993673361	0.0	5.031043065476412
RNF39-002	4.703393475955982	5.421630586014678	4.5966838784121125	0.0
MRPL52-003	5.6625307399184175	5.8174982123061225	4.413308898423226	0.0
ATP2A2-001	6.743552477857975	6.275951103335489	4.3427967530135305	0.0
ATP1A2-001	8.166300786165063	7.579002936213127	1.4119522319649134	7.0344093015865035
RP5-905H7.7-001	4.9647941440720755	3.9560576576661637	0.0	4.560655510060858
CEP41-001	7.197901698790966	7.368097579981791	0.0	5.224832147298338
NUF2-001	3.8245942396998758	2.271100584914879	0.0	4.794390519547202
SYTL3-001	6.2336551744784625	6.490095419861185	0.0	4.419646824897921
AKT1-005	4.076966012278558	5.231443323672953	4.906311672410492	0.0
MINOS1-001	6.543756659580051	7.1640465229802865	5.7717178995090945	0.0
SCN4B-001	6.7466113247163895	6.897363031774606	5.192674089628163	0.0
RFX3-202	6.449494679796166	6.633322541901348	5.342323731906722	0.8793160351959465
APOBR-001	7.519873054753532	7.389831598424398	5.355640467779547	0.00011531443988155379
TBX22-001	3.86101436920987	4.647273917709274	4.04814406518108	0.0
SCRIB-002	8.1911765413289	7.703476718176934	5.35873024145802	0.0
RTN4-002	9.47954385905038	7.862818977705591	4.86279665581864	0.0
CHD3-006	4.138560311386468	2.9479065146622085	0.0	4.208541373310642
SEMA4A-004	4.999271355890567	5.733024079364864	4.749628811914854	0.0
ATG13-004	6.731562572617838	5.752709591357377	0.009261455962056771	5.687456625305876
TDRKH-001	6.172687959976146	4.904046770002246	0.13077456271556795	5.654878260946093
ZNF195-203	6.169033041182513	6.315490642481068	0.0	4.342960160263417
RTEL1-201	6.010634367181047	5.451707704228347	0.00017308036308531622	4.7232092288376055
SCAMP4-001	5.857898155766594	5.534971493194354	0.0	4.417613834801706
DPY19L3-001	6.398590656625595	6.505602074902405	2.243961188998536e-07	4.506111867514081
FAM184A-201	2.7102847586708996	4.378463334080589	5.031029251166611	0.0
SMAD5-001	7.721930378956127	6.216504069183827	3.2527226473385115e-06	6.832478087936831
PSTPIP2-002	6.280755228989882	6.372344925175776	0.0	4.3854895405781145
COPB1-001	6.5287025334720505	7.00864982948044	0.001216296259447045	4.322491268401106
TDRD9-201	6.071803373758555	5.0589019014817795	0.04701411518998409	5.161345435955526
OCRL-002	7.1846298524767	6.540108266103646	5.96076376405858e-06	5.596860474879446
JADE1-201	6.550901882755574	7.249813828929434	5.683687427975973	0.0
SUPT7L-001	5.965704663558018	5.96554242936747	4.184075511194384	0.0
MAGEA10-201	6.0860027325422115	5.010519233458989	1.0427020538962404	5.658971030652516
OR2T5-001	3.9826322049407166	5.822388407945931	5.960779772387253	0.0
EIF4G1-005	5.659904367415893	6.1360341087381975	4.651620513502756	0.0
DUSP8-001	6.80145199558171	6.692925525734288	0.07972126839096894	4.886627307390414
CITED2-201	4.3417649655106985	5.077159746592322	4.10895499255302	0.0
GRAMD3-004	4.772319174673352	5.66837440340753	4.672166269909566	2.3121355720941476e-07
CNOT2-201	6.35210719238565	5.664259848937806	0.0	4.95797614882393
CDC25A-002	6.335665413726201	6.0370059401102365	0.0	4.592566785426057
EIF4G1-008	7.048245797716412	6.433949895581997	0.15837466818450383	5.430945586838705
FZD10-201	5.441751489017835	5.966656616667988	4.502280290304485	6.285426284329514e-08
PHLDA1-201	7.409115733762709	6.7111215718500254	4.298966263930666	0.17050211270057725
SNHG5-021	5.773135569947801	5.525372704888586	5.9327089093506474e-05	4.139327173088656
UBE3B-002	6.797501450000309	6.343233839554199	4.081524012955778	0.0
CENPT-002	4.297958366134853	5.108411627258262	4.108654860494631	0.0
AC009237.4-001	3.4095634463980113	4.783591790330453	4.517332089259977	0.0

LRRC37A3-201	6.807271381967454	6.887466720178233	0.0005653736820293326	4.567531711152404
DUSP10-002	5.817476631529847	4.465247300490046	0.0	5.101203401558479
IKZF5-201	5.535246640231917	6.008699928764182	4.394903472835663	0.0
WSCD1-201	7.076642187006718	7.372043618447239	3.907954583775634e-05	4.590575217637651
BTBD3-002	5.816316190849368	5.176330998951237	0.0	4.371489929711258
RNF6-006	6.204533050122686	6.139199112736969	4.059911153272415	0.0
NT5C2-001	7.493771624172261	6.1538804634010065	1.1966835241862217e-06	6.096257804392774
PHRF1-001	7.975989479284783	7.0497470338403465	0.0	6.038049654954715
FLOT1-008	4.242635297371036	5.1128225075718365	4.045286913060842	0.0
GLI1-001	6.974747646589211	6.470294602637711	1.2552370596622666e-05	4.99564942344651
FAM131B-001	7.414019101225746	5.887715814578725	0.0	6.2131708481922345
CRY2-001	5.720240679075025	6.497574023387961	4.879620082949574	1.0033753534423934e-07
PSG9-002	5.706927956300303	4.838968279262206	0.0009506216592441284	4.42234597412874
NKAIN2-201	4.6542375994712035	5.9232131219785	5.511599406670166	1.0150593657879317
AKAP2-006	6.8967787125288	7.308362243272978	5.127385327449875	6.888909290790487e-06
TTLL1-002	5.999154242491863	4.768261278008945	0.0	4.9509207907019395
CHTF18-005	5.800705358073867	5.429896923002139	0.0009910640456664262	4.052875489464764
NBPF6-202	6.477681910190989	6.595594710487995	0.006444337047174392	4.176637432173494
SLC12A5-202	7.153407064602731	6.981381130716881	0.10258953304986843	4.881007253544887
RAB43-004	5.679000269552132	7.268433926469746	6.218143464760365	0.1536241730054488
TMC8-002	6.1928362512484565	4.676945419787755	0.0	5.336066694043835
SACS-003	5.931750295984348	5.642943352300202	0.0	4.011641182877687
DYSF-009	8.80055190156937	8.179001076053598	4.969741875811129	0.05915517347992954
BICD1-002	5.28094505264475	6.473633802899685	5.390834948498623	0.45818753935757034
DEDD-201	3.807210388830376	5.037463018353475	4.258103448968581	4.850505383027568e-08
TCHP-003	4.3298147762114985	5.309503194434021	4.151292922330948	0.0
VWC2L-001	4.779626160126685	6.349839893203455	5.399053390974363	0.0
FKBP3-201	5.39424461976351	4.585369742191578	0.0	4.0238070513699125
NAV1-006	6.630442573977572	7.592537825967035	5.573921699875262	0.0
AR-203	5.665819268181679	6.326818396780774	4.888892226412523	0.7638554578659043
PLS1-001	4.8437462831547835	3.5003610567046914	0.0	4.170354611554531
FZD3-201	7.416962863780868	7.321375805540583	4.566839475225793	0.006951646450085777
NLRP1-008	6.874934339917449	5.756686343305682	0.002851897441009332	5.185948203644776
SLC4A9-003	4.888084501718113	6.431075937549858	5.31867332863338	0.0
BUD13-002	4.441551147551903	5.622987143016858	4.42749898478643	0.0
ARID3B-201	4.447207489191103	6.669890841927062	6.2403477119927535	0.0004125502149127655
CLCN4-003	2.8765539481005304	4.474841071293932	4.257410664313054	0.0
BIN2-001	4.331373290999433	5.847735498256125	4.891363638333686	0.0
ANKDD1A-002	6.289622416457044	5.566582053246018	6.069147442264931e-06	4.405402480713635
ADAMTS14-002	6.774649797705984	7.429736079173927	5.074350386111095	1.0318494177252754e-06
DEF8-001	3.7347434582646613	5.391299912991491	4.772807776308767	0.0
SAP130-002	5.7955138361402385	6.796409839499684	4.937192678280573	0.011401140570724708
KLHDC8A-001	6.254820290623977	5.344087079604775	0.0	4.448934478099569
ITPR1-201	8.598275671690265	8.073123105847944	1.1594955104116567	6.261084452474663
VPS13B-001	4.75444989629347	6.906707519422421	6.097988920160598	0.0
TMEM41B-001	8.117431992715447	7.485356028818249	4.1468010025779805	0.0
PRPF31-201	6.680873619668002	5.13835419603242	0.0	5.263170680897413
DNAAF2-002	5.458647359767746	6.201634921056748	4.229820552232725	6.330753959493402e-05
CEMIP-003	6.141666138043201	6.994856145428672	4.7986287069577775	0.0
C22orf29-003	7.100653812227206	7.556395228818005	4.843376777613666	0.0025036914084153043
RERE-002	6.575471806370091	6.791248604163012	4.175042356991546	0.0
AUTS2-008	3.6666625836588373	1.7844534859979233	0.066663410224033	4.391708127817594
PRDM2-002	2.3302610102148096	4.119540474215622	4.202517438575428	0.0
PCED1B-201	4.470428895841963	5.6633972961844545	4.2327971652249765	0.0
ZMAT1-001	3.590806959415987	5.847244358175221	5.575866108338254	0.0
FTH1-006	4.126471434894526	6.598950897649491	6.194034539140637	0.0
VDAC2-001	6.784439544950819	5.843746976178432	0.0	4.6485572075193735
TMC8-003	5.201277181933971	6.327408705365036	4.523805954067263	7.599386012804702e-06
YAF2-201	8.712626072693832	8.012539044890849	3.306009953038448e-08	5.616819634831855

PRODH-005	6.732914250113488	7.023551143839266	4.2648121873414055	2.8239212836736673e-07
PRF1-201	6.2722309124936695	4.7831163582993925	0.0	4.791804087771907
ATP2A3-201	6.269258886553183	6.80361922170941	4.293064158277579	0.0
UBE2I-001	6.740516263786251	6.418580022850826	0.0	4.091846498710229
PRDM10-008	5.2608559449213494	3.525239171308018	0.0	4.494533333389672
CERS1-001	6.084941544083576	6.688646977635925	4.246946417066938	0.0
USP5-001	4.732252667787177	6.679694075511776	5.464977383535999	0.0
TCAF2-005	4.289774301471025	2.4144385417499667	0.0	4.261456187266123
PTPRU-001	7.169291217750773	5.709002280613491	0.0	5.1272338344532535
LRRC9-001	5.9292271878904605	3.4408912333320596	0.0	5.664676103876989
PIEZO2-007	7.893039964142182	6.735270619330309	0.7863764897258831	5.777632492345908
PIK3CD-203	6.647506172225079	4.208561939513456	1.1682823973352521e-07	5.839916883858851
DNAJC19-002	5.616046435957772	3.6680165902943043	0.0	4.74745820427145
PLEKHB1-002	4.926976411284144	6.150260975963317	4.268455494627947	0.0
IMPDH1-008	4.355068828317588	5.6209719985768665	4.012968088131489	0.0
CACNA1B-202	6.246849301881699	8.418436979346469	6.368075152444413	5.799865337708462e-05
REST-204	7.861937612295903	5.450582448710338	1.4997989630197123e-05	6.316426415276741
POLR3H-001	6.649625776738017	5.959449467614826	1.5682565738574572e-07	4.043795886430781
ACTB-002	8.802781068290015	6.595120807964979	2.3006151264378945e-07	6.524273878401477
EWSR1-002	6.91656553407532	6.343117730038543	0.0	4.067290449661706
MCM7-001	3.851677300205149	6.094834555582106	5.195033606861807	0.0
WDR92-005	4.4571121293520015	2.506241029481244	0.0	4.156419044746112
NAALADL1-002	4.897186837948635	6.1322388615688554	4.138492770799183	0.04899430807878778
PRKCSH-003	7.345570477148148	6.573566472941787	0.0	4.324843898940501
MAN2A2-001	6.445270763396859	7.229122355413641	4.257534564141927	0.0
SPINK5-008	5.118506552718301	6.689494569943024	4.616125681891391	0.0
ZNF142-002	7.239434628263145	5.5010116072496436	0.0	5.044632042254064
RASA4B-002	5.489787880911879	3.688024071704417	0.0	4.2445084270876645
ITSN1-003	5.930729326279629	4.459938646416459	4.37616168888398e-06	4.106706560165175
SLC9C1-002	3.2643182711310583	6.171417927735266	5.933563193678399	0.0
BPTF-009	6.961086642618392	5.761164023593344	0.0	4.389436143119064
KLHL3-004	6.8156635901178575	5.044240106950035	0.0	4.8193826303885166
PTBP1-001	6.7161201568785796	4.531859949393713	0.0	5.191075746619286
RNF14-201	7.882069160247212	7.2980216258696196	0.0	4.380896172343646
CD59-006	4.045178964796088	1.7154850107926607	0.24293844608978538	4.89408885303555
MAP3K6-008	5.166144278043688	6.749039233307172	4.4873085151372765	1.541248344175427e-08
AKT3-001	3.2369643693915364	5.3176150637585735	4.345424175244347	0.0
FAM184A-001	6.327757701906911	5.002699668859113	9.447560544193321e-08	4.010723608173307
ADAM9-005	6.468722430186774	4.104135206401521	0.0	5.110531660217637
STX17-002	6.895082018108334	5.846725751078869	0.0	4.055700163133191
POU2F1-002	6.569204428446595	4.009630572672691	3.4379684416517294e-08	5.334389376902993
DAB2-201	8.002882182893233	5.7247393636250585	0.7687898812737608	6.282593084001252
CACFD1-202	6.597483065672364	4.894746592375017	0.0	4.389592050668568
DDAH1-001	7.827985542224544	6.292046903077942	0.0	4.779615101094181
UPF2-002	7.576521989509109	6.360395504993818	0.0	4.3009083077554155
LARP1-003	7.1647465146522835	5.134468149197279	0.0	4.797050684980022
INPP5K-001	6.815208162856402	5.326353930631924	0.0	4.084797378696601
NHS-202	5.823895583667786	3.316362503151381	1.1328875096245958	5.8789418199424235
ADRM1-001	7.384097139914737	6.177179301030653	0.0	4.067220058590346
TIGAR-001	8.30927230620086	5.49251593568382	0.0	5.939990197338047
MUC4-004	0.0	1.50063686731887e-06	3.996335093068791	8.56061206989526
SF1-006	5.389545961043287	2.8578063197933004	0.0	4.4665436736603965
ZFP36L1-001	8.4045345466736	6.656097570254163	0.0	4.825586799636912
ZNF502-201	5.746712025776456	2.8788161927860916	0.0013208319800039174	4.963403688543068
SLC28A1-202	4.103387643921457	1.3063790840801093	0.0	4.65558979125169
ARID2-001	7.300516922895684	4.617992715270082	0.5680763489893439	5.713501180704898
AAED1-002	7.635360971815536	5.832105561639476	0.0	4.3552481161159164
CDPF1-004	2.8519847062769847	5.26361763513927	4.086419848044343	0.0
RPS2-013	9.094826819749192	7.247227407821216	0.0	5.002487074831256

FN1-009	9.83336286385843	8.449928541213394	0.002051589993421245	4.848990937965103
DNAJC2-001	7.503486337675153	5.3375873258252176	0.0	4.47934137085449
LZTS1-201	7.556650190952516	0.0	2.0417264304103853	6.760546964442608
FAM65A-006	8.004379147419362	0.0	0.0	4.138239935386173
SCN4A-001	0.0	7.372083135022057	4.876109675914778	0.5984275556646229
FAM138E-001	2.12727518340361	5.381333225568361	5.082115696603533	0.0
ARL8B-003	6.490550057507803	4.136381494868819	0.0	4.077213630676476
SH3GL1-001	7.983566166382365	5.392977203542941	0.0	4.793356503198239
MYO7A-005	0.019242531167581158	7.384511770098395	4.095658955714168	0.0016565908008613311
RPL36-006	8.248594781784961	0.0	0.0	4.660902822590336
ISLR-002	0.0	6.213493780234472	5.371748708565364	1.2350511112685634
AP4S1-202	0.04496792471623482	7.054622373744849	4.878104730454809	0.5074089472332398
ZNF473-002	3.391789464827157	0.0	0.0	5.976650314274352
IL17REL-201	0.0	7.02086991748714	4.055637071005722	0.0
NONO-004	7.074657725350443	0.0	0.0	4.130051654006817
MOBP-015	4.3394792611013715	6.922296662579562	4.128807538936699	4.824383018542747e-06
NIPAL2-002	0.0	6.898583789171749	4.034760225099832	0.0
JMJD1C-201	8.071658867607988	5.102892945282982e-07	1.0885891127149777e-06	4.7683118108670755
C19orf54-003	6.878982213408317	4.35036923385464	0.0	4.036352544002405
DIDO1-007	2.767764833426915	7.366222875894303	6.579209582762188	0.08044145820576808
ARHGEF26-002	7.553845725512108	5.166419806869957	0.0	4.0128562117131725
PTPN23-001	0.0	7.661568589352089	4.667269644694867	0.000977791879954078
PTPN23-003	7.646255436455419	0.016354001873100422	0.5597298356270968	5.641190027457826
DPP3-001	6.784188967345967	2.9721148445085257	0.0	5.2361012848643185
TACC1-014	0.0	7.358991527081368	4.581092846265388	0.0
TTN-002	5.533319754855661	0.00031113773449945406	2.7342196668423076	6.8521432291238655
MPP2-202	0.0	6.568865890845977	4.121156519807104	0.0
ARHGAP12-008	0.0	3.98737836499096	6.334754028708935	5.737285080516806e-06
UGDH-001	6.603332196805692	0.0	0.0	4.193677460413954
TRAF5-201	3.091260775624538	0.0	0.0	4.9232618754821695
EXOSC2-201	9.022778838175675	1.4718177583478752e-06	0.00013567082114511052	5.851264620804158
PEX5-006	0.0	6.693950188456842	4.306206958378668	4.4453851688339355e-06
ODF2-008	6.418001565911372	3.40176143036613	0.8021918247339269	5.051871462465862
MYO18B-006	0.0	7.188718491157218	4.672964704817166	0.0
ARHGAP19-004	1.4787001728559754e-07	6.18031679307715	4.01143965483298	0.0
TUBB-004	1.4609520727998653e-06	7.678948504830495	5.052792925077454	8.256357166706394e-07
FAM107A-002	6.681721463277706	0.0	0.00011562470655652691	4.376069583583741
PPP6R2-201	6.13957626530173	0.0	0.0	4.022226976470445
DHDDS-002	6.873341054612622	0.0	0.0	4.529296541967476
ACO2-002	4.215661434306715	7.142626589232156	4.02038595880121	0.007672280218107792
CNTNAP4-201	0.0	3.631681668286459	5.54859988820402	0.0002805603760350629
NCKIPSD-002	6.198468885384686	0.0	0.0	4.077622259068875
YWHAE-014	7.726085224529495	4.577856892482645	0.0	4.335187912192964
APOBEC3A-001	0.0	6.1063279250153775	4.039560914545637	0.0
PMCHL2-004	6.374286246789357	0.0	0.0	4.227567568093833
ASAH1-051	2.1961943628774363	6.190646031781755	5.04625493109085	0.0
TAP2-201	0.0	7.381468421756247	5.211724914593545	0.1241620586833958
ZMYND15-004	0.0	6.105217693451345	4.077407159221893	6.001084064037193e-05
MPDZ-010	0.0	7.437503136409677	7.122844016147383	1.3948489367568635
SBNO2-003	0.0	6.703179759032139	4.522912889439148	0.0
SNORA50C-201	0.0	5.518894065567912	8.768668847370005	4.562247010345148
RPL32P3-002	6.218457996928675	0.002709427875585064	0.0	4.219872218829155
ELOVL5-202	7.1807417509096645	0.0	0.0	4.942621095792166
PMEPA1-001	6.050890488708596	0.0	0.0	4.143488199646801
PIH1D3-201	4.324797531643899	0.0	0.0	6.298571476893327
ZNF615-010	6.830144202126024e-07	6.071912002665366	4.165120846032218	0.0
RPLP0-008	10.557561364248103	8.2625987042931	0.6131333368578291	4.81668606378652
UBE2V1-204	4.256391879557082	0.0	0.0	6.181642887130314
CTSD-015	0.0	6.432028494601182	4.461084812944716	0.0

PC-005	6.376192538491436	0.0	0.0	4.43079207897134
MXD1-201	7.83966390474458	0.0	0.0002023935383179215	5.509529035367183
TRPM4-005	0.0	6.436821162417303	4.503615749312306	0.0
TMPRSS2-201	0.0001357128992750519	6.195209599108008	4.3546770334329254	7.296396234139682e-05
RRP1-001	6.178608958631597	0.0	0.0004748916495990766	4.345413636090877
CNOT3-005	0.22557951608617682	3.4256637582246428	4.549579673318206	0.11060482294758948
CDK5RAP3-006	0.0	6.782089096353973	4.813970958668012	0.0023034337509564946
ARMC1-001	6.334165585045179	0.0	0.0	4.485319037770611
UPF1-002	7.782767094641821e-06	6.24876507538427	4.4260666432294204	0.000768351804834136
CASP3-003	6.7010217283006925	0.0	0.0	4.761437553537972
AMD1-005	1.0263028129949268	4.569874800233024	4.30475550649703	0.0
DAG1-202	7.001963064307096	0.0	0.0	5.005468563525847
PLPP5-005	6.365971240091214	0.0	0.0	4.54819747822491
GOLGA6L10-203	0.004566436895408097	5.880191163808698	4.190252256201493	7.065726454268284e-08
DLC1-006	0.0	5.964196718230804	4.267922568713967	0.0018802358319910265
DDOST-201	7.6889281426342135	0.0	0.0	5.547201905657776
TBC1D2B-001	7.365743268173688	0.0019267958949297664	0.0	5.3052052467462865
CHKB-CPT1B-003	0.0	6.402051073120255	4.6148438899041455	6.225638349026589e-05
CTD-3092A11.3-001	0.0	5.84873545189282	4.210750440310572	0.0
KCNQ3-001	0.0	6.80906810015176	4.939725780757518	0.0
ZFP36-001	6.378710383517951	0.0	0.0	4.627662696027605
ZNF512-001	0.0	5.6344131744555614	4.080471258149452	0.0
MYH6-001	4.741294423221198e-06	7.647023815049239	7.398867714355074	1.165854469512156
ORC4-202	5.823642599926951	0.0	0.0	4.233365346948685
PTPN13-201	0.011188143880338276	6.8127017090217485	4.963108198578083	0.0
BCLAF1-202	5.271669447009811	0.0	1.1326753764548056	5.540600400069345
ANKRD17-012	0.0	6.816131006392105	5.010732861841279	0.0
CBX3-003	0.022814927872887258	3.3814093212713514	4.5910759312906775	0.0
OPALIN-202	0.0	5.494985706093315	4.015162717252172	0.0
CNTROB-001	2.7749601760336273e-06	7.125346825886165	5.327150266788051	0.03053413634962763
YWHAZ-201	8.715158282493624	0.0	0.0	6.483569175447368
GABARAPL1-001	5.833254071206608	0.0	0.0	4.302247765477088
THNSL1-001	3.664749650976351	0.0	0.0	4.978845427209272
ZNF205-201	0.0	5.761530905826238	4.2581630771301695	7.416033233830747e-08
OAT-007	5.6607830929017116	0.0	0.0	4.186187678789199
FAM9A-201	9.680044874810967e-06	8.076454238415007	6.044474602364958	9.492857985046044e-08
VWA9-007	2.170213220842673	5.860442296818044	4.161187020403739	0.001126781187947841
CTD-3006G17.2-003	0.0	7.611875662221656	5.856569968508401	0.0738621935398512
PLEKHG5-204	0.0	5.942049977630143	4.430697232429449	0.0
MSRB3-002	0.0	6.549945225127599	4.917326808804065	0.0
SCML1-004	3.7614808114311797	0.0	0.0755107648389829	5.185554172561673
GCFC2-001	6.199059773137181	0.0	0.0024098118129137775	4.666103406564851
TRMT10B-202	6.755706865563476	0.0	0.0	5.109251576569959
PLEKHM2-002	0.021892534061316266	6.826317189785411	5.141913077595951	0.0
GPR161-203	4.136672554778458	7.717217195879782	4.116336866727934	0.0
MAPK9-002	5.460794420381138	0.05782262343040826	0.428307420513678	4.792541202107198
MPHOSPH9-030	0.787394602999844	6.645891576690347	6.214696889913058	1.4706668861124443
SLC44A4-001	3.5095812667557507	0.0	0.0	4.622940430810117
XYLB-002	0.0	6.77044819513471	5.208937027060868	0.0
KHK-002	0.0	6.594763247040131	5.0807184801563485	0.0
RGS7-002	0.0	5.826639355759518	4.481041812194019	0.0
PPFIA3-002	6.243298241635871	9.529322954308589e-07	1.0187844049707047e-05	4.81504570924059
RPL13-016	6.193744399007978	0.0	0.0	4.786525185197376
BCL2L13-204	6.49478642680541	0.0	0.0	5.036504534996123
PTK6-201	6.257463910780132	0.0004338479279790915	0.0065148655238839665	4.861403302929881
USE1-003	5.6396611740425655	0.0	0.0	4.377135789733559
ZMYND15-201	5.891799930914553	1.1506085662422049e-05	1.2537855242817806e-07	4.57851834268154
AP2B1-021	0.0	3.613535321096662	6.263666849340841	0.964235253476078
EIF2A-002	5.7955138361402385	0.0	0.0	4.52337635136128

ZFP36-002	0.0	5.70772023499695	4.463529447398482	0.0
RP5-1187M17.10-201	3.878494293065424	0.0	0.0	4.963074886433053
CCSER2-002	5.7922198059273375	0.0	7.538569196519482e-05	4.543857351095031
ASXL1-001	7.505442027432522	0.0006829770685905246	1.5776830436020675e-05	5.937708528636977
EFR3A-001	7.00060955272357	0.0	6.975561727114618e-08	5.539457198495322
NKIRAS1-007	3.252946196726456	0.0	0.0	4.154819841814073
TMX2-002	5.53274567295179	0.0	0.0	4.3730896443577585
F3-002	5.600901839391382	0.0	0.0018689746325504038	4.432718336057419
OSR2-201	5.615898230355194	0.0	1.1911234183217707e-05	4.442327931300638
EIF4G1-006	0.0	4.747628827478755	5.399298806455038	1.0980724925075862
CWH43-001	0.0	4.441594687840517	5.612964464602236	0.0
SEPT9-006	1.902256636159081e-05	5.2566208691790175	4.169522032539651	0.0
GEM-002	5.3378931688835145	0.0	0.0	4.2442067087801325
DIDO1-201	7.547465721170115	0.003514760825570038	0.37908974189582334	6.728772321281273
ZNF185-205	0.0	5.6623015756488195	4.522977788828522	0.0
FGFR3-202	6.0846692402411735	0.0	0.00030107705528923957	4.887682467277821
HNRNPH3-009	4.491446376955465	0.7121928835815176	0.0	4.191090770631946
ENY2-002	5.547140669102269	7.281635671971623e-07	0.0	4.4619710096473275
BAK1-201	0.0	6.771301815975878	5.470969827507849	3.5130437511809535e-08
LMNB1-001	0.0	5.63480847360683	4.536769274953308	0.0
RAB6B-201	5.165785641960085	0.0	2.4990555430930124e-06	4.152958665037231
HMGCS1-001	0.030599534436588482	5.454928978990497	4.490201296975611	0.07215934225241326
MICAL2-202	7.24753404149142	0.0	0.0	5.8839736583361555
FLNB-001	0.06958124203982222	7.613400851913462	6.569428422625476	0.2569833411805312
CNTROB-002	6.035861675061518	0.0	0.045030192025110924	4.984995056889988
YWHAB-001	5.109830494599458	0.0	0.0	6.292690034643721
SLC16A11-201	5.218067702839653	5.978465576803404e-08	0.0	4.227814654238586
POSTN-201	5.143239116842961	0.0	0.0	4.168886861475141
ZNF549-001	4.083558741620685	0.0	0.0	5.029623747531975
POU2F1-010	0.0	3.93313854476525	4.836115890420019	0.0
EDEM3-005	3.490298084637008	0.0	0.0	4.296111052322265
BSDC1-001	6.677527974387032	0.0	0.0012467431465762989	5.4735695954082155
DAGLA-002	0.0	6.415727393482266	5.259596428633666	0.00015245206685243618
MBNL3-005	0.0	4.205730858972951	5.14296666628538	1.3986856695459327e-07
PAX6-202	6.352435090002078	0.0	0.07415071646273634	5.373203426735123
GRIA4-201	3.656858431821433	1.056362714735269e-06	1.9624775296784187e-05	4.468627811373414
PRSS21-002	5.162942322489239	0.0	0.0	4.238274484242413
IAPP-002	6.46474262508429	0.0	0.0	5.338697903587677
SLC50A1-002	0.0	3.972342780584259	4.830673130478738	9.728372395634092e-06
PCED1B-AS1-009	0.0	6.585991561229013	5.761964228777542	0.16229589118084
DCAKD-202	6.057119835629703	0.0	1.5073021143871337e-08	5.010765709227187
TRIP12-004	0.0	3.5020769229630107	4.235283089980333	1.605985085526917e-07
HHLA3-005	5.041071853628062	0.0	2.697041193971747e-06	4.186003176614354
TADA2B-002	3.2078753695656634	0.0	0.26320284751619827	4.359185327660091
MTF2-201	4.942525253735682	0.0	0.0	4.118272151133036
HNRNPR-201	4.850586001721016	1.0425598738454755e-05	9.026539703612678e-08	4.042975637035732
BRPF3-010	0.0	4.368242545037282	5.998114361292807	1.706239944182569
MCM7-201	4.625332098404906	0.0	0.0013564062460209299	5.527109243031586
PLPPR2-002	0.0	5.243319901277983	4.387311494386381	0.0
DCTN1-012	5.005523485952316	0.0	0.0	4.1908522240710715
NPNT-002	3.427273629354895	0.0	0.9060068584847137	5.585190929860356
AMBN-002	0.0	5.289651106870892	4.4547709461066	0.0
USP16-001	0.0	5.016663626134892	4.22480203225871	0.00025214477721894533
ZMAT1-201	4.848230393397951	0.0	0.0	5.737126513753589
TMEM164-202	5.926795247130179	0.0	0.0	5.01732207118907
LCLAT1-001	0.0	6.72695928116569	5.722490884920591	0.0
GBA3-002	3.4742708907543713	8.130402538496099e-05	0.0	4.112485202650697
CD163L1-002	5.0128746803515325	0.041840227455722856	0.0	4.176833173285465
CARNS1-201	5.055300611354102	8.404263955942376e-08	0.0	4.287291483521789

ORC4-204	0.00011129741808951242	8.840178555486204	7.630834702617171	0.03281039750446969
CHKB-002	5.134084247984729	0.0	0.0	4.368992584612679
KIZ-201	4.327872017738051	0.0	0.0	5.085206414040296
MKL1-203	0.0	4.974384607533219	4.2336325033185815	0.0
FAT1-201	6.298480290145085	0.006881250872763112	1.5980097811702656e-05	7.331617228772103
TP63-001	0.0	5.803168253730153	4.969237631590811	0.0
ORMDL3-001	5.423760661671494	0.8893567552896366	1.2131667139646843e-07	4.718365996834063
RBM45-201	0.0	5.536726808776378	4.7424005656015	2.6759853804334548e-08
TMEM71-002	4.689843189686027	0.0	0.17473284214603088	4.334234242786711
PCDH18-006	3.8426963361966595	0.0	0.0	4.489705287985676
NPIPP1-005	0.0	3.4905284118607094	4.080969208384688	9.468075097611613e-07
PSMB6-201	0.0	5.4813698611426025	4.715770938949728	0.0
GUCY1A3-008	0.0	4.697062501758436	4.034460320436782	0.000616034673409717
AMPH-002	0.0	6.398790089936516	5.522310170150384	1.5989322943824397e-07
RP11-111K18.1-001	5.448363341802103	0.0	0.004933332981415855	4.703194499598946
FCHO1-201	5.675733558254313	0.019866771483232563	0.003247024603973267	4.8649679900779885
MFSD6-001	7.112559425577842	0.0	0.0	6.16576211642996
RP4-665J23.1-006	0.0	5.201149699055241	4.506954788727615	1.8263542678864763e-06
SAXO1-001	4.385979977005904	0.0	0.0	5.046077890115307
AKAP9-202	0.0	5.251777088248807	4.5671838325735905	0.0
TPRKB-008	0.0	5.289651106870892	4.601048274435309	0.0
RFC2-009	0.0	3.489052965223057	4.020623913342524	0.0
CAMK2D-002	3.6556889782523276	0.0	0.0	4.203076511227093
OR1L1-001	4.201000980212164	0.0	0.0	4.804904461940881
ATXN7L3-009	5.238295171862873	0.0	0.0	4.602655457630468
AMY1B-001	3.835045086709369	0.0	0.0	4.368856401132927
STAC3-002	3.740506762677428	0.0	0.0	4.2566824148168
TTC24-006	0.014083893688202623	5.853969340508013	5.138498540914018	0.00013964396839292515
VIT-201	0.0	5.979744430925534	5.278031198631159	0.0
ADAM33-001	5.528396766487732	0.0	0.0	4.879931784899783
SLC25A27-201	3.815166683940736	2.0550340110190406e-05	0.0003144903389254016	4.325224311057223
FCHO1-001	6.0451946119458834e-05	4.8559583035784835	4.2915142636229024	0.0
ORMDL3-003	0.0	5.137967549349307	4.55123149779483	0.0
DNAH8-002	5.5390620751748875e-05	7.353814286653793	6.542630460232964	0.0
ORC3-004	0.0	5.245461283872804	4.651400509660807	0.0
SYCP2-201	5.941738019501209	0.0	0.0018094038062543465	5.282595470398042
C9orf43-001	6.088177715774291	0.0	7.918332246397772e-07	5.416555787497129
MAG-003	6.383867221395594	0.0	3.491550521746704e-08	5.685228562653479
FAM219A-008	7.954072725799753e-06	7.090188313871641	6.3274938139164645	0.00035993885563270864
PPT2-004	0.0	5.917774540449116	5.368739824537289	0.04213334663742441
SIRT5-201	5.87417345155546	0.0	5.430746484668652e-06	5.255934230675989
PRSS21-001	0.0	4.949075824362877	4.42251025212275	0.0
MYO5A-202	0.00039491878359948496	5.674314100306056	7.025118882567914	1.3060885899539145
ERI2-012	0.0	4.819245928119461	4.312276557551912	0.0
CTNND2-007	4.535067828551357	0.0	0.0	5.05351781894227
CDY1-001	0.0	3.9707119938867237	4.425548038038869	1.8520585418797673e-05
USP5-002	5.392535090052201	1.268981592927208e-06	0.0	4.848187579404635
CDCA8-002	0.0	5.49532242947873	4.94566753069815	0.0
NPIPB4-203	0.0007971412932168172	5.095955244710426	4.590955756225783	0.003386436555299113
OCA2-002	0.9315906951147082	6.872337353419636	4.836761009891885	0.0
HDGF-201	5.798285470045931	0.0	5.11570545188261e-07	6.417176155001186
TRIM6-001	4.296997319743587	0.0	0.0036108855323218484	4.774088980264674
POLR2B-001	0.0	4.745268113091196	4.277648159441783	0.0
FASTKD1-001	4.941957854124149	0.0	0.0	4.471007756312307
LIMK2-003	0.0	5.008446291890329	4.542015599826626	0.0
USP25-201	5.387701887750449	0.0	0.0006142551317520039	5.925857596114247
DAPK1-201	0.0	5.079291249583513	4.62125932248254	0.0
UBE4A-002	6.001091244537871	6.340601938380963e-07	2.843296734592895e-06	6.578647369704945
SPAG16-001	0.0	3.747291908655693	4.122915669502557	0.0

STAG2-019	0.0	5.782737622730964	5.2715633012655045	0.0
TACC3-201	5.441799267801101	0.0	0.0	4.9593714192416565
C2orf42-002	0.0	4.666981707194372	4.250238490945	0.0007048101884632657
ACTN2-001	0.0	6.246328544813326	5.707912813343856	0.0
ATXN7-001	0.03758989586952783	6.2589558559300364	5.65806310397881	0.0
STOX1-001	0.33883131055927707	5.706567424734708	4.655700213827445	0.0
SHFM1-001	0.0	5.072731668282757	5.546225855437156	0.0
PLEC-008	0.0	5.08518105077121	5.558137247999697	0.0
TOLLIP-004	4.470503836623946	0.0	0.0	4.086636350784857
RP11-12A20.13-001	4.404101862300032	0.0	0.0	4.035162688003651
LDLRAD1-202	0.0	7.543582362181972	6.952861369755112	9.48401421013121e-05
METTL8-001	9.075360366913102	2.630243104717696	0.004376413438411327	6.125492665736446
CENPJ-001	0.0	5.74117337732605	6.209677570337223	0.0
CNOT3-201	4.341529387630686	5.680028914617736e-05	0.0898526590940373	4.873818144724901
HIVEP1-201	7.516417759523335	0.10106804375557496	0.15983275308064726	7.091071549797181
PAX6-201	7.309652826005835e-07	6.094654482651212	5.647459759421518	0.0
MYH6-201	7.651945621806891	0.0025326449175881232	0.09945127236326251	7.305041014835424
FPR1-003	9.946596950300796e-07	5.3374642848619285	4.955855782996664	0.0
RBM45-002	5.151652891581884	0.0	0.0	5.539999649383266
KLHL8-002	8.895393895296296e-05	5.477460937434609	5.103832281048486	0.0
AP2B1-201	5.616584082757019	0.0001278590350960879	0.0	6.020776390863773
TTC24-201	5.806100979033822	1.0731891411396978e-06	1.4193853562522856e-08	5.430200549695766
WNK2-202	0.0	4.480505866659472	4.190367637696419	0.0
ABCC8-001	4.451969064893689	4.659889601754653e-06	0.0026268941686174913	4.736423603900132
TMCO6-001	4.357633907257649	0.0	0.0	4.101343670651424
PRG4-002	0.0	6.472422318874621	6.831836172467365	0.0
WHSC1-203	1.854886097699457	7.413748325576722	5.073156817618396	5.356890884874572e-05
C4A-013	4.194970109381077	0.0	0.00011409328498452942	4.415845904317008
C2CD2L-009	0.0	4.251653639157986	4.044191410738175	0.0
ZFP62-001	5.661767943137097	0.0	0.0	5.412384423752308
RBM10-006	5.737913565337193	0.0	1.2358142716723967e-05	5.48693296676677
MED24-030	4.806687563188726	0.0	0.0	4.594461679729702
CPT1B-005	4.906135145128326	0.0	0.0	4.697090742448205
ZNF208-201	4.402747643493858	0.0	0.0	4.213780173225666
APC-001	5.734063883311433	8.977686337427959e-06	0.0	5.498214993702458
GLI2-002	4.829581763570186	0.0	0.0	4.63345976429141
GABRR3-201	0.0	4.561593284825623	4.382349937016992	0.0
PPP2R5C-005	0.0	5.200145731596312	5.004875982085247	0.0
UBE3D-003	5.710144703759889	9.250587178926173e-08	0.08581846124602445	5.674933854705847
HUWE1-005	4.146919281159032	0.0	0.0	4.004165405920224
CTNND1-028	4.270212263032049	0.0	0.0	4.1280492150307095
CDH24-202	5.088046675928173	0.0	0.0	5.258848340209043
OR51T1-001	4.997185611903913	0.0	0.0015029293030942965	5.1599167272879
ECT2L-201	7.5444431056730785	0.4314314893239855	0.00989573952056368	6.601522885225005
CPNE4-002	0.0006581621448291806	4.6957900045209	4.571516445599126	0.0
PIK3C2G-201	6.183528914593572	2.259344144406847e-05	0.0	6.03378878895789
RNF220-002	0.0	4.571868962477318	4.464845603896702	0.0021526716611558125
ARMC1-201	0.0	4.883071917561008	4.767900182707074	0.0
SLC4A1AP-001	5.195889799409999e-07	5.861852282390711	5.7267764050764915	3.326457565717792e-07
CTC-518B2.8-002	0.0	4.08911302180323	4.341329711820445	0.08533980024665747
ZNF473-201	0.0	6.123461951507661	5.99985722301861	7.795793106231737e-05
OSBPL1A-003	0.0	4.9422698904713345	4.847757287013857	0.0
SLC35B2-204	0.0	4.17087110070486	4.093264736343338	0.0
RBFOX2-010	4.349945315492676	0.0	0.0	4.283837976654094
CPNE9-002	5.1733213613730085	0.1883452058263571	0.029024466074287988	4.808565990244747
RNF14-001	0.07656786780629192	5.035436443114402	4.962066725764873	2.7299113083927405e-07
EDA-001	3.846288193306994e-08	4.207684997767777	4.151421339837711	5.471422439987125e-07
ABCC4-007	4.2701584498306495	4.462462650638307e-08	0.0	4.327170089344152
FGF2-201	8.800264501227864e-08	5.779866687667003	5.891532658050019	0.03017541806635065

ZAN-003	5.987359458989816	0.0	0.01500203448840613	5.9772440550594474
GLB1L3-007	0.0	4.615794373426072	4.637362873469571	8.08245471623596e-05
CTB-50L17.10-002	4.842653834212152	0.0	0.0	4.8385185658661305
MRI1-002	0.0	2.772756043599096	4.382694983050504	0.050887432164127014
OTUD5-003	0.0	1.425088804737552e-07	0.0	4.348678460207882
LAMB4-001	0.0	0.0	0.0	4.508115727115824
ATP1B4-001	2.4980638656558902	0.0	6.13881783358652	0.0
CACTIN-201	0.0	0.0	4.431270944376783	0.0
TULP1-003	0.0	0.0	0.0	4.32815555210937
DENND1B-004	0.00753916634074445	0.0	0.0	4.146110316200255
SP140L-201	0.0	0.0	4.243271486307548	0.0
LRRC17-001	0.0	0.0	0.0	4.202583607459397
DHX58-001	0.0	0.0	4.759415725418027	0.0
MYH13-001	0.00015889579273862294	0.0005067806127457851	0.0	6.218982788375972
STARD8-201	2.730184235282205e-05	0.0	4.223059511637218	0.0
DIAPH1-201	7.643566977092552e-06	0.0	0.0026553314035845954	4.131714780592172
ADCY7-201	4.4265368840892384e-07	0.010587556197547405	5.829021030012311	0.009077471521136894
IMMT-005	0.0	0.0	0.0	4.513522619107436
SLC52A1-001	0.0	0.0	4.3789877336581595	0.0
PDE6A-001	0.0	0.36694373055936064	6.328455219030538	0.20429237768644098
KIDINS220-001	0.0	2.6786939305509374e-05	5.853561641378004	0.7298664381558843
RERG-001	2.4495031947054067e-08	0.0	4.558177641006025	0.0
RC3H1-201	0.0	0.4887755243687365	1.6220386297151288	7.4049074959447685
IL6-201	0.0	0.0	4.854338105589542	0.0
ST3GAL3-201	0.0	1.7094445550889628	4.980412830717367	0.5464351052726142
CCDC88A-001	0.0	2.6658617481719685e-07	4.689021647689096	8.926492380598383e-06
USE1-004	0.0	0.0	4.586714089850671	0.0
ABCB4-002	0.0	0.0	0.0	6.234580769636484
ABCB1-001	0.00016887130965530478	0.0	4.1472815340341285	0.1410577472796719
EPHX1-201	0.0	0.0	5.132888851033274	0.0
GABRB2-201	0.0006813273038459267	1.9023794713440543	4.002585377658911	0.0002668749286653028
CCNB3-201	0.0	0.0	0.09808581988203123	4.422858115132394
C11orf57-001	0.0	0.0	0.004048288515952366	4.183102895253642
USP25-001	0.0	0.0	6.338739218624222	0.0004840180809977462
GRAMD3-001	0.008880903852301062	0.0008429975807464958	0.0	4.589527506165553
CRAMP1-201	0.13862653031604344	0.0	8.66342845660765e-06	6.521845935803549
RAVER2-201	0.029776295819599816	0.0162444580333355	0.006788060924861865	6.099822309588906
KIF27-001	0.0	0.0	0.0	4.122436935156851
TSC1-001	2.8288645226523927	0.0	4.344430811676332	0.0
FAM35A-001	0.0388625874666397	0.0	4.549013647689674	6.126025934652018e-08
TMEM99-001	0.0	3.308513174719219	0.0	5.5242542015393346
GLOD5-201	0.0	0.0	4.199238749494965	0.0
PRY2-001	0.0	0.0	0.8146006790703216	4.956781215516111
NT5C1B-001	0.00019266164551114178	0.0	4.0374138055789155	0.0
ADH1B-001	2.65348870319824	2.5930619032668214	6.9695128497991865	1.8542936092935565
ZNF131-007	0.0	0.0	0.0	4.003492096857191
WDR49-001	0.0	0.0	0.0	4.162889404456756
ZNF699-201	0.0	2.0139275323486023	0.10819438606543397	4.768881247718794
MRPL52-017	0.0	0.0	0.0	4.122416088999534
NRF1-002	0.0	0.0	0.00013967965960244534	5.449717577732775
MCTP1-008	0.0	0.0	0.0	4.044373634539893
C9orf131-001	0.0	0.0	4.452038763194574	0.0
JAM2-201	0.10424481066357094	0.5258886664878065	7.07172932915851	0.0
CD200-001	0.0	0.0	4.002919353622762	0.0
LYPLA1-001	0.0	0.0	0.0005346202540326429	5.7580416971787765
CLSPN-005	0.0	0.0	4.957359471728171	0.03109194560194706
ATRIP-001	0.0	0.00012489673111216158	4.352328556731803	0.0
POLE-001	0.4811694054423822	0.0	5.266147730852245e-07	4.801816243366429
HACL1-001	0.0	0.0	4.280514259141983	0.0

TMEM132E-001	0.0	0.0	4.8901980145933805	0.0
NAALAD2-002	0.0	0.005906290966033154	0.0	4.09695833524435
RNF135-003	2.6651061257800412	1.3536996971628543	4.444048950573313	0.0
HPS1-009	0.0	0.11352689927271888	4.861130651736912	0.0
UBE2I-003	0.0	0.0	4.295452929078083	7.79411283199834e-06
YME1L1-005	0.0	0.0	4.530987585729104	1.8192651769591782e-06
FLII-001	0.12138851838342075	0.0	4.57691765584521	0.0
SERPINA5-201	0.0	0.0	4.821111029676154	0.0
RGPD8-007	0.0	0.0	0.00033137829822565783	4.809174802892278
DUSP8-201	0.0	0.0	5.0787175093039645	1.0426967433500283e-08
TPCN1-001	0.0	0.0	4.17990743023845	0.0
HEPH-003	0.0	0.0	2.9014893995426943e-08	4.55703542677253
AGTPBP1-201	2.9440198078097763	0.0	0.0	4.6043356627173955
RSPH10B-201	2.764434264519872	3.0303744479353556	0.0	5.109248946892578
C6-002	0.0	0.0642204817070705	0.0	4.787399770934409
NEDD4-001	0.0	0.0	2.5505596707113613e-08	5.453337190464204
SPAG8-001	0.0	0.0	4.5100473202660085	0.0
ADGRG2-008	0.0	0.0	0.13663848096587528	5.192719668121977
ACSL4-201	0.0	0.0	0.012496479391935992	6.3123203427346
NLRP7-201	0.0001999297118311131	0.00031349910434018554	2.1696498310429958e-05	4.841288664646833
SPATA13-201	0.0	0.0	5.7934575511485935	0.0
NT5C2-201	5.747397465122517e-07	8.566978137116986e-06	5.912219391678615	1.160424770759886e-07
VIPAS39-005	0.0	0.0	0.10384946213063115	5.368825545684855
TMEM130-002	0.0	0.0	0.0	4.952314102353161
TMPRSS6-003	0.0	0.0	4.147831224244398	0.0
BIN1-010	0.0	1.5217510248977013	0.0	4.19270985656129
POFUT2-003	0.0	0.0	4.832038828076928	0.0
SNAP23-201	0.0	1.9540605756103817	0.00280713610549447	4.835005513541723
IL37-002	0.0	0.0	0.0	4.603002860467087
CFAP100-001	0.0	0.0	0.0	4.114278757955888
TUFT1-201	0.0	0.0	4.6398751065249035	0.23486857670739208
WHSC1-002	0.00023731556141293856	0.0002124478680336059	0.0	5.620601076616716
BRCA1-012	2.667611931363725	0.0	0.0	4.0368116213441425
SMARCAD1-002	0.0	0.0	0.0	5.232032695778124
ZNF589-001	0.005946203114654415	0.0	4.123303502688773	0.02410288763932452
ATG7-004	2.647008293230283	3.2877762321428645	0.0	4.240624373643439
KCNQ5-202	0.0	0.14200725180282542	2.348692800359017e-05	4.838946789018789
MLH3-201	0.0	0.0	6.869951047987497	0.0
CCR9-201	0.0	0.0	0.0	5.1873713054898625
ATF7IP2-202	3.528241968313907e-07	0.0	6.0086900409678385	0.00035971743322871794
ZNF254-001	0.0	0.0	4.110556846310025e-07	4.811138738481563
AP1B1-201	0.0	0.0	0.0	5.525271551569673
RTN4-007	0.0	0.0	0.0	4.879789972767596
FN1-004	0.0	0.0	6.864726562807374	4.2509903048939334e-07
MYOF-004	0.0	0.9602213954463146	0.00048015859115257655	4.445964025951904
PRDM10-005	2.440381035526031	1.2139733473343872	0.1628605439001049	4.187114818929105
SHANK1-008	0.0	0.2136210991605131	4.476266681757394	0.008043090891517621
IKZF1-002	0.0	0.0	0.0003530052324907504	4.145843704038567
FAM86C1-002	0.0	0.0	7.899414889545689e-05	4.775136893717793
SLC25A44-001	2.674729366383458	0.0	0.0	4.431015118086065
MINA-003	0.0	0.0	4.14404848799054	0.0
ZNF317-002	0.0	0.0	4.968281885462023	0.0
MARK2-010	0.0	0.0	4.021456445297901	0.0
CHD1L-201	0.0	0.0	5.237000016552726	0.0
ASTN2-202	0.0	0.0	0.0	4.0993009578318516
CES1-004	0.0	0.0	0.0	4.627060401007266
PPT2-001	0.0	0.0	7.839762549448005e-05	5.767678761748146
EPS8L3-003	0.0	0.0	4.138238596358319	0.023923988188943063
OPA1-001	0.003822260203361548	0.0	4.951011855317535	0.0

INTS7-003	0.00020074075624322603	1.2104721435370343	6.957579062298286	1.4769870748467697
CR2-001	0.0	0.0	5.0374087626178004e-05	5.084377275933972
MDM4-001	0.002704414710453248	0.0	0.00015375438557193054	5.909446888408957
EYA4-001	0.0	0.0	4.600903609882678	0.0
CCT3-017	0.0	0.0	0.03674902692745442	4.070530134311273
RNF217-005	0.0	2.998239154611345	0.0	4.503969211420934
REV3L-010	0.0	0.0	0.0	5.251866479118863
RPF2-002	0.0	2.1365410005773824	0.0	4.167276167052499
TARS2-012	0.01030065707628217	0.0	0.0	4.383399109580027
KLHL32-001	0.0	0.0	4.486325995952476	0.008265089326461814
HIPK1-009	0.0	0.0038966547426720587	4.897849055252206	0.5539784803479159
LCA5-001	0.0	0.0	0.0	4.949819856523315
TKTL1-002	0.03443761639174509	0.0	5.477798404885671	0.0
RTEL1-001	0.0	0.0	4.459254599698784	0.0
CRTAC1-201	4.8924441827223815e-05	0.13818303486024494	0.0	4.21701489440184
MTG2-008	0.0	0.0	0.09455144872628442	4.273319617611052
ENOX2-003	5.340262476102157e-08	0.0	8.100891648735967e-05	4.0959766954039445
LEPR-002	0.0	0.019545527514162393	0.0005415091911805083	4.35288239814254
OPALIN-001	0.0	0.0	0.0	4.785768536799665
ARRDC1-AS1-001	0.0	0.0	0.0	4.2602630400510435
NSMF-202	0.0	0.0	0.0	4.1841368828133305
LONRF3-005	0.0	0.0	5.2000777812839205	0.0
CCSER2-201	0.0	0.0	4.174779962824852	0.0009074551630123245
EXOSC2-006	0.0003229169182002709	8.752180774868438e-07	5.640957779733353	0.0
CAP1-004	0.0	2.752036582190711e-08	0.0	5.083836742314942
TRIT1-002	0.0	0.0	8.510970626675068e-05	4.4095769676673395
UBR2-001	4.085559235433344e-06	0.5316259180917285	6.995133112984008	1.5969548101053905
TRERF1-001	0.0	0.0	1.1552401439063334e-07	4.713302316675912
TBX22-201	0.0	0.0006490177964672659	0.008908490461345162	4.706257584151427
PBX3-010	0.0	0.0	0.0	4.450425717271579
MAPKAP1-009	0.0	0.0	5.683665229651419	0.008311396370532026
HDAC8-001	0.0	0.0	4.686436838379338	0.003808260481807033
ITGB1BP2-001	0.0	2.2621436508167423	0.0	4.019906771457426
MAPK8-014	0.0	0.0	0.0	5.6387216941550955
DLG3-001	0.0	3.194437007150213	4.470890047395256	3.914972573237301e-05
MUSK-001	0.0	0.0	4.517029290486178	0.0
RAB41-001	0.11493141381852821	0.6926221352868256	5.235370033208312	0.7142220779636976
DAXX-001	0.0	0.0	4.359785808317391	0.023802834595644223
CUL2-001	0.5497053630646057	9.607720596193591e-07	0.0	4.503328995362116
SMC2-001	0.0	0.0	0.0	4.32069361376738
ITGAV-003	0.0	0.0	4.8424348344464905	0.0
C6orf10-201	0.0	0.0	1.4133996257516133e-08	6.241015481932025
DDOST-001	0.0	0.0	4.926513468848042	0.010517790580097134
ZNF341-201	0.0	0.0	5.055065133041852	0.0
ARHGEF10L-003	0.0	0.0	0.0	4.067057604547329
ARHGEF7-007	0.0	0.06194034679413316	5.350396497821944	0.0
PDPN-002	9.556897686078776e-06	0.0	5.674449093338634	0.8799672368037756
PCCA-001	0.0	0.0	0.06938707475870494	4.544347580606533
CBWD5-013	0.0	0.0	0.0	4.274154906955416
BORA-005	0.0	0.0	4.006358637218737	0.0
WISP1-004	0.0	0.0	4.1003485228771215	0.00033849157860596914
OPTN-008	0.00020078809829926698	0.0	1.0636173366093227	6.579634682103435
LGR4-001	0.03465760620694561	0.018150540058252493	4.156056466089548	0.003758105859114999
BRE-002	0.0	0.0	4.3626470311148235	4.2463882893612436e-07
LINGO2-001	0.1710880227192567	0.7421641102644279	0.0	5.0368336127660145
PSIP1-004	0.0	0.0	4.334415870021486	0.0
DNM1L-010	0.0	0.0	5.1835571292332165	3.3125231071316695e-05
GEN1-201	0.0	0.0	5.668865141482223	0.47399731486374846
NOMO2-002	0.0	1.1391048318596946e-07	4.185586003919752	0.004314217665913852

E2F6-001	0.0	0.0	4.376285556146171	0.0
TMPRSS6-201	0.0	0.0	0.5452784021392948	5.452804671281606
DEPDC5-006	0.0	0.0	5.8536557466154315	0.0
IFITM10-004	0.0	0.0	4.208107881888913	0.0
RBCK1-006	0.0	0.0	4.429740538567603	0.0
DAZ2-201	0.0	2.798487731865515e-05	0.0	4.055334414674903
WDR1-202	0.0	0.0	4.411478886447446	0.0
RBMY1D-001	0.0	0.0	1.088475015077909e-06	4.377419687196744
CDY2B-001	0.0	0.0	5.20013269414651	0.0
PLSCR4-201	3.459358487137748e-05	0.0	0.0	4.5761572358848674
LTN1-001	9.033537086771883e-07	7.539303749482643e-06	4.736815627675039	0.00926309157520071
PTPRN2-004	1.6979510825200637	0.0	5.939889228810516	1.12285487376988e-06
STK36-002	0.0	0.03735288144852541	6.17025947683845	0.004879523379071184
OPA1-002	2.8222702441650114	0.0	0.0002912315590482648	6.607036557836164
C17orf77-001	3.8072255474870834e-08	0.0	0.0	4.648347292029349
CERS6-002	0.0	0.0	5.792292399164646	0.0
ECT2-003	0.0	0.0	0.0	4.410277234508395
ORC4-001	0.0	0.05416783244655949	0.006420089902521132	4.6151465935494755
NRF1-202	0.0	0.0	4.156213995813749	0.0
EME1-201	0.4332815830352338	0.0	5.381586578221883	2.98649854483899e-07
RAB43-003	0.0	0.0	4.036802555279939e-05	6.154325283419162
ODF2-018	0.0	0.0	4.342671312247959	0.0
ARAP1-007	0.015202515499794851	0.0	0.0	4.36376446700184
SLC26A5-013	0.0	0.0	4.414062485088939	0.0
MBNL3-201	0.0010669746322833989	0.0	4.1146963338157	0.0
CAMK2D-001	0.0	0.00019156778293462416	4.7329493759366414	0.6870252550626958
PTBP1-002	0.0	0.0	4.917355853488714	0.0011859064313139596
SEPT6-201	0.041891051235886954	0.0456075284535905	4.145814668715839	2.9081808292176685e-05
KTN1-201	0.001906371113255774	1.9786119337769013	0.004802431293200419	5.052973799320286
CRCP-001	0.0	0.7952459607754124	0.0	4.201365412982124
AK4-201	0.0	0.0	6.302558849609845	1.9617057021167414
PPHLN1-003	0.0	3.428808357750185e-06	5.000619144665264	1.6124660298632497e-07
ATRX-002	0.0	0.0	5.881341291512267	0.5835534726042289
ZNF286A-002	0.0	0.0	5.484645778380873	0.07292800556172117
ZNF586-001	0.036775293803963056	8.388812654480462e-07	0.0	4.201848868307936
ADCYAP1R1-003	0.0	0.0	0.0	4.102622861669856
STIL-008	0.0	0.0	0.0	4.296568401726722
ATF7IP2-002	0.0	0.0005235748685181582	4.63682674007294	0.0
TRIM40-001	2.2669844179502023	0.0	5.453796678741278	0.0
FNDC3A-002	0.0	0.0	0.0	4.212365759236267
CDH23-201	0.0	0.0	6.302651969791411e-06	4.72978878619343
GRB10-202	0.0	2.3858860281533834	0.0	4.431313852308
NRIP1-002	0.0	0.0	1.7805224218527663e-08	5.1918731086603565
DEPDC5-201	0.0	0.0	0.0002977943531804298	4.019979509220455
KMT5B-010	0.0	0.0	0.4588984301091458	5.187607937602465
STON1-GTF2A1L-003	0.0	0.0	0.0	4.163345398688351
MADD-009	0.0	0.0	4.383876771345685	0.0633338056634094
FMO1-201	0.0	0.0	0.0	4.1832577908202335
KDM5C-009	0.0	0.0	4.664347900800099	0.0
NRG3-005	2.2225036969532255	0.0	0.0	4.177951901134501
RSPH10B-001	0.0	0.0	6.082867295043596	1.125332283753949
PPP1R7-002	0.0	0.0	0.0	4.2078489587525
KAT6A-004	0.00041665774090240086	0.7351637031689527	7.77662333140525	1.3626950657076113
EIF2A-003	0.0	0.0	4.830855092610829	1.3990578854338261e-08
SIRT1-002	0.0	0.0	4.2807355347233935	0.0
SEPT2-005	8.951074091491571e-08	0.0	0.0	5.911702793810608
UMODL1-002	0.0	0.0003597335282585335	4.619646534077942	1.3306539667114576e-08
MARK2-016	0.0	0.0	0.0008653170462096832	5.435730739651826
ARHGAP25-006	0.0	0.0	4.444480331278038	0.0

FAM131B-004	0.0	0.0	0.0	4.722208869684566
TMEM139-003	0.0	0.5155675090951429	4.524175994052444	5.938322530012779e-07
OSBPL6-005	0.0	0.0	4.549718334471834	0.0
XIRP2-006	0.0	0.0	5.287140765193613	0.0
DYSF-004	0.0	0.0	6.012528961598391	0.34400042661277747
CALCRL-002	0.0	0.0	0.0	5.088915647942304
PRR29-001	0.0	4.372229368532566e-08	4.984175128288676	0.0
DYSF-021	0.0	0.0	9.853276526970236e-06	6.708805653009109
KIF27-202	0.0	0.0	5.97781419868708	2.0056839060687872e-05
SCN5A-004	0.0	0.8575734367102632	6.255671441970573	6.1559396269084735e-06
ZNF200-001	0.0	0.10865160806620154	0.0	5.568262591481185
DDX11-016	0.0	0.0	4.071838795394904	0.0
DAZ4-202	0.0	0.0	0.0	5.424439849285133
TMPPE-002	2.622118066944862e-08	0.0	4.142726191471213e-05	4.8338156174095035
CAPRIN2-005	0.0	0.0	4.29891275757047	0.0
FAM138A-001	1.6364922959373225	0.0	0.9832632590845077	6.426102237921506
DIP2A-012	0.0	0.04111128932945557	5.4419614150310816	0.0
RAPH1-013	0.0	0.0	4.290693847939947	0.0
TMEM50B-002	0.0	0.0	0.0	4.330318496354406
M1AP-007	0.0	0.0	0.0	4.1036739092596255
PLB1-201	0.6818499740343049	0.0	6.157282955539523	5.047266820637452e-06
ITGB1-202	0.0	0.0	0.0	4.404202648891014
DCAF1-001	0.0	5.278358984106593e-08	0.0	6.302486826754035
GREB1L-201	0.0	0.02832454481789338	0.0	5.085096781179278
LPIN1-202	1.0131903504420321e-07	0.0	6.059883456331042	5.851900903110628e-07
ZNF418-003	0.0	0.0	4.004139624623248	0.0
LIG1-002	0.0	0.0	0.08832784459233138	4.422151228190436
SLC30A8-201	0.0	0.0	0.0	5.99271305789029
CDC7-201	2.689075522913172	0.0	4.469963607632763	0.0
IL2RB-005	0.000657558102104669	0.0	3.142314413410564e-07	5.212975138672466
ALS2CL-003	0.0002265143307929162	0.5902302038287741	5.018121577741242	0.010071766332286677
TBL1XR1-014	0.0	0.0	4.107565721656487	0.0
NHEG1-001	0.0	0.27036334857187844	5.010765530352446	0.0
WHSC1L1-003	0.0	0.0	4.242877519049051	0.0
LILRB2-201	0.0	7.715964952367693e-07	4.390173111083807	0.0
FAM160A1-201	0.0	0.0	4.151939832736778	0.0
NPIPB7-201	0.0	0.0	4.793007566133509	3.25150922162084e-05
PANK4-008	0.0	0.0	0.0	4.453768131576476
TAC4-005	0.0	0.0	4.259561338809169	0.0
CDK14-201	0.0	0.0	1.012011993397156e-06	4.323108128366426
GLI2-001	0.0	2.9653516092444123	4.439817006040514	0.0
KIF15-002	0.0	0.0	5.72942703028982e-06	4.125778845582176
HPS5-005	0.0	5.332421130447058e-06	0.32069804844067684	5.856509682933978
CPB2-201	0.0	0.0	3.811404100701176e-05	4.991615106009155
LONRF3-002	4.0106322921704095e-06	0.0	0.0	4.680171023162203
GFRA1-201	2.91191793717062e-06	2.0218634046764844e-05	4.525114020617045	2.8374978918202036e-06
OSBPL2-201	0.0	0.0	4.031994455638704	0.0
BRPF3-005	3.429641692071006e-05	8.584178335661028e-07	4.809217291575323	0.0
MORF4L2-006	0.0	1.8522922396232762	4.124375797159652	2.3266496161231198e-07
PRUNE2-203	0.0	3.033000643517775e-07	0.004545106846314317	4.0754164431513145
ATIC-002	2.048621772403206	2.948038853676129	0.0	4.080431133249979
AC023274.4-001	0.00015313026509015406	0.00043959116380375585	4.181136863744307	7.8674517547976e-05
PTPRO-006	1.3142898599952388	2.3536378809993344e-07	0.002678389156717458	5.6824103567184405
FAM219A-201	0.0	0.0	0.30571186994126737	6.084147110049389
SLC5A6-008	4.241690321621228e-07	0.0	0.00029117803165705947	4.966568574705404
FNBP1-002	0.0	0.0	5.168224848084236	8.629087847006e-06
DNAI2-201	0.0	0.0	4.034267491655506	2.0535319251839993e-06
MPDZ-009	0.0	0.0003402776056267604	0.10102984837007689	6.530394657516891
CHTF8-001	0.0	0.0002789944466819564	0.0	5.627618548466604

AP2A2-001	0.0	0.0	0.0	4.464666194219402
SERPINA1-203	8.305772285635526e-05	0.0	4.52026097187156	0.006453074607238545
MORF4L2-204	0.0	0.0	4.874647473305788	0.0
CITED1-003	0.0	2.322511844288225	0.0	4.208963533981529
SEC16A-005	0.0	0.0	0.0	4.131502463095341
SMPD4-011	0.0	0.0	1.196008217801733e-08	4.132724155834835
GABRR1-001	0.0	0.0	0.0	4.754022532356096
USP9YP10-001	0.0	0.0	8.53970329887078e-06	4.735210865866542
FOXP3-202	0.0	0.0	0.0	5.435697185436547
PCCA-AS1-003	0.0	0.0	5.023981687269388	0.0
TP63-013	0.0	2.212313212581665	5.854019751243203e-05	4.170813292370081
DARS-002	0.0	2.282733112099323	0.0	4.129740576439237
TRMU-003	0.0	0.0	5.0919202691690595	1.592941877522823e-05
ZSCAN21-004	0.0	0.0	0.0	4.225419527886964
MAPKBP1-002	0.0	0.0	5.5701727975322335	0.009906714124620521
YWHAZ-202	0.0	0.0	4.779259320124768	0.0
MTTP-201	0.0	0.0	4.2759547578727695	0.0
ELN-009	1.7997502124633062e-05	0.0	0.0	4.107349190612275
LGALS17A-002	0.0	0.0	5.069713917442196	0.0
CDK11A-002	0.0	0.0	0.0	4.40266283177152
PLOD2-005	0.0	3.291402042107191	0.0	5.0452644353800915
C9orf72-004	2.27996890257957	0.0	4.238955159032761	0.0
BSPRY-002	0.0	0.0	4.820753932463339	0.0
SASS6-002	0.0	0.0	0.0	4.733887955632092
AKR1E2-002	0.0	7.966345160387695e-07	1.0155757018713063e-08	4.444530697620859
VPS52-005	0.0	0.0	4.131584250383007	1.7257452395665526e-05
RPP40-006	2.670733188209484	2.7521688011228087	0.0	4.037906180156809
IGF2BP3-013	0.0	3.0329585069146363	0.0	4.019145602607002
PRRT1-007	3.0067811672186295e-06	2.656316600342196	4.095196862312682	0.0
PDXK-010	0.028001115000677215	2.8602027974890465	4.959203024925882	0.16960982845202094
OR2A1-AS1-016	0.0	0.0	0.0	4.584810930896111
WDR46-004	2.444373674223411	0.0	0.0	4.05886792044594
FBXO24-005	0.0	0.0	0.0	4.761782212738048
INTS7-002	0.0	0.1483133006553335	0.0	4.394770721365341
SLC7A2-002	1.2516184515486666	0.0009599972903343656	0.1730531119835912	4.825830059612395
CD247-006	0.050889369998507926	0.0	4.086052977771247	0.0
ZNF286A-005	0.0	0.0	4.007084813597329	0.0
TMEM209-008	0.0	0.0	4.428685404372715	0.0
SNRPC-004	0.0	0.0	0.0	4.053455080405595
ZEB2-015	0.0	0.0	4.037050296828404	0.0
PQBP1-005	0.0	0.0	0.00011221871745496324	4.982433264069293
KRI1-001	0.0	0.0	4.116984464772552	0.0
ECHDC1-015	0.008085382500094777	0.002348604112440742	4.444657684054692	0.0
METTL2B-008	0.0	0.0	4.029298628456609	0.0
NOBOX-001	0.0	0.0	5.092861838980808	0.09686975157618932
DRAM2-003	0.0	0.0	4.261855218258585	2.636080701882199e-06
FAM71F1-003	0.0	1.387251967706235	4.394757448192108	0.0011477324257378274
CNKSR2-001	0.0	0.0	4.081347743039909	2.7424647304202806e-07
RABL2A-018	1.1884067750922498	0.0	4.0311787208312015	2.921843682828756e-08
FAM185A-007	0.0	0.0	0.0	4.488537194134883
ATP11A-001	0.0	0.07289146191899444	4.567306004135701	0.010929715535567867
FETUB-008	0.0	0.0	0.0	4.026867358936869
TFDP2-001	0.0	0.0	0.0	4.181518007908188
TSN-003	0.0	0.0	5.08920015934003	0.042831909627427577
LSM2-006	2.3104486694381534	0.0	0.0	4.210013490972608
MCFD2-016	0.0	0.0	4.269511654768802	0.0
LGALS8-AS1-201	0.0	0.0	0.0	4.287268239556365
TTC12-004	0.28490080760854164	0.40703695294180636	5.257551348884783	0.11891560898133036
AIDA-006	0.0	0.0	4.018995303299161	0.0

HLA-B-002	0.0	0.0	0.0	4.700592736152664
PIAS3-007	0.0	0.0	0.000266837780277859	4.797863442350903
FILIP1-003	0.0	0.026021894616179847	4.470577341512899	0.0
ZNF518B-006	0.0	0.0	5.863133502159533e-07	5.422684501312569
TSC22D1-201	0.0	0.0	5.034054053041211	0.0
FGB-011	0.0	0.0	4.277272522645107	0.0
NDUFAF2-003	0.0	0.0	4.006172939465615	0.0
CC2D2A-002	0.007540178617156057	0.0	0.010743630869846752	4.802440921829958
RFESD-006	0.0	0.0	0.0	4.380212006286213
HSFY2-005	1.2865590133188107	1.89615304778527	4.182036493522902	0.0
ZBTB47-003	0.0	0.0	4.695360164778942	0.0
USP17L20-001	0.0	0.0	5.247041904655938	0.0
RAP1GDS1-011	0.0	0.0	4.464754187721271	0.0
RAB28-003	2.7295469417245375	0.0	0.0	4.036971987430202
TRIM41-003	0.0	0.0	4.116605048057551	0.0
ZFP42-002	0.0	0.0	0.0	4.178643773774552
UGT2B4-004	0.0	0.0	0.0	4.654105262816957
PCDH18-005	0.0	0.0	4.853743295779908	0.0
ADGRL3-014	0.0	0.0	0.0	4.588520534092486
SERF1A-008	0.0	0.0	5.375218736510501	4.153813028760794e-08
MAP1B-002	0.0	0.0	4.18285824540441	0.0
GABRA2-011	5.3653115761396016e-05	0.0	0.5323347648161743	6.683216079084037
LINC00900-003	0.0	8.114299710388389e-05	4.218276739322195	0.0
CDK10-010	0.0	0.0	5.580207620777784	0.0
OXR1-006	0.0	0.0	4.245540496377024	0.0
TEK-003	1.5207159902282767	0.0	4.498670305170842	0.0
MTSS1-006	0.0	0.0	3.409886450066243e-05	4.879133139164011
EFR3A-002	0.0	3.3013116787856838	5.773676467688447	0.0007297784518923607
AMPD1-001	0.0	0.0	4.655212651761124	0.0
ANK1-007	0.0	6.149059226413503e-07	4.020991584089544	0.0
TOP1MT-011	0.0	0.5182191787278336	4.0759975670344835	0.0
RGPD8-008	0.0	0.0	0.008177733585425043	4.6664537357436755
KCNU1-002	0.0	0.0	0.0	6.160442130381537
SMG1P3-001	1.1525880742412328e-06	0.0	4.242719902017335	0.0
CEP57L1-006	0.0	1.2004073042728192	4.480479695414357	0.0
TSNARE1-001	0.0	0.0	0.0	4.053509746778696
ADGRB2-014	0.0	0.004141168378418579	4.209447750606623e-06	4.642973202181218
C6orf10-005	0.0	0.0	6.275905994753853	1.0576607621484234e-05
POLD4-006	0.0	0.0	1.1921064066120252e-05	5.223411525320023
GLI1-003	0.0	0.0	0.0	5.248606387344873
ZNF143-008	0.0	1.7949425270963837e-05	0.0	4.905130548286481
RDX-005	1.7359528055841364e-07	1.2218566067715429e-06	0.0	4.0229586043648755
PSMC3-002	0.0	0.0014712696727794128	4.034749515292465	0.0
BIRC3-001	0.0	0.0	5.146172393662508	6.325060671084066e-06
LUZP2-001	0.1238910998489257	0.1451720091353519	4.86608378024827	4.0974959390093646e-05
BOLA3-AS1-003	0.0	0.0	4.97978158111357	0.3969718576504003
FAM111A-003	0.0	0.0	0.0	4.897704723309459
SLC5A9-007	0.0	0.0	5.030167309346431	0.0
PHRF1-004	0.0	0.0	4.973135333700393	0.0
C8B-201	0.0	0.16779448422791354	4.9703943303293165	0.0
DAP3-201	0.0	0.0	4.364314171192227	0.0
KIAA0319-202	0.0002077583685381264	0.0	6.48148481347539	0.0
ZBTB7B-202	0.0	0.0	0.0	5.440015067213569
TSN-201	0.0	0.10004231944890564	0.0007662501806437303	5.827419766825428
CRNKL1-007	0.0	0.0	4.105094298648542	0.0
MELK-202	0.0	0.0	0.0	4.819112557333164
FAM65B-201	3.3256569754564932e-06	0.0	0.015635796651808156	6.397219419138125
GABARAPL1-005	0.0	0.0	4.5047064244431665	0.0
ARHGAP5-001	0.0	0.0	0.0	5.3243182971888645

SOAT1-202	6.322019952677351e-08	0.016493049208302475	6.4651167849678215	0.001170642392048436
EOGT-202	0.0	0.0	0.0	4.27682646064261
ARMC10-202	0.0	1.062953457842853e-06	5.531506298953554	0.0
DAG1-204	6.004709384680207e-06	0.0	0.0	4.153178051769662
MELK-203	0.0	0.0	5.58774066586146	0.0
YTHDF2-008	0.0	0.0	0.0	5.4826032307041
SERPINB8-202	0.0	0.0	0.0	4.620665732463528
NLRC5-008	0.0	0.0	0.0	4.0585193171371134
PRAME-202	0.0	0.0	0.0	4.735469850596727
TIMM50-201	0.0	0.0	0.0	4.380421212510543
CDY2B-201	0.0	0.0	4.076585497984778	0.0
SRPX-203	2.0841908655008665	0.0	0.018846351911937415	5.28686130097233
BTAF1-201	0.0	0.0	3.1188160136136187e-06	5.3662465616488815
CDK2AP1-004	2.8967408739352267	2.192829635184194	4.524141664439905	0.0
NUDT13-202	0.0	0.0	0.0	4.009602587194432
ASH2L-201	0.0	0.0	0.0	4.423090846410611
E2F6-203	0.0	4.748009352877756e-08	0.0	5.930752513312505
PRB1-201	0.0	0.0	6.0860102742795466	0.012760870865409377
SART3-003	0.0	1.5052699645470367	4.430843733765848	0.0
PPFIA2-011	0.0	0.0	4.111460438047044	0.0
GLYCAM1-003	0.0	0.0	4.464563387476858	5.077882240120312e-07
OR13A1-001	0.0	0.0	0.0	4.257508406898086
RP11-1085N6.2-001	0.0	0.0	0.0	4.266473636804782
PTGR2-004	0.0	0.0	0.0	4.06059335541089
MPP5-004	0.0	0.6975728672112809	6.019242888341008	1.0029759662708016e-07
RP11-47I22.4-001	0.0	0.0	0.0	4.2247788608347046
RNASE9-004	0.00026924329375985873	0.0002631817178007021	0.0	6.092897258507829
NUSAP1-007	0.0	0.0	4.957040210263977	0.40683985187515764
RP11-403B2.5-001	0.0	0.0	4.675996107244267	0.0
NIP7-008	0.0	0.0	4.172167998281453	0.0
SLC7A6-014	0.0	0.0	0.0	4.310747978505204
RBMX-009	0.0	0.0	0.0	4.106443104310419
AC004381.6-004	0.0	0.0	0.0	4.374013602455721
ADGRG1-003	0.0	0.0	4.11656457105122	0.0
IGHV1OR16-2-001	0.4550931604265054	0.9036087844617146	5.604873571533695	0.2878212529911298
CENPT-006	0.0	0.0	0.0	4.208801556702279
ADGRG1-008	0.0	0.0	4.000903373117636	0.0
CIAPIN1-004	0.0	0.0	5.744182068759625	0.24008947522690516
ERI2-004	0.0	0.0	4.042525291528628	0.0
SLC43A2-003	0.0	0.0	0.0	4.488808082125541
RPL23AP87-001	0.0	0.0	0.0	4.733239696036657
CAMTA2-006	0.0	0.0	0.01304358521160269	4.736549588435587
CLUH-017	0.0	0.0	4.801536521365691	0.003714006373975956
P4HB-014	0.0	0.0	0.0	4.005641059922564
ZNF594-001	0.0	0.0	5.275237215211741	0.0
MYH11-003	0.008586079673959733	0.0	4.0836013378027465	0.0
FLOT2-004	0.0	0.0	0.0	5.12629337734937
LRRC46-004	0.0	0.0	4.049427409829048	0.0
PER1-005	0.0	0.0	4.160716006290619	0.0
NBPF15-202	0.0	0.0	4.13532828124322e-06	5.314500350834229
CILP2-001	0.0186381013642546	0.02695126008433195	0.0033999403792765955	5.362114546270025
LPAR2-001	0.0	0.0	1.9250931221817874e-08	5.123283434128919
LGALS3BP-004	0.0	1.9509078875481463	4.132169984894025	0.0
TRMT1-012	0.0	1.7108130636475782	0.0	4.211121495076141
NFIX-004	0.0	0.0	0.0	4.714727193791772
KRT9-002	0.0	0.0	3.833988055799226e-06	5.094308906247891
ERC1-202	2.5101076347005007	1.8762699417203619	5.487796229733289	0.10684617405672885
RTN2-005	2.9509600132066276	0.0	4.095299562526804	0.0
NFIX-013	8.135637030078479e-07	1.8910773482363046e-06	4.779204983758403	0.0

PLIN3-007	0.0	0.0	0.0	4.1024009346375845
LIG3-006	0.0	0.0	0.0	4.028848356698843
LIG1-004	0.0	0.0	5.809114475277348	0.0
RP11-78B10.2-002	0.0	0.0	0.0	4.224885658436008
FAM223B-001	0.005998174869950805	0.010255919340476427	0.00035713016901881104	4.153274979466965
KLK6-005	0.0	0.0	4.332770422046769	0.0
KIAA1683-003	0.0	0.0001892499366165761	4.342411744997124	0.0
MARCH2-005	0.0	0.0	4.599090456621563	0.0
NXF2B-001	0.0	0.0	6.638095279315242	0.0
KDM5B-010	0.0	0.0	4.4739027989667965	0.0
ANKRD30A-001	0.0	7.451235405231948e-07	0.2839919612622401	6.266989816637936
LINC00993-003	0.0	0.0	4.197302400998361	0.0
RNF146-011	0.0	0.0	0.34820981875264156	4.444614069664033
ALG13-202	0.0	0.0	5.354128501348234	0.0
GRID2-201	0.0	0.0	4.541499362699713	0.0
ZNF254-201	0.0	0.0	5.269985573211377	0.35003351190012977
ZNF16-201	0.002391677348333778	0.0	0.000293577109837834	5.119290850143608
BCL2L13-205	0.3949115238203772	0.0	5.047115281388394	6.567246129733783e-07
Metazoa_SRP.83-201	2.241498536735161	2.288211536572213	4.331284424582104	1.5177673688018998e-05
HECTD1-202	0.0	0.0	6.0908342773294715	0.00013432537935597298
RP11-402G3.3-001	0.0	0.0	0.0	4.654714392531369
HLCS-201	0.0	1.8586997189151174e-06	5.318913015433247	0.0
RN7SL422P-201	1.0563001157855583	0.0	0.0	4.236002652289918
PDE6A-201	0.0	0.0	4.473274673172652	0.12872382001342905
FGFR3-204	0.0	0.0	0.0	4.109943138090099
MR1-202	3.0704346732346087e-07	0.00012608908930716395	4.369911200861946	1.7727821621675879e-06
ZSCAN26-202	0.052697094801558486	0.0	0.0	5.606734874238954
MTERF4-201	0.03989318463863748	0.0	0.0	4.85188561686467
PARD3B-202	0.0	0.0	4.161858930589952	6.195413398933282e-05
C8orf58-201	0.0	0.0	0.0	4.759173472549541
RP11-144A16.8-001	0.0	0.0	4.6245293140804735	0.0
RP11-423C15.3-201	0.13502365958269352	1.0056047500814556	1.4322745531974566	7.040875872169501
GTF2I-002	0.6842842431172255	0.0	0.0	4.115405145005415
IKZF1-207	0.0	0.0	6.677513665158312	0.0
YAP1-203	0.2395270537275565	0.0018412593685461628	6.13875555410655	1.665157294795843e-05
YTHDF3-003	0.0	0.0	0.0	5.909274721354983
COL12A1-201	0.0	0.0	8.22521643298134	1.212706161760458
AUTS2-202	0.0	0.0	5.701318987703492	0.13474440899776102
MCM3-202	0.0	0.014073329461786082	4.270644346366847	0.003867719515713011
INO80-202	0.0	0.0	0.011559157975298615	4.130383396163618
BCL2L13-206	1.990341342407795	0.0	0.0	5.301195989375491
SLC2A14-202	0.0	0.0	0.012408944087599662	4.365258261639985
PRKD1-201	0.0	0.0	9.75518277235591e-07	4.839767987389697
NOP2-201	2.502288798424947	0.0	0.0	4.352189258389643
ASAP3-201	0.0	0.0	0.0	5.177482826614563
SNCA-203	0.0	0.0	4.881476307531691	0.0
KCNE1B-001	8.72971919162929e-08	0.0	4.303124765965065	0.0
LYPLA1-201	0.0	0.0	4.842346113305251	0.06952915264779788
ROBO1-203	4.061682965648248e-08	0.0	4.693210838351582	0.0
ADAM17-201	1.1175749446696763	0.19523817164772395	0.32232958867599437	4.815609892237514
BRF1-202	0.0	0.0	4.0948116713843	0.0
ZNF568-203	0.0	0.0	0.0	4.208187841440713
KIZ-009	0.0	0.0	0.0	4.427231412733665
NCAM1-003	0.0	0.0	4.473598646411958	0.0
FAM184A-202	0.0	0.0	4.246937172064482	0.0
SMOX-201	0.0	0.0	4.097700662798635	0.0
MYC-001	0.0	0.0	0.012955213268575428	4.92879452246387
GTF2I-003	2.5191948966237996e-06	0.0	0.0	4.592303408711056
ZNF322-203	0.0	0.0	4.356293413680463	0.00018580777146247744

SIX5-201	0.0	0.0	4.039939798505995	0.0
CTD-3137H5.4-001	0.0	0.0	4.266842448477265	0.0016343866843173739
CH507-152C13.4-001	0.0	0.0	5.132083180410704	0.0
FAM223A-001	0.005998174869950805	0.010255919340476427	0.00035713016901881104	4.153274979466965
CUL2-202	0.0	0.0	4.188130476958203	0.0
IMPDH1-201	0.0	1.0880468302640745e-07	0.3438375067669859	4.545914419394067
PROX1-AS1-032	0.0	0.848142339681118	5.295567724004891e-05	4.641595636263471
HIVEP1-202	0.0	1.705001970830031	6.661285782274188	1.0110835405433576
PYCR1-202	2.71406224810089	0.0	0.0	4.662767446108696
SYNJ1-014	0.0	0.0	4.074789736112044	0.0
SMARCA2-041	0.0	0.0	0.0	4.065421214074135
FOXP2-028	0.0	0.0	4.502791394550548	0.0
FASN-004	0.21292992311534048	0.0017654519168084316	0.0	4.372682214202622
COL11A1-009	0.005099935503879216	0.0	4.462005023118136	0.0
ABCC8-019	1.927632352995889	0.0	4.394085544398347	0.0
PCCA-010	0.0	0.0	4.05075198135549	0.0
KIF16B-005	0.0	0.0	0.0	4.617526145883926
CACNA1A-053	0.0	0.0	4.769519668487567	0.0
ALDH7A1-028	0.0	0.0	5.987202762058905e-08	4.8325068714525425
TDRD12-007	0.0	0.0	4.966823222665133	2.9047761287292724e-06
CACNA1A-016	1.2668456570060792e-06	0.0	0.0	4.957809677118829
MBD5-022	0.0	0.0	0.0	4.632731829765186
