# Supplementary material for: Extracellular vesicles produced by irradiated endothelial or Glioblastoma stem cells promote tumor growth and vascularization modulating tumor microenvironment
Source: Cancer Cell Int. 2024 Feb 12;24:72. doi: 10.1186/s12935-024-03253-0 (PMC10863174; doi:10.1186/s12935-024-03253-0)
Supplement: Supplementary file 3 — Additional file 3: Figure S2. Validation by qRT-PCR of enriched transcripts in EVs isolated after irradiation from GSC lines. A–C. qRT-PCR analysis of CENPJ, CNTROB, FLNB and LMNB in GSC-derived EVs isolated from GSC#1 A, GSC#83 B and GSC#163 C. EVs were isolated from sham irradiated (EVs) and irradiated cells (EVs 50 Gy). Samples were run in duplicate. Data were normalized to the GAPDH expression in the corresponding samples. [file 12935_2024_3253_MOESM3_ESM.pdf]

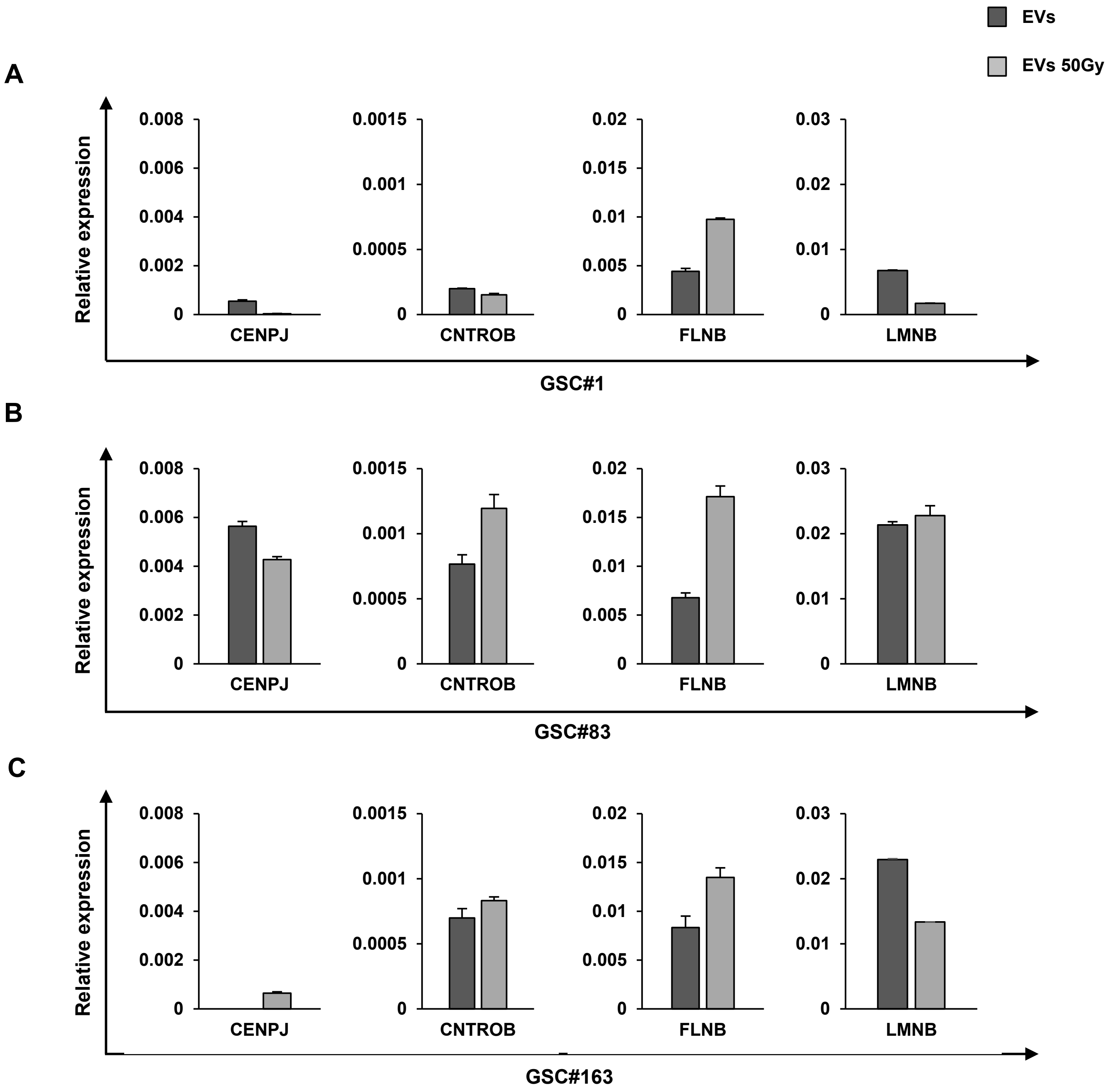

Additional file 3: Figure S2. Validation by qRT-PCR of enriched transcripts in EVs isolated after irradiation from GSC lines. A-C. qRT-PCR analysis of CENPJ, CNTROB, FLNB and LMNB in GSC-derived EVs isolated from GSC#1 (**A**), GSC#83 (**B**) and GSC#163 (**C**). EVs were isolated from sham irradiated (EVs) and irradiated cells (EVs 50Gy). Samples were run in duplicate. Data were normalized to the GAPDH expression in the corresponding samples.
