# Supplementary material for: Extracellular vesicles produced by irradiated endothelial or Glioblastoma stem cells promote tumor growth and vascularization modulating tumor microenvironment
Source: Cancer Cell Int. 2024 Feb 12;24:72. doi: 10.1186/s12935-024-03253-0 (PMC10863174; doi:10.1186/s12935-024-03253-0)
Supplement: Supplementary file 4 — Additional file 4: Figure S3. Effects of POSTN silencing on GSC tumorigenic properties and endothelial transdifferentiation potential. A. qRT-PCR analysis (left) and WES full-length gel image (center and right) of POSTN expression in sh-NTC or sh-POSTN GSC#61. For qRT-PCR, samples were run in duplicate and data were normalized to the GAPDH expression in the corresponding samples. For WES, the relative amount of each immunoreactive band was shown, the protein expression was quantified using AUC measurements and signal intensity was normalized to the β-actin expression in the same sample. B–D. Functional in vitro assays on sh-NTC or sh-POSTN_C GSC#61. Growth curve B: points and range lines at each day represent mean ± SD of at least two independent experiments in triplicate. Migration C and Colony formation D assays: values are reported as percentage relative to sh-NTC GSC#61 and shown as mean ± SD from two independent experiments in triplicate. E. Transdifferentiation assay: cytofluorimetric evaluation of CD34 expression. Values are reported as fold change relative to sh-NTC GdEC#61. ns, not significant; *p<0.05; ***p<0.001 vs sh-NTC vector. (Student’s t test). [file 12935_2024_3253_MOESM4_ESM.pdf]

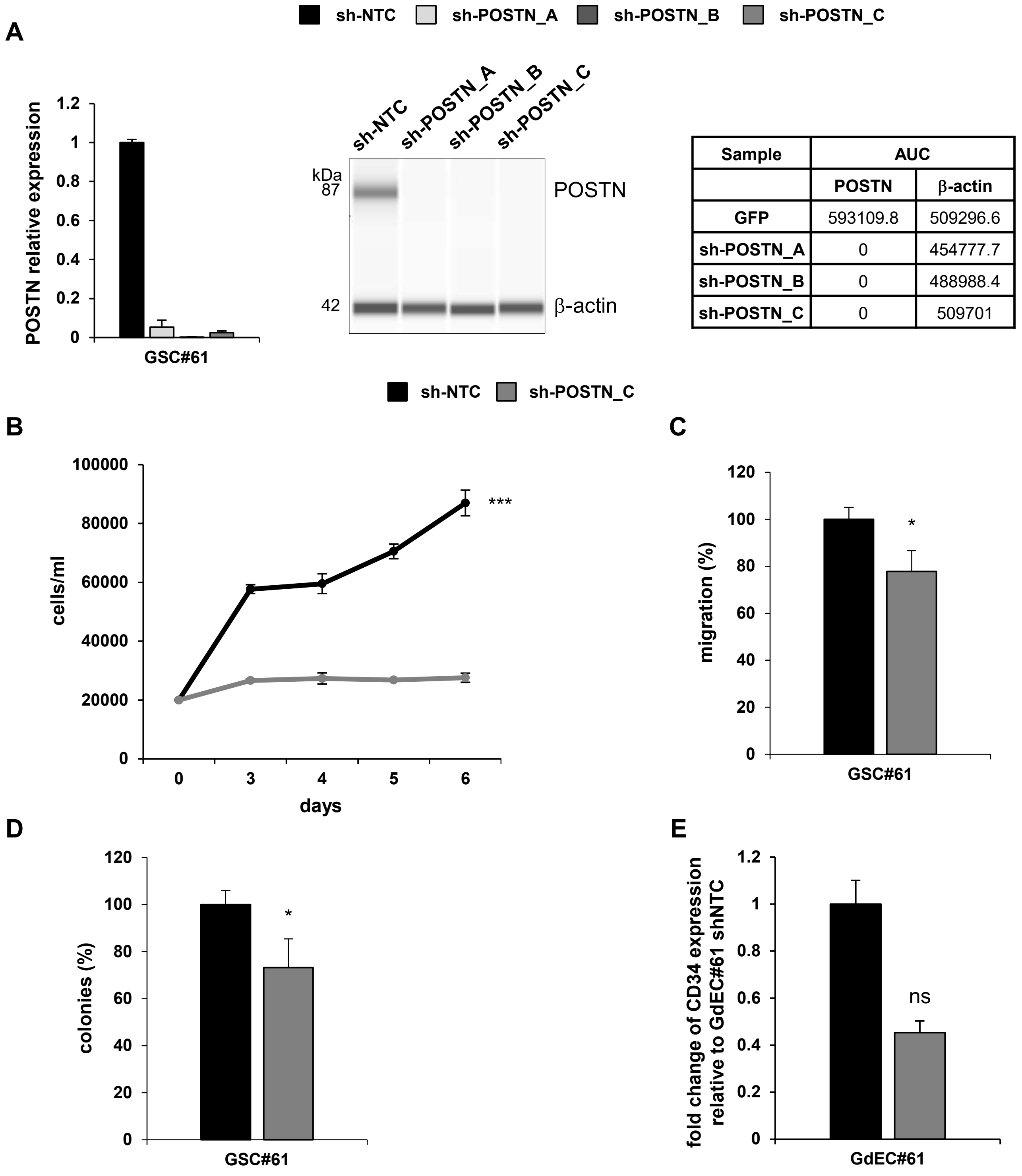

Sample	AUC	
	POSTN	β-actin
GFP	593109.8	509296.6
sh-POSTN_A	0	454777.7
sh-POSTN_B	0	488988.4
sh-POSTN_C	0	509701

Additional file 4: Figure S3. Effects of POSTN silencing on GSC tumorigenic properties and endothelial transdifferentiation potential. A. qRT-PCR analysis (*left*) and WES full-length gel image (*center and right*) of POSTN expression in sh-NTC or sh-POSTN GSC#61. For qRT-PCR, samples were run in duplicate and data were normalized to the GAPDH expression in the corresponding samples. For WES, the relative amount of each immunoreactive band was shown, the protein expression was quantified using AUC measurements and signal intensity was normalized to the β-actin expression in the same sample. **B-D.** Functional *in vitro* assays on sh-NTC or sh-POSTN_C GSC#61. Growth curve (**B**): points and range lines at each day represent mean ± SD of at least two independent experiments in triplicate. Migration (**C**) and Colony formation (**D**) assays: values are reported as percentage relative to sh-NTC GSC#61 and shown as mean ± SD from two independent experiments in triplicate. **E.** Transdifferentiation assay: cytofluorimetric evaluation of CD34 expression. Values are reported as fold change relative to sh-NTC GdEC#61. ns, not significant; *p<0.05; ***p<0.001 *vs* sh-NTC vector. (Student's t test).
