# Supplementary material for: Extracellular vesicles produced by irradiated endothelial or Glioblastoma stem cells promote tumor growth and vascularization modulating tumor microenvironment
Source: Cancer Cell Int. 2024 Feb 12;24:72. doi: 10.1186/s12935-024-03253-0 (PMC10863174; doi:10.1186/s12935-024-03253-0)
Supplement: Supplementary file 5 — Additional file 5: Figure S4. Filamin-B and Periostin expression increases in recurrent GBM patients. Box plot showing the expression of FLNB A and POSTN B in GBM clinical samples (https://xena.ucsc.edu/), GDC TCGA GBM dataset (n=671 samples). Significance (p values) were evaluated by One-way ANOVA. [file 12935_2024_3253_MOESM5_ESM.pdf]

A

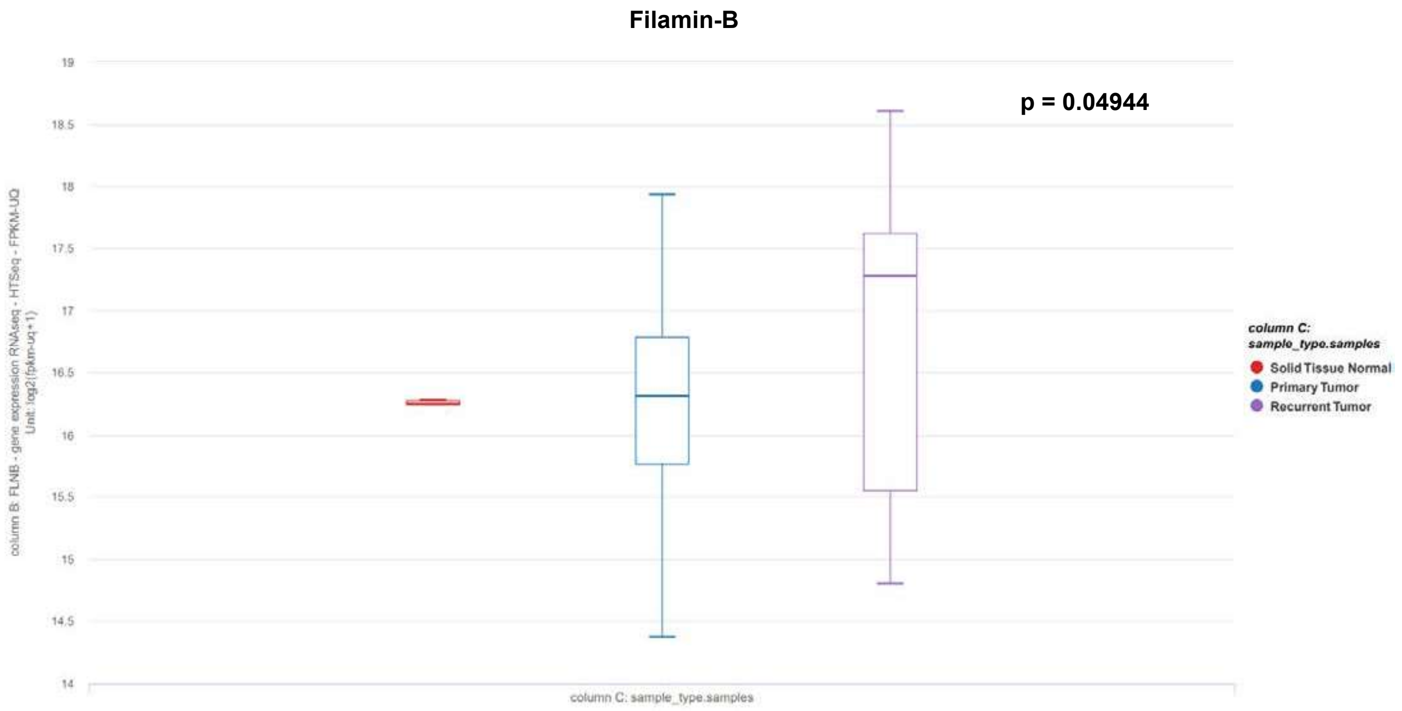


B

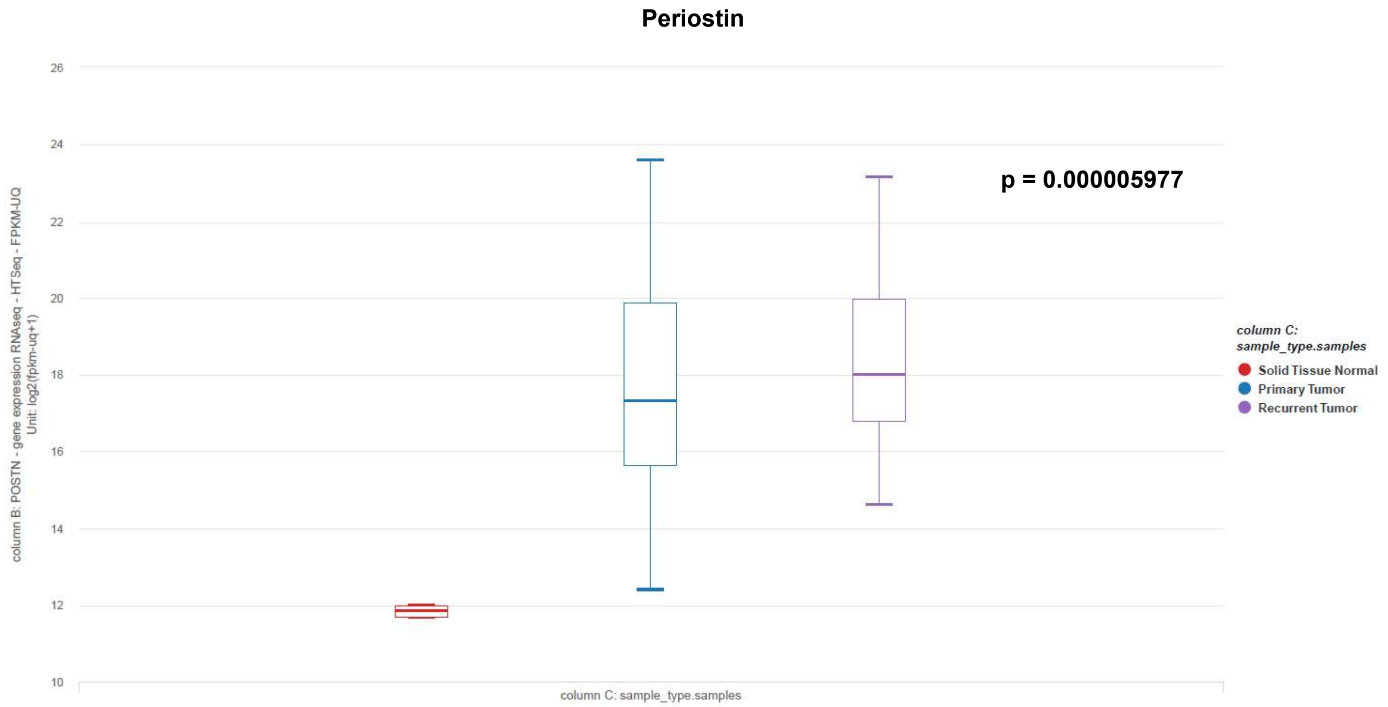


**Additional file 5: Figure S4. Filamin-B and Periostin expression increases in recurrent GBM patients.** Box plot showing the expression of FLNB **(A)** and POSTN **(B)** in GBM clinical samples (https://xena.ucsc.edu/), GDC TCGA GBM dataset (n=671 samples). Significance (p values) were evaluated by One-way ANOVA.
